# Development of a CRISPR/Cas12a genome editing toolbox in *Kluyveromyces marxianus* and its application in succinic acid biosynthesis

**DOI:** 10.1016/j.synbio.2025.09.015

**Published:** 2025-09-16

**Authors:** Hao Zha, Yanjie Li, Zhongmei Hu, Jiacheng Li, Yujie Xie, Mingtao Zhao, Lili Ren, Biao Zhang

**Affiliations:** Anhui Province Key Laboratory of Pollutant Sensitive Materials and Environmental Remediation, School of Life Sciences, Huaibei Normal University, Huaibei, Anhui, 235000, PR China

**Keywords:** CRISPR/Cas12a, *K*. *marxianus*, Genome editing, Succinic acid, Metabolic engineering

## Abstract

*Kluyveromyces marxianus* is a promising thermotolerant yeast for industrial biotechnology, but lacks efficient genome engineering tools. A CRISPR/Cas12a genome editing toolbox for *K*. *marxianus* was developed for the first time in this study. A plasmid-free transient system achieved single-gene knockout efficiencies of about 50 %–100 % in *Δku70* strain. Even with homology arms as short as 35 bp, the knockout efficiency remained 66.67 %. Chromosomal integration of Cas12a enabled single-to-triple fragment knock-ins efficiency of 82.93–85.70 % and 94.50 % for large fragment (>5 kb) integrations. Applying this system, the roles of succinate dehydrogenase (*SDH*) genes *SDH1-SDH5* were elucidated. Combinatorial *SDH* genes knockouts redirected carbon flux toward succinic acid (SA), but increased glycerol/acetate byproducts. Subsequent *GPD1*/*ACH1/ADH2A* co-knockout in a *Δsdh1,3,5,4A,2* strain with *NDE1* overexpression (YZH43) yielded a chassis producing 32.38 g/L SA from glucose at 37 °C, which is the highest reported titer in *K. marxianus*, while reducing ethanol, acetate, and glycerol by 60.79 %, 89.24 %, and 67.5 %, respectively. At 46 °C, YZH43 produced 20.51 g/L SA through simultaneous saccharification and fermentation (SSF) using cellulose as substrate. This work provides a high-efficiency CRISPR/Cas12a platform for *K. marxianus*, enabling rapid metabolic engineering for value-added chemical production, and demonstrates its utility in developing thermotolerant SA-overproducing strains.

## 1 Introduction

*Kluyveromyces marxianus* emerges as a highly promising next-generation cell factory due to its exceptional physiological traits including the fastest-growing eukaryote (doubling time as low as 45 min), exhibits remarkable thermotolerance (up to 52 °C), acid tolerance (pH 2.3), and broad substrate utilization capacity (efficiently metabolizing glucose, lactose, xylose, arabinose, galactose, inulin, pentose sugars, and disaccharides) [1,2]. It's generally recognized as safe (GRAS, US) and qualified presumption of safety (QPS, EU) safety certifications, combined with Crabtree-negative metabolism that minimizes accumulation of ethanol, glycerol, and organic acid byproducts, enhances product yield and separation efficiency [3,4], supporting successful high-level production of diverse compounds including fuel ethanol, 2-phenylethanol, d-allulose, and triacetic acid lactone from non-glucose substrates, alongside secretion of valuable lytic enzymes (β-galactosidase, pectinase, inulinase) [5,6]. *K. marxianus* shares nutritional needs and cultivation methods with *Saccharomyces cerevisiae* and can leverage natural strain diversity for chassis optimization without needing ethanol rerouting. However, it faces significant hurdles as a metabolic engineering platform. Inefficient and random gene targeting makes stable integration of foreign genes difficult. Limited biochemical and genetic understanding hinders rational design. These challenges persist despite ongoing development of gene expression systems, genome engineering tools, and models. These limitations currently constrain its full exploitation across food, feed, and pharmaceutical industries, though its inherent advantages position it as a versatile industrial host [3,7,8].

Current CRISPR/Cas9 genome editing tools for *K*. *marxianus* primarily utilize episomal plasmids or chromosomal integrations for Cas9/gRNA delivery, with episomal systems favored for high transformation efficiency [7,[9], [10], [11], [12], [13], [14], [15], [16], [17]]. However, no studies have yet reported CRISPR/Cas12a-mediated genome editing in *K*. *marxianus*. CRISPR/Cas12a (also known as CRISPR/Cpf1), a Class 2 Type V CRISPR-Cas system, recognizes T-rich PAM sequences (TTTV or TTV, where V = A/C/G) upstream of the short guide RNA target, differing from Cas9's NGG-specific PAM [18,19]. Furthermore, Cas12a exhibits both endonuclease activity for DNA cleavage and RNase activity. Consequently, Cas12a can autonomously process precursor crRNA by identifying short 19-bp direct repeat (DR) sequences and releasing mature crRNAs from precursor transcripts [20]. This enables the design of crRNA arrays containing multiple tandem DR/gRNA units [20]. Cas12a cuts DNA about 14–23 nucleotides downstream of the PAM site; this location is distal to the recognition region. Consequently, small insertions or deletions created by classical non-homologous end joining (NHEJ) repair near this cut site usually do not disrupt the target sequence [18,21]. This allows repeated Cas12a recognition and editing of the same locus, mitigating interference from NHEJ-induced mutations on fragment insertion efficiency and enhancing precision of homology-directed repair (HDR), thereby achieving superior fidelity and specificity. As a next-generation gene editing tool, the CRISPR/Cas12a system exhibits significant advantages in yeast synthetic biology [22,23]. In *Pichia pastoris*, Zhang et al. optimized CRISPR/Cas12a components to achieve single-gene editing efficiencies of 99 ± 0.8 %, double-gene editing efficiencies of 65–80 %, and triple-gene editing efficiencies of 30 ± 2.5 % [24]. They also accomplished, for the first time, the deletion of a large 20 kb fragment and the one-step integration of three genes for the lycopene synthesis pathway, providing efficient tools for chassis genome streamlining and metabolic pathway assembly [24]. In *S*. *cerevisiae*, the sticky-end cleavage characteristic of Cas12a (cleavage sites located ±18/+23 bp distal to the PAM) enhances homologous recombination efficiency [25]. Ciurkot et al. combined with in vivo recombination technology mediated by 50 bp linkers, successfully multiplex integrated β-carotene synthesis genes (*CRTE*, *CRTYB*, *CRTI*) into independent genomic loci validated the reliability of multiplex editing [25]. In *Yarrowia lipolytica*, the Cas12a system was further extended to transcriptional regulation and genome-wide screening. Ramesh et al. developed CRISPR activation/repression vectors and constructed a genome-wide gRNA library, combining machine learning algorithms to predict efficient gRNAs for high-throughput optimization of targeted metabolic pathways. Cas12a offers the advantages of low off-target rates and T-rich PAMs, making it suitable for targeting promoter regions and gene regions with high TA content [26].

In this study, a plasmids-free CRISPR/Cas12a-based gene editing system for *K. marxianus* was developed. In the transient CRISPR/Cas12a system, the knockout efficiency for single genes at multiple loci reached over 90 %, with a minimal requirement of only 35 bp homology arm length. When Cas12a was integrated into the genome of *K. marxianus*, it maintained its high gene editing efficiency, achieving in vivo recombination and knock-in efficiencies of over 80 % for single, double, and triple exogenous DNA fragments. The in vivo recombination and knock-in efficiencies for 5 kb and 1.2 kb fragments reached as high as 94.50 %. Leveraging this technology, we systematically elucidated the function of the succinate dehydrogenase (*SDH*) genes in *K. marxianus* and constructed an engineered *K. marxianus* strain capable of synthesizing succinic acid from glucose. This study will provide a foundation for fully exploring the industrial application potential of *K. marxianus*.

## 2 Materials and methods

### 2.1 Microorganisms and media

All chemicals used were of analytical grade or higher. The DNA polymerase and seamless cloning kit was purchased from Tolo Biotech Co. (Chuzhou, Anhui, China). Ligase, recombinase restriction enzymes (*EcoR*I, *Sma*I, *Sac*I, and *Not*I) were obtained from Sangon Biotech Co. (Shanghai, China). Glucose, inulin, glycerol, ethanol, acetic acid, uracil, adenine, and yeast nitrogen base (YNB) were also purchased from Sangon Biotech. Yeast extract and peptone were sourced from Angel (Yichang, Hubei, China). The *K. marxianus* strain NBRC1777 served as the original strain. YZB040 is a *URA3*-deficient derivative of NBRC1777, YZB100 is a *KU70*-deficient derivative of NBRC1777, and YZB101 is a *URA3*-deficient derivative of YZB100. *K. marxianus* was cultured in YPD medium (20 g/L glucose, 10 g/L yeast extract, 20 g/L peptone). YPI medium was prepared by replacing glucose in YPD with inulin, while keeping other components identical. Yeast transformants were selected on synthetic dropout (SD) (0.67 % YNB and 2 % glucose), supplemented as required. The *URA3* marker was recycled by transforming cells with a truncated *URA3* fragment, and transformants were selected on SD plates supplemented with 0.1 % 5-fluoroorotic acid (5′-FOA) and uracil. The SI medium is prepared by replacing glucose in the SD medium with inulin. The SD + Adenine medium is prepared by adding 5 μg/mL adenine to the SD medium. *Escherichia coli* DH5α, used for plasmid construction, was cultured in lysogeny broth (LB) medium. Bacterial transformants were screened on solid medium (1.5 % agar) supplemented with 100 μg/mL ampicillin.

### 2.2 Plasmids and strains construction

Primers, plasmids, strains, genes used in this study are summarized in Tables S1, S2, S3, and S4 p414-TEF1p-Cas9-CYC1t (Addgene 43802) was obtained from Addgene. The *FnCas12a* gene from *Francisella novicida* was codon-optimized, cloned into the p414-TEF1p-Cas9-CYC1t using a seamless cloning kit (Sangon Biotech Co., Shanghai, China), and replaced the original *SpCas9* gene, resulting in the plasmid pZB214. pZB211 that containing the fragment *P*_*ScSNR52*_-DR was constructed by PCR with p426-SNR52p-gRNA.CAN1.Y-SUP4t (Addgene 43803) as template using primers 5′-phosphorylation 426P-F and 426P-R following ligation and transformation. Similarly, pZB212 which containing the fragment, DR-*T*_*ScCYC1*_ was also constructed by PCR with p426-SNR52p-gRNA.CAN1.Y-SUP4t as template using primers 426T-F and 5′-phosphorylation 426T-R following ligation and transformation. pZB211 and pZB212 served as templates for constructing crRNA through fusion PCR. The plasmid PZB214 was double-digested with restriction enzymes *Sma*I and *Sac*I. The *P*_*KmINU1*_, *P*_*KmGDH2*_, and *P*_*KmPDC1*_ promoter fragments were amplified from the *K. marxianus* genome, replaced the *P*_*ScTEF1*_ promoter in PZB214 (double-digested with *Sma*I and *Sac*I) using a seamless cloning kit, yielding plasmids pZB260, pZB261, and pZB273. The *P*_*KmSNR52*_, *P*_*KmTEF1*_, and *P*_*KmtRNA*_^*gly*^ fragments were amplified from the *K. marxianus* genome, substituted for the *P*_*ScSNR52*_ promoter in the pZB211 plasmid using a seamless cloning kit, resulting in plasmids pZB281, pZB282, and pZB283. The plasmid pMD18T-*ΔScURA3* was used to amplify a truncated *ScURA3* fragment for the *URA3* selection marker. The plasmid pZB023 was employed to construct knockout cassettes containing the *URA3* tag and homologous arms of the gene to be knocked out (Table S2). The *KmNDE1*coding sequence was amplified from the genomic DNA of *K. marxianus* and cloned into the *EcoRI*/*Not*I sites of pZB023, resulting in plasmid pZB047. The *KmRAD52* coding sequence was amplified from the genomic DNA of *K. marxianus* and cloned into the *EcoRI*/*Not*I sites of pZB023, resulting in plasmid pZB182. Similarly, the *EGFP* coding sequence was amplified from plasmid pCG [27] and cloned into the *EcoR*I/*Not*I sites of pZB023, resulting in plasmid pZB191. The *Sp*Cas9^D10A, H840A^ mutant was constructed using plasmid p414-TEF1p-Cas9-CYC1t and designated as pZB210.

Overexpression of *KmRAD52* at *XYL2* site in YZB101 using transient CRISPR/Cas9 [17] generated strain YZB358. Disruption of *URA3* in YZB358 then yielded strain YZB359. CAS12a expression cassettes driven by the *P*_*KmINU1*_, *P*_*KmGDH2*_, *P*_*KmTEF1*_, and *P*_*KmPDC1*_ were individually integrated into the *XYL1* locus of YZB101, producing strains YZB567, YZB568, YZB569, and YZB596. Subsequently, *URA3*-deficient derivatives were constructed from these strains to generate strains YZB570, YZB571, YZB572, and YZB599.

### 2.3 The *K. marxianus ADE2* disruption efficiency in WT and *Δku70* strain using transient CRISPR/Cas12a system

**Fig. 1:**
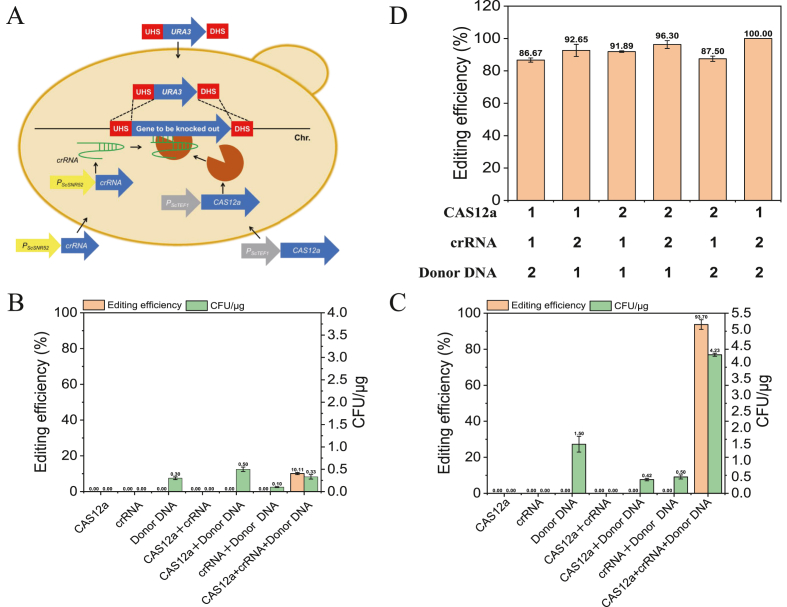
(A) Schematic representation of the transient CRISPR/Cas12a-based genome editing system in *K. marxianus* (UPS/DHS: upstream/downstream homologous sequences). (B) The transient CRISPR/Cas12a-based genome editing system in *ku70* non-knockout strain. (C) The transient CRISPR/Cas12a-based genome editing system in *ku70* knockout strain. (D) *ADE2* disrupted efficiencies of 40 or 50 μg of DNA fragments of Cas12a expression cassette, crRNA transcription cassette, and donor DNA with mass ratios of 1:1:2, 1:2:1, 2:1:1, 2:2:1, 2:1:2, and 1:2:2.

The *CAS12a* was PCR amplified from the plasmid pZB214 with primers M13-F/M13-R. The crRNA was designed using the online Cas-Designer server (http://www.rgenome.net/cas-designer/). The crRNA was constructed through overlap-extension PCR with pZB211and pZB212 as a template using primers SNR52P-SF/ADE2-SNR52-R1 and ADE2-CYC1-F1/CYC1T-SR, similar to the previous report [17]. The crRNA-*ADE2* guide RNA sequence directs Cas12a activity to the *ADE2* site. *URA3* flanked by *ADE2* sequences of 60 bp arms at both ends, were amplified from pZB023 with primers ADE2-60-F/ADE2-60-R, served as donor DNA to replace the *ADE2* target gene (Fig. 1A). Next, 30 μg of DNA fragments, including the three components (Cas12a expression cassette, crRNA transcription cassette, and donor DNA) with mass ratio of 1:1:1, were transformed into YZB040 and YZB101 (*ku70* defective strain) strains either individually, in pairwise combinations, or all together, selected on an SD + Adenine plate for the uracil-independent transformants. Knockout of the *ADE2* gene was determined by the change in colony color (colonies turned red upon successful *ADE2* deletion). After confirming the color change through streak purification, genomic PCR verification was performed. Furthermore, 40 or 50 μg of DNA fragments of Cas12a expression cassette, crRNA transcription cassette, and donor DNA with mass ratio of 1:1:2, 1:2:1, 2:1:1, 2:2:1, 2:1:2, and 1:2:2 were transformed into YZB101, respectively, to determine the effects of ratios of these components on the gene editing efficiency. Unless otherwise specified, a total of 30 μg of fragments was used in subsequent transformations, with the three fragments mixed at a 1:1:1 mass ratio, all data are shown as the mean ± standard deviation from at least three experiments.

### 2.4 Evaluation of PAM sites, homology arm length, and temperature on transient CRISPR/Cas12a editing efficiency in *K. marxianus*

To assess the impact of PAM sites on CRISPR/Cas12a editing efficiency in *K*. *marxianus*, crRNAs targeting TTG, TTC, TTA, and TTT PAM sites were constructed. Similar to the construction of cr-Ade2 described in Section 2.3, which targeted the TTG PAM site, crRNAs targeting TTC, TTA, and TTT were generated by replacing primers ADE2-SNR52-R1/ADE2-CYC1-F1 with ADE2-SNR52-R2/ADE2-CYC1-F2, ADE2-SNR52-R3/ADE2-CYC1-F3, and ADE2-SNR52-R4/ADE2-CYC1-F4, respectively. Furthermore, using the crRNA targeting the TTC PAM site (which showed the highest efficiency) along with Cas12a and donor DNA templates containing varying homology arm lengths, the effect of homology arm length on CRISPR/Cas12a editing efficiency was evaluated. Additionally, the influence of temperature (30 °C, 37 °C, and 45 °C) on transient CRISPR/Cas12a genome editing efficiency in *K. marxianus* was investigated. All data are shown as the mean ± standard deviation from at least three experiments.

### 2.5 Impact of genomic loci on transient CRISPR/Cas12a editing efficiency in *K. marxianus*

In addition to *ADE2*, genes involved in xylose metabolism (*XYL1* and *XYL2*), and *TRP1* were selected as targets to assess the knockout efficiency of transient CRISPR/Cas12a. CrRNAs targeting these loci were generated by fusion PCR. These crRNAs, along with the *CAS12a* expression cassette and donor repair fragments (containing 60-bp homology arms amplified using plasmid pZB023 as template), were co-transformed into strain YZB101. Putative *XYL1* and *XYL2* knockout strains were initially screened on SX plates (YNB + 2 % xylose), as deletion of these genes results in loss of growth capability on this medium. Final confirmation was achieved by genomic PCR. For *TRP1* knockouts, verification was performed directly by genomic PCR. All data are shown as the mean ± standard deviation from at least three experiments.

### 2.6 Impact of promoters controlling *Cas12a* expression on transient CRISPR/Cas12a editing efficiency in *K*. *marxianus*

Since *CAS12a* expression in the above gene editing experiments was driven by the *ScTEF1* promoter (*P*_*ScTEF1*_), we investigated whether using endogenous *K. marxianus* promoters to control *CAS12a* expression would enhance editing efficiency. The weak constitutive promoter *P*_*KmGDH2*_, the strong constitutive promoter *P*_*KmPDC1*_, and the inulin-inducible promoter *P*_*KmINU1*_ from *K. marxianus* were selected to regulate *CAS12a* expression. The *ADE2* locus was uniformly targeted for knockout in all experiments to facilitate efficiency quantification via colony color phenotype. For the *P*_*KmINU1*-_controlled *CAS12a* system, editing efficiency was assessed after culturing transformants on both SD + Adenine plates (non-induced condition) and SI + Adenine plates (inulin-induced condition).

The relative expression levels of *CAS12a* controlled by different promoters were determined using real-time quantitative PCR (RT-qPCR). The primers used are shown in Table S1. Strains YZB567, YZB568, YZB569, and YZB596 were pre-cultured in YPD medium overnight and transformed to YPD medium cultured for 4 h at 37 °C. Besides, strain YZB567 was pre-cultured in YPD transformed to YPI, YPDI, YP1D3I, and YP3D1I medium, cultured for 4 h at 37 °C. After that, the total RNA was isolated using a yeast total RNA extraction kit (Sangon Biotech Co. Shanghai, China). Then, the isolated RNA was treated with RNase-free DNase I (Sangon Biotech Co. Shanghai, China) at 37 °C for 15 min to remove the potentially contaminated genomic DNA. cDNA was synthesized by a reverse transcription kit (Sangon Biotech Co. Shanghai, China). The reverse transcription reaction was performed in an Arktik thermal cycler (Thermo Fisher Scientific, West Palm Beach, FL) at 37 °C for 15 min, 50 °C for 5 min, and denatured at 98 °C for 5 min. The synthesized cDNA was quantitatively determined using a Nanodrop 2000 (Thermo Fisher Scientific, West Palm Beach, Florida, USA). Real-time PCR was conducted on a Light Cycler 480 (Roche Diagnostics Ltd., Forrentrasse CH-6343 Rotkreuz, Switzerland) with qPCR mix kit (Sangon Biotech Co. Shanghai, China). The relative mRNA expression was quantified according to the formula of 2^−ΔΔCT^. Gene *KmACT1* for actin was used as an internal control. The primers for the real-time qPCR are shown in Table S1. All data are shown as the mean ± standard deviation from at least three experiments.

### 2.7 Impact of *KmRAD52* overexpression on transient CRISPR/Cas12a editing efficiency in *K*. *marxianus*

*RAD52* is a recombinase that enhances the efficiency of homologous recombination. Strain YZB358 was derived from YZB101 by overexpressing *KmRAD52*, and YZB359 is a *URA3*-marked derivative of YZB358. The *CAS12a* expression cassettes driven by *P*_*ScTEF1*_, *P*_*KmGDH2*_, and *P*_*KmPDC1*_ were individually co-transformed with the crRNA targeting *ADE2* and the donor repair fragment (containing 60-bp homology arms) into strain YZB359. Editing efficiency was quantified by counting red colonies resulting from successful *ADE2* knockout. All data are shown as the mean ± standard deviation from at least three experiments.

### 2.8 Impact of integrated CRISPR/Cas12a on genome editing efficiency in *K*. *marxianus*

To evaluate the effect of integrated CRISPR/Cas12a systems on genome editing efficiency in *K. marxianus*, *CAS12a* expression cassettes driven by *P*_*ScTEF1*_, *P*_*KmGDH2*_, *P*_*KmPDC1*_, and *P*_*KmINU1*_were individually integrated into the *XYL1* locus of strain YZB101. Using *ADE2* as the target gene and donor DNA with 60-bp homology arms, editing efficiency was assessed. To enhance the editing efficiency of the *P*_*KmINU1*_ controlled integrated *CAS12a* system, the effects of different pre-culture media, including YPD, YPI, YPDI (1 % glucose + 1 % inulin), YP1D3I (0.5 % glucose + 1.5 % inulin), and YP3D1I (1.5 % glucose + 0.5 % inulin) were compared. Additionally, editing efficiency was tested after culturing transformants on both SD + Adenine plates (non-induced) and SI + Adenine plates (inulin-induced). All data are shown as the mean ± standard deviation from at least three experiments.

### 2.9 Assessing the toxicity of integrated CRISPR/Cas12a in *K*. *marxianus*

Given the inherent cellular toxicity of Cas12a, the impact of integrated Cas12a on *K. marxianus* growth was evaluated. Strains harboring integrated Cas12a expression cassettes driven by *P*_*ScTEF1*_, *P*_*KmGDH2*_, *P*_*KmPDC1*_, and *P*_*KmINU1*_, along with the wild-type (WT) strain, were cultured in liquid YPD and SD media at 37 °C with 220 rpm shaking. Growth curves were compared to assess toxicity. Additionally, growth curves of the *P*_*KmINU1*_-*CAS12a* integrated strain and WT cultured in liquid YPI and SI media under identical conditions (37 °C, 220 rpm) were analyzed to evaluate toxicity under both constitutive and inducible conditions. All data are shown as the mean ± standard deviation from at least three experiments.

### 2.10 Efficiency of single, double, and triple fragment knock-in in *K. marxianus* using integrated CRISPR/Cas12a

To evaluate the knock-in efficiency of an integrated CRISPR/Cas12a system in *K. marxianus*, a DNA fragment containing an *EGFP* expression cassette and a *URA3* selectable marker flanked by 40-bp homology arms was amplified. This fragment was targeted to the *ADE2* locus. For one-step double-fragment knock-in, the *EGFP* and *URA3* cassettes were split into two separate fragments. These fragments shared a 40-bp overlapping region within the *EGFP* coding sequence and were co-targeted to the *ADE2* locus. For one-step triple-fragment knock-in, the cassettes were divided into three fragments. The first overlapping region matched that used in the double-fragment approach. A second 40-bp overlapping region was designed at the junction between the *EGFP* and *URA3* cassettes. All fragments were targeted to the *ADE2* locus. Successful fragment integration efficiency was calculated based on the percentage of red colonies (indicating *ADE2* knockout). *EGFP* expression efficiency was assessed by counting cells exhibiting green fluorescence under fluorescence microscopy. The imaging data were collected using a Motic PA53 FS6 Spinning Disk Microscope equipped with a Motic Images Plus 3.0 camera and a HPlan S-APO 4×–100× objective (Motic China Group Co., LTD, Xiamen, China). The excitation wavelength for EGFP was 488 nm. All data are shown as the mean ± standard deviation from at least three experiments.

### 2.11 Large-fragment (>5 kb) knock-in efficiency in *K*. *marxianus* using integrated CRISPR/Cas12a

Plasmid pZB210 contains a ∼5-kb expression cassette for *dCAS9*. To integrate this *dCAS9* cassette into the *K. marxianus* genome, a *URA3* selectable marker cassette was also required. The combined fragments (*dCAS9* cassette and *URA3* marker) were co-transformed into strains harboring integrated *P*_*ScTEF1*-_*CAS12a* or *P*_*KmPDC1*_-*CAS12a* systems. This transformation utilized a crRNA targeting the *ADE2* locus and driven by the *P*_*ScSNR52*_ promoter. Additionally, crRNAs targeting *ADE2* and controlled by *P*_*KmSNR52*,_ *P*_*KmTEF1*_, or *P*_*KmtRNA*_^*gly*^ promoters were individually co-transformed with the donor repair fragment into the strain harboring integrated *P*_*KmPDC1*_-*CAS12a*. All data are shown as the mean ± standard deviation from at least three experiments.

### 2.12 Impact of crRNA promoters on knock-out efficiency using integrated CRISPR/Cas12a in *K*. *marxianus*

Given the influence of crRNA promoters on large-fragment knock-in efficiency, whether substituting the crRNA promoter could further enhance editing efficiency was investigated. CrRNAs targeting *ADE2* and controlled by the *P*_*KmSNR52*_, *P*_*KmTEF1*_, or *P*_*KmtRNA*_^*gly*^ promoters were individually co-transformed with the donor DNA fragment into the strain harboring integrated *P*_*KmPDC1*_-*CAS12a*. Knockout efficiencies were quantified based on the number of red colonies. All data are shown as the mean ± standard deviation from at least three experiments.

### 2.13 The effect of *SDH* genes knockout in *K*. *marxianus* on succinic acid production

Using the CRISPR/Cas12a system, based on the YZB599 strain and employing *P*_*KmSNR52*_ to control crRNA expression, single-gene knockout strains for *SDH1*, *SDH2*, *SDH3*, *SDH4A*, *SDH4B*, AND *SDH5* were constructed. Phenotypic experiments on SD plates were used to investigate the effects of *SDH* genes knockout on the growth of *K. marxianus* at 30 °C, 37 °C, and 46 °C. Growth of *SDH4A* deletion strain was performed using SD medium at 37 °C and 46 °C. Using an Erlenmeyer flask with YPD and YPG media at 37 °C and 220 rpm, the impact of *SDH* genes knockout on succinic acid production by *K*. *marxianus* were tested.

Furthermore, strains with different combinations of *SDH* genes knockouts (*Δsdh3Δsdh4*, *Δsdh3Δsdh4AΔsdh4B*, *Δsdh3Δsdh4AΔsdh4BΔsdh5*, *Δsdh3Δsdh4AΔsdh4BΔsdh5Δsdh2*, *Δsdh3Δsdh4AΔsdh4BΔsdh5Δsdh1*, *Δsdh3Δsdh4AΔsdh4BΔsdh5Δsdh1Δsdh2*, *Δsdh1Δsdh3Δsdh5*, *Δsdh1Δsdh3Δsdh5Δsdh4A*, and *Δsdh1Δsdh3Δsdh5Δsdh4AΔsdh2*) were constructed. Their abilities to synthesize succinic acid were investigated using YP10D medium (YP medium supplemented with 100 g/L glucose) under conditions of 37 °C and 220 rpm. The concentrations of d-glucose, succinic acid, fumarate, malate, ethanol, acetate, and glycerol were analyzed as described previously [28]. All data are shown as the mean ± standard deviation from at least three experiments.

### 2.14 Reactive oxygen species (ROS) quantification assay

The *SDH* single-gene knockout strain was pre-cultured overnight in YPD medium at 37 °C. Cells were then transferred to SD medium and incubated at 30 °C and 46 °C for 12 h, respectively. Intracellular ROS levels were measured using an ROS assay kit (ShareBio, Shanghai, China). 2′,7′-dichlorodihydrofluorescein diacetate (DCFH-DA), a non-fluorescent probe, freely penetrates live cell membranes and is hydrolyzed by intracellular esterases to form 2′,7′-dichlorodihydrofluorescein (DCFH). DCFH remains non-fluorescent and membrane-impermeable, and is oxidized by ROS to generate fluorescent 2′,7′-Dichlorofluorescein (DCF). Based on fluorescence generation in live cells, ROS levels and dynamics can be determined. Qualitative analysis was performed using fluorescence microscopy (Motic China Group Co., LTD, Xiamen, China), while quantitative measurement was conducted with a microplate reader (excitation: 504 nm; emission: 529 nm) (SPECTRAmax GEMINIXs, Molecular Devices, Union City, CA, USA). Rosup is a ROS-inducing agent that served as the positive control. All data are shown as the mean ± standard deviation from at least three experiments.

### 2.15 The effect of *ACH1* and *GPD1* knockout to the glycerol and acetate accumulation in succinic acid-producing *K*. *marxianus*

Since the knockout of *SDH* genes diverts metabolic flux towards acetate and glycerol, leading to undesirable accumulation of glycerol, acetate, and ethanol, strains *Δach1*, *Δach1Δgpd1*, *Δsdh1Δsdh3Δsdh5Δsdh4AΔsdh2Δach1*, *Δsdh1Δsdh3Δsdh5Δsdh4AΔsdh2Δach1Δgpd1*, *Δach1Δgpd1Δsdh3*, and *Δach1Δgpd1Δsdh1* were further constructed. Their succinic acid synthesis using YP10D medium and the accumulation of byproducts, including ethanol, glycerol, and acetate, were investigated under conditions of 37 °C and 220 rpm. All data are shown as the mean ± standard deviation from at least three experiments.

### 2.16 The effect of *ADH* genes knockout on the ethanol accumulation in succinic acid-producing *K*. *marxianus*

*ADH* genes (*ADH1 ADH2A*, *ADH2B*, *ADH3)* in YZH35 **(***Δsdh1Δsdh3Δsdh5Δsdh4AΔsdh2Δach1Δgpd1*) were deleted with transient CRISPR/Cas12a to obtained strains YZH39, YZH40, YZH41 and YZH42. *KmNED1* was overexpressed in YZH36 at *ADH2A* site to obtain strains YZH43. Their SA synthesis using YP10D medium and the accumulation of byproducts, including fumarate, malate, ethanol, glycerol, and acetate, were investigated under conditions of 37 °C and 220 rpm. Adaptive laboratory evolution (ALE) was employed to restore growth defects caused by *SDH* and *ADH* gene knockouts. Engineered strains were cultured in YPD medium until reaching OD_600_ > 15 prior to subculturing. The initial transfer volume was set at 10 %, which was gradually reduced by 1 % per passage over a total of 11 serial transfers. All data are shown as the mean ± standard deviation from at least three experiments.

### 2.17 Measurement of the intracellular NADH/NAD^+^ contents

Intracellular NADH/NAD ^+^ contents of *K. marxianus* YZB596, YZH33, YZH31, YZH35, YZH37, YZH38, YZH40-ALE, and YZH43 were extracted from cells cultivated in YPD medium. A 1 mL sample of yeast culture was withdrawn and washed with sterile water. Then, the intracellular NADH/NAD^+^ contents were extracted using an NAD^+^/NADH Assay Kit (Macklin, Shanghai, China) following the manufacturer's instruction. The WST-8-based colorimetric method quantifies cellular NAD^+^ and NADH levels through a chromogenic reaction. During this process, NAD^+^ is reduced to NADH, which subsequently reduces WST-8 to generate orange-yellow formazan. This formazan exhibits an absorption maximum at approximately 450 nm. The amount of formazan produced in the reaction system is directly proportional to the total NAD^+^/NADH content in the sample.

### 2.18 Synthesis of SA using cellulose as substrate

A solution containing 100 g/L cellulose and 15 FPU/g of cellulose cellulase was hydrolyzed at both 30 °C and 46 °C, followed by quantification of the resulting glucose content. YZH43 strains were fermented using YP medium supplemented with 100 g/L cellulose and 15 FPU/g of cellulose cellulase at 30 °C and 46 °C, respectively, to compare succinic acid production through SSF. All data are shown as the mean ± standard deviation from at least three experiments.

## 3 Results and discussion

### 3.1 Development and characterization of a transient CRISPR/Cas12a-based genome editing system in *K. marxianus* in WT and the *Δku70* strain

A transient CRISPR/Cas12a-based genome editing system was developed and characterized in *K*. *marxianus* (Fig. 1A). YZB040 and YZB101, which are the *URA3* and *URA3*/*KU70* gene defective strains of *K. marxianus* NBRC1777, were used for gene transformation. To facilitate the screening of positive transformants with successful knockouts, the *ADE2* gene, encoding phosphoribosylaminoimidazole carboxylase, was selected as the target site because editing leads to the accumulation of oxidized 5-aminoimidazole ribonucleotide, resulting in red colonies (Fig. S1A) [29]. In addition to calculating the number of red colonies on the transformation plates to determine positive clone efficiency, further verification was performed through streak purification and PCR identification of extracted genomic DNA, with results consistent with those obtained from red colony counting (Fig. S1B and C). The Cas12a protein from *F*. *novicida* was codon-optimized and synthesized according to the codon preference of *K. marxianus*, and the plasmid pZB214 was constructed. A crRNA fragment targeting the *ADE2* gene was constructed by fusion PCR using plasmid pZB211 (containing the *P*_*ScSNR52*_-DR fragment) and plasmid pZB212 (containing the DR- *T*_*ScCYC1*_ fragment) (Fig. S2). Using pZB023, a donor repair fragment was amplified with primers containing 60-bp homology arms for the *ADE2* gene. The three components (the Cas12a expression cassette, the crRNA transcription cassette, and the donor DNA) were transformed either individually, in pairs, or all together into strain YZB040 or YZB101, with 10 μg of each component. As shown in Fig. 1B, when *CAS12a* alone or crRNA alone was transformed, 0 colonies grew. When donor DNA alone was transformed, 0.30 CFU/μg (all white colonies) grew. When *CAS12a* and crRNA were co-transformed, 0 colonies grew. When *CAS12a* and donor DNA were co-transformed, 0.50 CFU/μg (all white colonies, no red colonies) grew. When crRNA and donor DNA were co-transformed, 0.10 CFU/μg (all white colonies, no red colonies) grew. Therefore, no successful gene editing occurred when the *CAS12a* fragment, *crRNA* fragment, or donor DNA fragment was transformed individually or in pairwise combinations. Only when all three components were co-transformed did editing occur, yielding 0.33 CFU/μg transformants with an editing efficiency of 10.11 % (Fig. 1B). In the *Δku70* strain (YZB101), Cas12a alone yielded 0 colonies; crRNA alone yielded 0 CFU/μg; donor DNA alone yielded 1.50 CFU/μg (white colonies); Cas12a + crRNA yielded 0 colonies; Cas12a + donor DNA yielded 0.42 CFU/μg (white colonies); crRNA + donor DNA yielded 0.50 CFU/μg (white colonies). Similarly, no successful gene editing occurred with individual or pairwise transformations. However, when all three components were co-transformed, the *ADE2* knockout phenotype (red colonies) was observed, with editing efficiency dramatically increasing to 93.70 % in the *ku70Δ* strain (Fig. 1C). Ku70 is a core protein in the NHEJ pathway that recognizes and binds to DNA double-strand breaks. Its absence incapacitates this rapid yet error-prone repair mechanism. When cells experience DNA double-strand breaks, the inability to utilize NHEJ forces them to rely entirely on the high-fidelity homologous recombination pathway for repair. The observed increase in the frequency and efficiency of HR usage results from an altered competitive landscape between DNA repair pathways [10]. The *KU70* gene plays a highly significant role in promoting homologous recombination (HR) in *K. marxianus*, consistent with previous reports that its deletion reduces NHEJ and enhances DNA repair mediated by homologous recombination [17]. Furthermore, 40 or 50 μg of DNA fragments of Cas12a expression cassette, crRNA transcription cassette, and donor DNA with mass ratios of 1:1:2, 1:2:1, 2:1:1, 2:2:1, 2:1:2, and 1:2:2 were used to determine the effects of ratios of these components on the gene editing efficiency. As shown in Figs. 1D and 100 % gene knockout efficiency was achieved under the 1:2:2 condition. Overall, the knockout efficiency of transient CRISPR/Cas12a in *K. marxianus* ranged from 50 % to 100 % (Fig. 1D and S3).

### 3.2 Effects of PAM sites, homology arm length, and temperature on transient CRISPR/Cas12a editing efficiency in *K*. *marxianus*

**Fig. 2:**
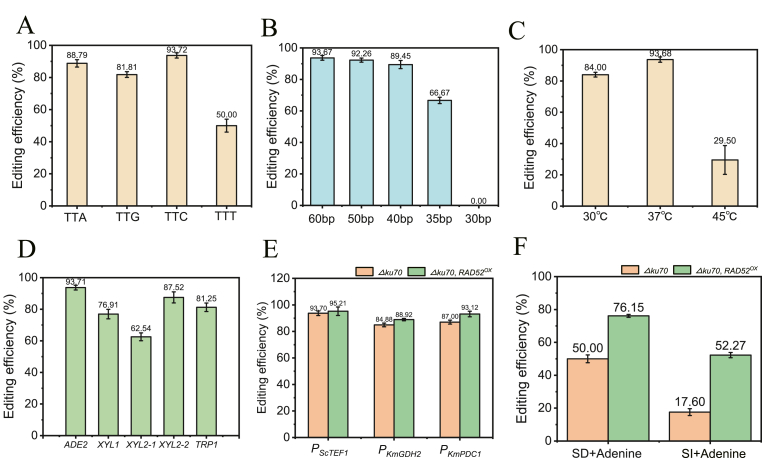
Impact of PAM site (A), homologous arm length (B), pre-culture temperature (C), genomic locus (D), Cas12a promoter strength (E), and Rad52 (F) on transient CRISPR/Cas12a editing efficiency in *K*. *marxianus*.

Previous studies have indicated that gene editing efficiency is influenced by the crRNA location and the PAM sequence (TTN) recognized by the Cas12a/crRNA complex [30]. To evaluate the performance of transient CRISPR/Cas12a in *K*. *marxianus*, different PAM sequences were designed to target corresponding sites within the *ADE2* gene. Four rationally selected crRNAs were designed to target the *ADE2* gene, with PAM sequences TTG, TTC, TTA, and TTT, respectively; the positions of the crRNAs and PAMs in the *ADE2* gene are shown in Fig. S4. The results demonstrated editing efficiencies of 88.79 % for crRNA-TTG, 81.81 % for crRNA2-TTC, 93.72 % for crRNA3-TTA, and 50.00 % for crRNA4-TTT, where crRNA-TTT exhibited a slightly lower efficiency while the other three sites achieved efficiencies above 80 % (Fig. 2A). Certainly, the relatively low knockout efficiency observed with crRNA-TTT may not only result from the influence of the PAM sequence (TTT), but could also be attributed to the positional effect of the crRNA.

The length of the homology arms in the donor DNA significantly impacts the efficiency of homologous recombination repair, with longer arms generally yielding higher editing efficiency. Therefore, donor DNA repair fragments with varying homology arm lengths were designed, selected crRNA-TTA, and the effect of arm length on transient CRISPR/Cas12a gene editing in *K*. *marxianus* was compared. As shown in Fig. 2B, no gene knockout occurred when the homology arm length was less than 30 bp; an editing efficiency of 66.67 % was achieved at 35 bp; this increased to 89.45 % at 40 bp; reached 92.26 % at 50 bp; and peaked at 93.67 % with 60-bp arms. While editing efficiency increased with longer homology arms, the improvement became marginal beyond 40 bp (Fig. 2B). 35-bp homology arm represents the minimal length required for efficient editing in *K*. *marxianus*, equivalent to that of best results reported in *S*. *cerevisiae* (Table S5) [15,16,31].

Given that *K. marxianus* is a thermotolerant yeast capable of growth at temperatures up to approximately 48 °C, we investigated whether *CAS12a* expression fluctuates with temperature changes, thereby affecting gene editing efficiency. Using crRNA-TTA and 60-bp homology arms to target the *ADE2* gene, we tested editing efficiency at three pre-culture temperatures (30 °C, 37 °C, and 45 °C), with transformed plates subsequently cultured at 37 °C. Results revealed that 37 °C is not only the optimal growth temperature for the yeast but also yielded the highest editing efficiency. When the temperature was reduced to 30 °C, editing efficiency decreased by approximately 10 %–84 %, while at 45 °C, *FnCAS12a* editing efficiency dropped significantly to 29.50 %, indicating normal *CAS12a* expression at 37 °C but compromised stability at 45 °C (Fig. 2C).

### 3.3 The transient CRISPR/Cas12a system demonstrates broad applicability for gene editing at diverse loci in *K*. *marxianus*

To investigate whether this system exhibits wide-ranging utility for targeting different genomic locations beyond the *ADE2* gene, four additional loci (*XYL1* and two sites within *XYL2* (*XYL2*-1 and *XYL2-*2) (Fig. S5 and S6) involved in xylose metabolism, along with *TRP1* (Fig. S7) were tested. As shown in Fig. 2D, the knockout efficiencies for *XYL1*, *XYL2*-1, *XYL2*-2, and *TRP1* were 76.91 %, 62.54 %, 87.52 %, and 81.25 %, respectively, all maintained at relatively high levels around 80 % (Fig. 2D). This confirms the broad applicability of transient CRISPR/Cas12a for editing diverse genomic locations in *K. marxianus.*

### 3.4 The influence of *Cas12a* promoter strength and *KmRAD52* on gene editing efficiency in *K*. *marxianus*

To investigate the impact of *CAS12a* promoter strength on gene editing efficiency in *K*. *marxianus*, the original promoter *P*_*ScTEF1*_ was replaced with the constitutive weak promoter *P*_*KmGDH2*_, the constitutive strong promoter *P*_*KmPDC1*_, and the inulin-inducible promoter *P*_*KmINU1*_. Using crRNA-TTA targeting the *ADE2* locus, the effects of these promoters were evaluated. As shown in Fig. 2E and F, neither the constitutive weak promoter (*P*_*KmGDH2*_) nor the constitutive strong promoter (*P*_*KmPDC1*_) caused significant changes in *ADE2* knockout efficiency compared to *P*_*ScTEF1*_, showing only minor reductions. In contrast, the inulin-inducible promoter (*P*_*KmINU1*_) yielded an editing efficiency of 50.70 % when plated on SD medium but only 17.60 % on SI medium (Fig. 2E and F). This disparity is likely because Cas12a, delivered as a linear DNA fragment, undergoes transient expression that may not persist long enough to allow induction by inulin.

Additionally, previous studies have demonstrated that overexpressing the recombinase Rad52 enhances homologous recombination repair efficiency in yeast, thereby improving gene editing outcomes [32,33]. Consequently, a strain overexpressing *KmRAD52* were constructed and the editing efficiency of Cas12a targeting the *ADE2*-TTA site under the control of the aforementioned promoters in this background were tested. As shown in Fig. 2E and F, the *RAD52* overexpression strain further improved the editing efficiency of Cas12a controlled by *P*_*ScTEF1*_, *P*_*KmGDH2*_, and *P*_*KmPDC1*_ by 1.52 %, 4.04 %, and 6.12 %. However, the enhancement was more significant for Cas12a controlled by *P*_*KmINU1*_, with an increase of 26.15 % on SD + Adenine plate and 34.67 % on SI + Adenine plate (Fig. 2E and F).

### 3.5 Integrated Cas12a for genome editing in *K*. *marxianus*

**Fig. 3:**
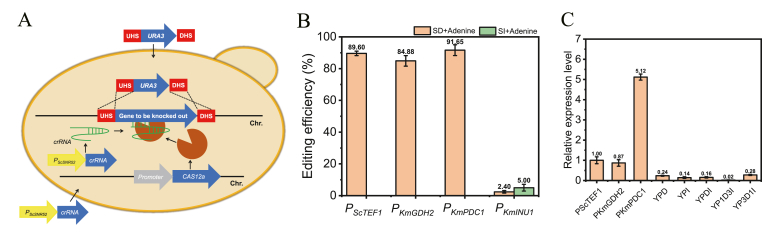
(A) Schematic representation of the integrated CRISPR/Cas12a-based genome editing system in *K. marxianus*. (B) Impact of *CAS12a* promoter strength on integrated CRISPR/Cas12a editing efficiency in *K*. *marxianus* and comparing the effects of glucose-to-inulin ratios in pre-culture to the Cas12a editing efficiency under P_*KmINU1*_ promoter on SD. (C) The expression levels of *CAS12a* controlled by *P*_*ScTEF1*_, *P*_*KmGDH2*_, *P*_*KmPDC1*_, or *P*_*KmINU1*_, in YPD medium, and by *P*_*KmINU1*_in mixed carbon sources (inulin/glucose).

While transient CRISPR/Cas12a is suitable for strains requiring fewer editing cycles (with no genomic integration or cellular toxicity), integrated CRISPR/Cas12a offers greater efficiency for multi-locus editing (Fig. 3A). Therefore, the effects of *CAS12a* expression cassettes integrated (driven by promoters *P*_*ScTEF1*_, *P*_*KmGDH2*_, *P*_*KmPDC1*_, or *P*_*KmINU1*_) into the *XYL1* locus of strain YZB101 were investigated. Using crRNA-TTA to target *ADE2*, integrated CRISPR/Cas12a achieved efficiencies comparable to the transient system: 89.60 %, 84.88 %, and 91.65 % for *P*_*ScTEF1*_, *P*_*KmGDH2*_, and *P*_*KmPDC1*_, respectively (Fig. 3B). However, the inulin-inducible *P*_*KmINU1*_ yielded consistently low efficiency on both SD and SI plates. Since constitutive *CAS12a* expression risks chronic toxicity, whereas inducible promoters minimize toxicity by restricting expression, *P*_*KmINU1*_-driven editing was optimized. Testing YPD and YPI pre-cultures followed by SD/SI plating revealed that YPD pre-culture gave <5 % editing on SD (6.20 CFU/μg) and SI (2.00 CFU/μg); YPI pre-culture produced 0.60 CFU/μg (SD) and 0.50 CFU/μg (SI) but no editing, likely due to poor growth under inulin and Cas12a-induced toxicity. Optimizing pre-culture media with blended carbon sources (YPDI, YP1D3I, and YP3D1I) significantly improved efficiency (Fig. S8). SD plates showed 22.70 % (YPDI), 25.00 % (YP1D3I), 42.50 % (YP3D1I), and SI plates showed 0 % (YPDI), 16.67 % (YP1D3I), and 25.00 % (YP3D1I). Thus, *P*_*KmINU1*_ achieves higher efficiency on SD plates, with YP3D1I pre-culture yielding optimal performance. The expression levels of *CAS12a* controlled by *P*_*ScTEF1*_, *P*_*KmGDH2*_, *P*_*KmPDC1*_, or *P*_*KmINU1*_, in YPD medium, and by *P*_*KmINU1*_ in mixed carbon sources (inulin/glucose) were quantified using RT-qPCR (Fig. 3C). *CAS12a* expression driven by *P*_*ScTEF1*_ and *P*_*KmGDH2*_ showed comparable levels, whereas *P*_*KmPDC1*_-controlled *CAS12a* exhibited 5.12-fold higher expression than *P*_*ScTEF1*_, consistent with the growth curve data. *CAS12a* expression under *P*_*KmINU1*_ control remained consistently low across conditions, aligning with its reduced editing efficiency (Fig. S8 and 3C).

### 3.6 Toxicity of integrated Cas12a in *K*. *marxianus*

To assess cellular toxicity caused by integrated Cas12a, growth curves of strains harboring *P*_*ScTEF1*_, *P*_*KmGDH2*_, *P*_*KmPDC1*_, or *P*_*KmINU1*_ promoters were analyzed in YPD and SD media (Fig. S9). Results indicated negligible toxicity from constitutive Cas12a expression in nutrient-rich YPD. However, in SD medium, Cas12a toxicity positively correlated with promoter strength, with *P*_*KmINU1*_ (inducible) showing the lowest toxicity, consistent with expectations. Similarly, when WT and *P*_*KmINU1*_-integrated strains were cultured in YPI versus SI media, no toxicity was observed in YPI, whereas *P*_*KmINU1*_ strain growth was impaired in SI. Collectively, Cas12a exhibits low, acceptable toxicity in *K. marxianus* (Fig. 3C and S9).

### 3.7 Integrated CRISPR/Cas12a for gene knock-in in *K*. *marxianus*

**Fig. 4:**
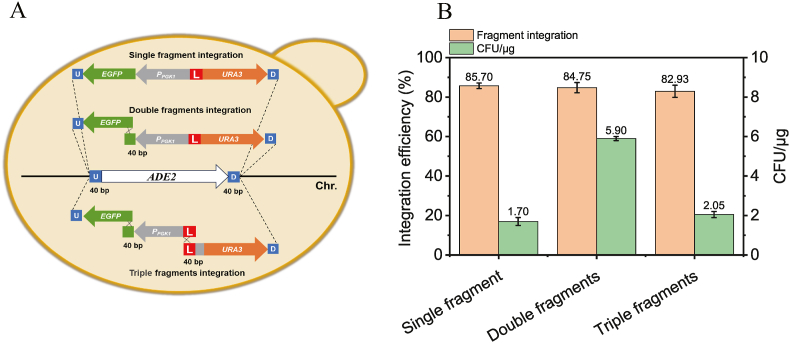
(A) Schematic representation of the single, double, and triple fragment knock-in in *K*. *marxianus* using integrated CRISPR/Cas12a. (B) Efficiency of one-step knock-in of single, double, and triple fragments mediated by integrated CRISPR/Cas12a in *K*. *marxianus*.

Beyond gene knockout, genome editing encompasses gene knock-in, which often involves complex multi-fragment in vivo recombination. To evaluate the efficiency of integrated CRISPR/Cas12a for knock-in, using an *EGFP* expression cassette, three integration strategies (single-fragment knock-in of a full *EGFP*-*URA3* expression cassette, dual-fragment knock-in with a 40-bp overlap in the *EGFP* coding region, and triple-fragment knock-in with overlaps in the *EGFP* coding region and the *EGFP*-*URA3* junction) were designed (Fig. 4A). Using crRNA-TTA targeting the *ADE2* locus and 40-bp homology/overlap arms, integration efficiency was calculated via colony color change, PCR validation, and EGFP fluorescence microscopy. Results showed surprisingly high efficiencies: 85.70 % (1.70 CFU/μg) for single-fragment, 84.75 % (5.90 CFU/μg) for dual-fragment, and 82.93 % (2.05 CFU/μg) for triple-fragment knock-in (Fig. 4B and S10). Fluorescence microscopy confirmed that all successfully integrated strains exhibited *EGFP* expression, while untransformed controls showed no fluorescence (Figs. S10 and S11), strongly suggesting minimal mutations in the overlap regions during multi-fragment assembly.

### 3.8 Large-fragment (>5 kb) knock-in efficiency in *K*. *marxianus* using integrated CRISPR/Cas12a

**Fig. 5:**
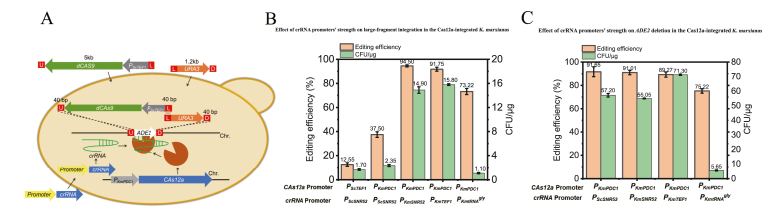
(A) Schematic of one-step *dCAS9* and *URA3* fragment knock-in procedure. (B) Impact of *CAS12a* and crRNA promoters on large-fragment knock-in efficiency in the strain with integrated *CAS12a*. (C) CrRNA promoter-dependent editing efficiency for *ADE2* knockout in the strain with integrated *CAS12a*.

To evaluate the efficacy of integrated CRISPR/Cas12a for large-fragment (>5 kb) integration, co-integration of *dCAS9* (5 kb) and *URA3* (1.2 kb) into the *ADE2* locus of *K. marxianus* were tested (Fig. 5A). Initial attempts using *P*_*ScTEF1*_-driven *CAS12a* with *P*_*ScSNR52*_-initiated crRNA yielded low efficiency (12.55 %). Suspecting stronger Cas12a expression might improve results, *P*_*KmPDC1*_-driven *CAS12a* with the same crRNA promoter were tested, yet efficiency remained unchanged at 37.50 %. Consequently, in the *P*_*KmPDC1*_-Cas12a strain, the crRNA promoter was replaced with stronger endogenous *K. marxianus* promoters (*P*_*KmSNR52*_, *P*_*KmTEF1*_, and *P*_*KmtRNA*_^*gly*^). As shown in Fig. 5B, all three native promoters significantly enhanced large-fragment integration efficiency, achieving 94.50 %, 91.75 %, and 73.22 %, respectively (Fig. 5B).

Given that the three endogenous promoters enhanced large-fragment integration efficiency, whether they could improve knockout efficiency at the *ADE2* locus were further investigated. Using the *P*_*KmPDC1*_-Cas12a-integrated strain, crRNA-TTA, and donor DNA with 60-bp homology arms, the knockout efficiencies driven by endogenous crRNA promoters (*P*_*KmSNR52*_, *P*_*KmTEF1*_, *P*_*KmtRNA*_^*gly*^) were 91.01 %, 89.27 %, and 75.22 %, respectively, similar to the *P*_*ScSNR52*_-crRNA (Fig. 5C). This suggests that residual NHEJ activity persists even in the *Δku70* background, allowing partial random integration of donor DNA into non-target genomic sites, thereby complementing the auxotrophy. Consequently, both transient and integrated Cas12a systems exhibit an efficiency ceiling of ∼90 % for fragment-based crRNA, regardless of the crRNA promoter strength (Fig. 5C).

In summary, CRISPR/Cas12a outperforms existing tools (e.g., CRISPR/Cas9) in *K. marxianus* across key metrics (Table S5). The highest gene editing efficiency achieved with transient CRISPR/Cas12a was 100 % in *Δku70* strains, surpassing transient CRISPR/Cas9's 90.9 % efficiency in this yeast [17]. Furthermore, with only 35-bp homology arms, 66.7 % efficiency was obtained using transient CRISPR/Cas12a, while CRISPR/Cas9 requires ≥40 bp, and the efficiency of 40-bp homology arms was 68.97 % [17]. Cas12a's T-rich PAM (TTN) broadens targetable genomic sites versus Cas9's NGG restriction, critical for AT-rich promoters. Its sticky-end cleavage enhances homologous recombination fidelity, reducing error-prone NHEJ. Unlike plasmid-dependent Cas9 systems, Cas12a's plasmid-free delivery minimizes metabolic burden, accelerating iterative editing. These advances establish Cas12a as the premier genome engineering toolkit for *K. marxianus*, enabling complex metabolic rewiring unattainable with prior methods.

### 3.9 Succinic acid production via *SDH* genes knockout in *K*. *marxianus*

**Fig. 6:**
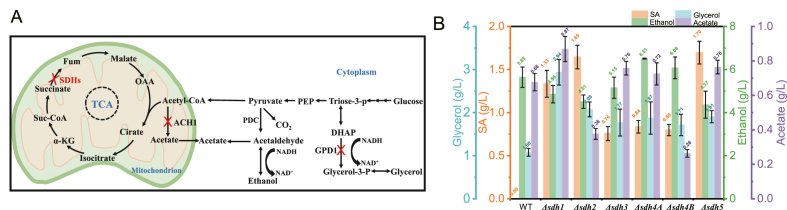
(A) Schematic diagram of succinate acid biosynthesis pathway in yeast. (B) Comparative analysis of succinate acid production capacity in *K*. *marxianus sdh1*, *sdh2*, *sdh3*, *sdh4A*, *sdh4B*, and *sdh5* single-knockout mutants grown in YPD media at 37 °C under shake-flask conditions.

Succinic acid (SA, butanedioic acid), recognized by the U.S. Department of Energy as one of the top 12 high-potential bio-based chemicals, has been extensively studied for microbial production [[34], [35], [36]]. For instance, *Yarrowia lipolytica* efficiently synthesizes succinic acid from glucose and glycerol after *SDH5* knockout (Fig. 6A) [[37], [38], [39], [40]]. However, succinic acid production in *K. marxianus* remains unreported. Through homology-based identification, six *SDH* homolog genes (*SDH1*, *SDH2*, *SDH3*, *SDH4A*, *SDH4B*, and *SDH5*) in *K. marxianus* were annotated. Using a *P*_*KmPDC1*_-Cas12a-integrated strain, single-knockout mutants for each gene were generated. Phenotypic assays at 30 °C, 37 °C, and 46 °C revealed that *SDH1* or *SDH2* deletion severely impaired growth, while *SDH3*, *SDH4A*, *SDH4B*, or *SDH5* deletion caused minimal defects. At 46 °C, all mutants exhibited significant growth impairment except *SDH4A* deletion improved strain's thermotolerance (Fig. S12A). During liquid culture, the *sdh4Δ* strain also exhibited improved growth at 46 °C compared to the control strain, although this enhancement was not statistically significant (Fig. S12B). Previous studies have demonstrated that single-subunit knockout of succinate dehydrogenase (*SDH1-SDH5*) in yeast significantly reduces thermotolerance by disrupting mitochondrial electron transport chain (ETC) function, with the core mechanism involving ROS burst and collapse of the antioxidant system: *SDH* deficiency blocks the conversion of succinate to fumarate, causing electron accumulation in the ETC and leakage to form superoxide anions (O_2_^−^). Elevated temperatures further increase ROS levels by 2 ∼ 3-fold, and this dual effect synergistically heightens yeast heat sensitivity. Concurrently, succinate accumulation inhibits prolyl hydroxylase (PHD), stabilizing HIF-1α and upregulating pro-oxidant genes (e.g., NOX4), thereby amplifying oxidative stress. High ROS levels directly damage heat shock proteins (e.g., oxidizing Hsp90 thiol groups to inactivate it) and membrane lipids (inducing mitochondrial membrane peroxidation), while also suppressing antioxidant defenses (e.g., thioredoxin system failure and reduced Mn-SOD activity). *SDH* dysfunction additionally impairs trehalose synthesis indirectly by inhibiting thiamine metabolism (THI80 downregulation leads to TPP cofactor deficiency for Tps1), depriving cells of the thermoprotective “sugar shield” [[41], [42], [43]]. We speculate that the deletion of *SDH1*, *SDH2*, *SDH3*, *SDH4B*, and *SDH5* leads to reduced thermotolerance in *K*. *marxianus*, while the enhanced thermotolerance resulting from *SDH4A* deletion is associated with ROS levels. As shown in Figs. S12C and S13, we measured changes in ROS levels in *SDH* subunit deletion strains at 30 °C and 46 °C. It was observed that at 30 °C, the ROS levels in *sdh1Δ*, *sdh2Δ*, *sdh3Δ*, *sdh4BΔ*, and *sdh5Δ* strains were 1.06, 1.14, 1.13, 1.11, and 1.10-times that of the WT, respectively. Upon temperature increase to 46 °C, the ROS levels of WT, *sdh1Δ*, *sdh2Δ*, *sdh3Δ*, *sdh4BΔ*, and *sdh5Δ* deletion strains became 1.86, 4.68, 2.27, 3.13, 3.02, and 2.78-times that of WT at 30 °C, respectively. Only the *SDH4A* deletion strain exhibited similar ROS levels with WT at 30 °C (0.98-times) and lower ROS levels with WT at 46 °C (0.57-times). Fluorescence microscopy results were consistent with the microplate reader data (Figs. S12C and S13). *Km*SDH4A and *Km*SDH4B are two homologs of the *S*. *cerevisiae* SDH4 protein. Among them, *Km*SDH4B shares higher homology (50.28 %) with *Sc*SDH4, while *Km*SDH4A exhibits only 33.68 % homology (Fig. S14). Therefore, we postulate that *Km*SDH4A is a suboptimal SDH4 homolog. Deletion of *SDH4A* allows only the highly active SDH4B to form the SDH complex in *K. marxianus*, resulting in relatively higher complex activity. This reduces ROS levels under high temperature and thereby enhances thermotolerance. Conversely, deletion of *SDH1*, *SDH2*, *SDH3*, *SDH4B*, or *SDH5* subunits results in incomplete SDH complexes with reduced activity. This leads to increased ROS levels at high temperature and consequently decreased thermotolerance.

Fermentations in YPD and YPG media quantified succinic acid, ethanol, glycerol, and acetate in WT and single *SDH* gene knockout strains (Fig. 6B and S15). *SDH* genes knockouts enabled *K. marxianus* to accumulate 0.76–1.70 g/L succinic acid from 20 g/L glucose, while no succinic acid was detected in the WT strain, alongside increased glycerol in all the *SDH* genes deleted strains. Acetate acid was increased in *sdh1Δ*, *sdh3Δ, sdh4A*, and *Δsdh5Δ*, and decreased in *sdh2Δ* and *sdh4BΔ*, while ethanol did not fluctuate much across all strains. When glycerol was the carbon source, *SDH1-5* knockouts produced only 0.58–1.43 g/L succinic acid (Fig. 6B and S15). Regardless of whether glucose or glycerol served as the carbon source, *K*. *marxianus* produced significantly lower succinic acid yields than *Y*. *lipolytica* under equivalent conditions, highlighting distinct metabolic profiles between *K. marxianus* and *S*. *cerevisiae* and *Y. lipolytica*.

**Fig. 7:**
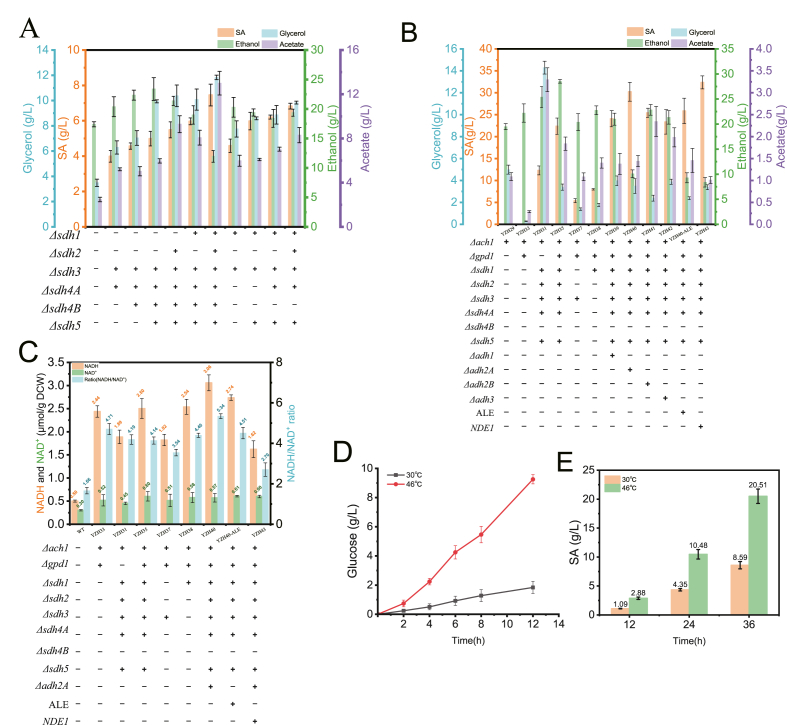
(A) The effects of combinatorial *K*. *marxianus SDHs* knockout on succinate acid and byproduct (ethanol, glycerol, and acetic acid) production. (B) Impact of *GPD1*, *ACH1*, and *ADH* genes knockout on byproduct accumulation during succinate biosynthesis in *SDH* gene*s*-deficient *K*. *marxianus*. (C) Effects of *SDH* genes, *GPD1*, *ACH1*, and *ADH* genes knockout, *NDE1* overexpression, and ALE on intracellular NADH and NAD^+^ contents and NADH/NAD^+^ ratio. (D) Cellulose hydrolysis by cellulase at 30 °C and 46 °C. (E) Simultaneous saccharification and fermentation of cellulose by YZH43 for SA synthesis at 30 °C and 46 °C.

To boost succinic acid synthesis, combinatorial *SDH* genes knockout strains were constructed (*sdh3Δsdh4Δ*, *sdh3Δsdh4AΔsdh4BΔ*, *sdh3Δsdh4AΔsdh4BΔsdh5Δ*, *sdh3Δsdh4AΔsdh4BΔsdh5Δsdh2Δ*, *sdh3Δsdh4AΔsdh4BΔsdh5Δsdh1Δ*, *sdh3Δsdh4AΔsdh4BΔsdh5Δsdh1Δsdh2Δ*, *sdh1Δsdh3Δsdh5Δ*, *sdh1Δsdh3Δsdh5Δsdh4AΔ*, and *sdh1Δsdh3Δsdh5Δsdh4AΔsdh2Δ)*. Fermentation in YP10D medium revealed that succinic acid titers increased with the number of *SDH* genes knockouts, with strains retaining only *SDH1*, only *SDH2*, only *SDH4B*, or no *SDH* genes producing 5.49 g/L, 5.97 g/L, 6.83 g/L, and 7.46 g/L, respectively. However, byproduct accumulation also rose significantly, glycerol reached 10.37, 10.08, 9.84, and 11.83 g/L, while acetate reached 9.29, 8.07, 8.29, and 12.99 g/L in the respective strains (Fig. 7A).

### 3.10 Optimizing succinic acid production via *ACH1* and *GPD1* co-knockout

While *SDH* genes knockout in *S*. *cerevisiae* also elevates acetate production, the increase is less pronounced than in *K. marxianus*. To mitigate byproducts, we targeted *ACH1* (acetyl-CoA hydrolase) and *GPD1* (glycerol-3-phosphate dehydrogenase), the latter previously shown to reduce glycerol accumulation [44,45]. However, attempts to knockout *ACH1* or *GPD1* in the full *SDH* genes knockout strain (*ΔSDH1-5*) failed, likely due to severe metabolic fragility. As an alternative, strains *ach1Δ, ach1Δgpd1Δ*, *sdh1Δsdh3Δsdh5Δsdh4AΔsdh2Δach1Δ*, *sdh1Δsdh3Δsdh5Δsdh4AΔsdh2Δach1Δgpd1Δ*, *ach1Δgpd1Δsdh3Δ*, and *ach1Δgpd1Δsdh1Δ* were constructed. Fermentation in YP10D revealed that *ACH1* knockout reduced acetate by 58.33 % versus WT, while *GPD1* knockout decreased glycerol by 92.95 %. Among these, the *sdh1Δsdh3Δsdh5Δsdh4AΔsdh2Δach1Δgpd1Δ* strain achieved the highest succinic acid titer (22.39 g/L), with byproducts at 28.52 g/L ethanol, 3.39 g/L glycerol, and 1.84 g/L acetate (Fig. 7B).

### 3.11 Optimizing succinic acid production via *ADH* genes knockout

Although the SA yield in strain YZH35 was significantly improved, substantial accumulation of ethanol as a byproduct persisted. To reduce ethanol synthesis, we sequentially deleted four ethanol dehydrogenase genes (*ADH1*, *ADH2A*, *ADH2B*, and *ADH3*) in YZH35. As shown in Fig. 7B, strains deletion of *ADH1*, *ADH2A*, *ADH2B*, *ADH3* produced 24.08 g/L, 30.25 g/L, 25.44 g/L, and 23.35 g/L SA, among them, strain deleted *ADH2A* exhibits the highest SA titer (Fig. 7B). However, the combined deletion of *SDH* genes, *GPD1*, *ACH1*, and *ADH2A* caused a significant growth defect compared to the WT (Fig. S16). This impairment stems from substantial intracellular NADH accumulation resulting from the deletion of *GPD1*, *ACH1*, and *ADH2A* (Fig. 6A), which elevated the NADH/NAD^+^ ratio from 1.66 in WT to 4.71, 4.19, 4.14, 3.54, and 4.40 in YZH33, YZH31, YZH35, YZH37, and YZH38 (Fig. 7C). The deletion of *ADH2A* further increased the NADH/NAD^+^ ratio to 5.34 in YZH40. Consequently, we hypothesized that overexpressing the external mitochondrial NADH dehydrogenase *NDE1* could restore growth by lowering the NADH/NAD^+^ ratio [4]. As anticipated, *NDE1* overexpression reduced the ratio to 2.70 (Fig. 7C). Additionally, adaptive laboratory evolution (ALE) was employed to restore growth in the engineered strain. ALE decreased the NADH/NAD^+^ ratio to 4.51. Fig. S16 demonstrates that both *NDE1* overexpression and ALE successfully recovered growth. *NDE1* overexpression increased SA titer to 32.38 g/L while reducing ethanol content to 8.38 g/L, while the YZH40-ALE strain exhibited reduced SA production capacity (25.87 g/L). This aligns with literature reports, as the selection pressure (survival and growth under high-glucose stress) did not match the engineering objective (SA overproduction) [46].

We also measured the accumulation of intracellular intermediates, fumarate and malate. As shown in Fig. S17, deletion of *ADH2A* caused malate accumulation to reach 6.85 g/L, while expression of *NDE1* reduced it to 3.52 g/L. Fumarate was detectable only in the *ADH2A* knockout strain and was not detected in other strains. *ADH2A* deletion diverts acetyl-CoA into the glyoxylate shunt, overproducing malate, while combined NADH overload drives excessive oxaloacetate reduction to malate by MDH. Fumarate buildup further inhibits its own conversion, amplifying retention. Anaplerotic pyruvate carboxylation also contributes malate. Thus, blocked catabolism and redirected carbon flux under redox stress jointly increase both metabolites.

### 3.12 SA production through SSF using cellulose as substrate

Cellulose must be hydrolyzed into glucose to be utilized by yeast. Therefore, bioconversion using cellulosic biomass involves two distinct processes—hydrolysis and fermentation—which prolongs the overall fermentation time. The optimum temperature for cellulolytic enzymes is typically above 45 °C, whereas conventional yeast such as *S*. *cerevisiae* ferments optimally at 30 °C. This temperature mismatch restricts the implementation of simultaneous saccharification and fermentation (SSF). Employing thermotolerant yeast like *K*. *marxianus* enables high-temperature SSF. This approach significantly reduces cellulase costs, as demonstrated in Fig. 7D: the cellulose hydrolysis rate at 46 °C is 5.03-fold higher than at 30 °C (Fig. 7D). Using strain YZH43, SA titers of 8.59 and 20.51 g/L were achieved from 100 g/L cellulose at 30 °C and 46 °C, with productivity of 0.24 and 0.57 g/L/h, respectively, fully demonstrating the advantages of high-temperature fermentation (Fig. 7E). Furthermore, high-temperature fermentation effectively mitigates contamination risks and reduces cooling water requirements. To our knowledge, this represents the first engineered *K. marxianus* strain for SA production, leveraging its rapid growth, broad substrate utilization, and thermotolerance to offer a promising alternative for green biosynthesis.

## 4 Conclusions

This study pioneers a high-efficiency CRISPR/Cas12a toolbox for *K*. *marxianus*, overcoming genetic limitations through transient and integrated systems achieving 50 %–100 % knockout efficiency. Even with homology arms as short as 35 bp, the knockout efficiency remained 66.67 %. Chromosomal integration of Cas12a enabled single-to-triple fragment knock-ins efficiency of 82.93–85.70 % and 94.50 % for large fragment (>5 kb) integrations. Applied to metabolic redesign, combinatorial *SDH* genes knockout redirected carbon flux toward succinic acid SA and subsequent *GPD1*/*ACH1/ADH2A* co-knockout in a *Δsdh1,3,5,4A,2* strain with *NDE1* overexpression yielded 32.38 g/L SA at 37 °C, while reducing ethanol, acetate, and glycerol by 60.79 %, 89.24 %, and 67.5 %. This breakthrough positions *K. marxianus* as a versatile thermotolerant chassis for sustainable bioproduction.

## CRediT authorship contribution statement

**Hao Zha:** Investigation. **Yanjie Li:** Investigation. **Zhongmei Hu:** Investigation. **Jiacheng Li:** Investigation. **Yujie Xie:** Investigation. **Mingtao Zhao:** Writing – review & editing, Supervision, Software. **Lili Ren:** Writing – review & editing, Writing – original draft, Supervision. **Biao Zhang:** Writing – review & editing, Writing – original draft, Supervision, Software, Funding acquisition.

## Declaration of competing interest

The authors declare that they have no known competing financial interests or personal relationships that could have appeared to influence the work reported in this paper.

## References

[1] Cernak P., Estrela R., Poddar S., Skerker J.M., Cheng Y.F., Carlson A.K., Chen B., Glynn V.M., Furlan M., Ryan O.W. (2018). Engineering *Kluyveromyces marxianus* as a robust synthetic biology platform host. mBio.

[2] Ren L.L., Zha H., Zhang Q., Xie Y.J., Li J.C., Hu Z.M., Tao X.R., Xu D.Y., Li F., Zhang B. (2024). Altered sterol composition mediates multiple tolerance of *Kluyveromyces marxianus* for xylitol production. Microb Cell Fact.

[3] Baptista M., Domingues L. (2022). *Kluyveromyces marxianus* as a microbial cell factory for lignocellulosic biomass valorisation. Biotechnol Adv.

[4] Zhang B., Ren L., Zeng S., Zhang S., Xu D., Zeng X., Li F. (2020). Functional analysis of *PGI1* and *ZWF1* in thermotolerant yeast *Kluyveromyces marxianus*. Appl Microbiol Biotechnol.

[5] Zhang B., Ren L., Zhao Z., Zhang S., Xu D., Zeng X., Li F. (2021). High temperature xylitol production through simultaneous co-utilization of glucose and xylose by engineered *Kluyveromyces marxianus*. Biochem Eng J.

[6] Nurcholis M., Lertwattanasakul N., Rodrussamee N., Kosaka T., Murata M., Yamada M. (2019). Integration of comprehensive data and biotechnological tools for industrial applications of *Kluyveromyces marxianus*. Appl Microbiol Biotechnol.

[7] Rajkumar A.S., Varela J.A., Juergens H., Daran J.G., Morrissey J.P. (2019). Biological parts for *Kluyveromyces marxianus* synthetic biology. Front Bioeng Biotechnol.

[8] Zhang B., Ren L., Wang Y., Xu D., Zhang S., Wang H.N., Wang H., Zeng X., Xin B., Li F. (2020). Glycerol production through *TPI1* defective *Kluyveromyces marxianus* at high temperature with glucose, fructose, and xylose as feedstock. Biochem Eng J.

[9] Rajkumar A.S., Morrissey J.P. (2022). Protocols for marker-free gene knock-out and knock-down in *Kluyveromyces marxianus* using CRISPR/Cas9. FEMS Yeast Res.

[10] Ploessl D., Zhao Y., Cao M., Ghosh S., Lopez C., Sayadi M., Chudalayandi S., Severin A., Huang L., Gustafson M., Shao Z. (2021). A repackaged CRISPR platform increases homology-directed repair for yeast engineering. Nat Chem Biol.

[11] Lee M.H., Lin J.J., Lin Y.J., Chang J.J., Ke H.M., Fan W.L., Wang T.Y., Li W.H. (2018). Genome-wide prediction of CRISPR/Cas9 targets in *Kluyveromyces marxianus* and its application to obtain a stable haploid strain. Sci Rep.

[12] Juergens H., Varela J.A., Gorter de Vries A.R., Perli T., Gast V.J.M., Gyurchev N.Y., Rajkumar A.S., Mans R., Pronk J.T., Morrissey J.P., Daran J.G. (2018). Genome editing in *Kluyveromyces* and *Ogataea* yeasts using a broad-host-range Cas9/gRNA co-expression plasmid. FEMS Yeast Res.

[13] Nambu-Nishida Y., Nishida K., Hasunuma T., Kondo A. (2017). Development of a comprehensive set of tools for genome engineering in a cold- and thermo-tolerant *Kluyveromyces marxianus* yeast strain. Sci Rep.

[14] Lobs A.K., Engel R., Schwartz C., Flores A., Wheeldon I. (2017). CRISPR-Cas9-enabled genetic disruptions for understanding ethanol and ethyl acetate biosynthesis in *Kluyveromyces marxianus*. Biotechnol Biofuels.

[15] Zhou H.Y., Tian T., Liu J.T., Lu H., Yu Y., Wang Y.M. (2024). Efficient and markerless gene integration with SlugCas9-HF in *Kluyveromyces marxianus*. Commun Biol.

[16] Wang W.L., Wang X.K., Tan Y.D., Zhao S., Zhao L.Q., Zhu Z.W. (2024). CRISPR-Cas9 mediated genome editing of *Kluyveromyces marxianus* for iterative, multiplexed gene disruption and pathway integration. Biotechnol Bioeng.

[17] Ren L., Liu Y., Xia Y., Huang Y., Liu Y., Wang Y., Li P., Chang K., Xu D., Li F., Zhang B. (2022). Improving glycerol utilization during high-temperature xylitol production with *Kluyveromyces marxianus* using a transient clustered regularly interspaced short palindromic repeats (CRISPR)/CRISPR-associated protein 9 system. Bioresour Technol.

[18] Yao R.L., Liu D., Jia X., Zheng Y., Liu W., Xiao Y. (2018). CRISPR-Cas9/Cas12a biotechnology and application in bacteria. Synth Syst Biotechnol.

[19] Joseph R.C., Sandoval N.R. (2023). Single and multiplexed gene repression in solventogenic *Clostridium* via Cas12a-based CRISPR interference. Synth Syst Biotechnol.

[20] Bandyopadhyay A., Kancharla N., Javalkote V.S., Dasgupta S., Brutnell T.P. (2020). CRISPR-Cas12a (Cpf1): a versatile tool in the plant genome editing tool box for agricultural advancement. Front Plant Sci.

[21] Chen X.M., Li C.Y., Qiu X., Chen M., Xu Y.P., Li S.Y., Li Q., Wang L. (2025). CRISPR/Cas9-based iterative multi-copy integration for improved metabolite yields in *Saccharomyces cerevisiae*. Synth Syst Biotechnol.

[22] Meliawati M., Schilling C., Schmid J. (2021). Recent advances of Cas12a applications in bacteria. Appl Microbiol Biotechnol.

[23] Bai W.X., Huang M.L., Li C., Li J. (2023). The biological principles and advanced applications of DSB repair in CRISPR-mediated yeast genome editing. Synth Syst Biotechnol.

[24] Zhang X.Y., Gu S.J., Zheng X.Y., Peng S.Q., Li Y.R., Lin Y., Liang S.L. (2021). A novel and efficient genome editing tool assisted by CRISPR-Cas12a/Cpf1 for *Pichia pastoris*. ACS Synth Biol.

[25] Ciurkot K., Vonk B., Gorochowski T.E., Roubos J.A., Verwaal R. (2019). CRISPR/Cas12a multiplex genome editing of *Saccharomyces cerevisiae* and the creation of yeast pixel art. J Vis Exp.

[26] Ramesh A., Lee S., Wheeldon I. (2024). Genome editing, transcriptional regulation, and forward genetic screening using CRISPR-Cas12a systems in *Yarrowia lipolytica*. Methods Mol Biol.

[27] Hong J., Ye X.H., Wang Y.R., Zhang Y.H.P. (2008). Bioseparation of recombinant cellulose-bindning module-proteins by affinity adsorption on an ultra-high-capacity cellulosic adsorbent. Anal Chim Acta.

[28] Zhang B., Zhu Y.L., Zhang J., Wang D.M., Sun L.H., Hong J. (2017). Engineered *Kluyveromyces marxianus* for pyruvate production at elevated temperature with simultaneous consumption of xylose and glucose. Bioresour Technol.

[29] Liao L.T., Shen X.R., Shen Z.Y., Du G.C., Li J.H., Zhang G.Q. (2024). CRISPR/Cas9-Based genome editing for protein expression and secretion in *Kluyveromyces lactis*. ACS Synth Biol.

[30] Zetsche B., Gootenberg J.S., Abudayyeh O.O., Slaymaker I.M., Makarova K.S., Essletzbichler P., Volz S.E., Joung J., van der Oost J., Regev A. (2015). Cpf1 is a single RNA-guided endonuclease of a class 2 CRISPR-Cas system. Cell.

[31] Stojkovic L., Gligorovski V., Geramimanesh M., Labagnara M., Rahi S.J. (2025). Automated plasmid design for marker-free genome editing in budding yeast. G3.

[32] Cai P., Duan X.P., Wu X.Y., Gao L.H., Ye M., Zhou Y.J. (2021). Recombination machinery engineering facilitates metabolic engineering of the industrial yeast *Pichia pastoris*. Nucleic Acids Res.

[33] Wang X., Li Y., Jin Z.H., Liu X.J., Gao X., Guo S.Y., Yu T. (2023). A novel CRISPR/Cas9 system with high genomic editing efficiency and recyclable auxotrophic selective marker for multiple-step metabolic rewriting in *Pichia pastoris*. Synth Syst Biotechnol.

[34] Jiang Z., Cui Z., Zhu Z., Liu Y., Tang Y.J., Hou J., Qi Q. (2021). Engineering of *Yarrowia lipolytica* transporters for high-efficient production of biobased succinic acid from glucose. Biotechnol Biofuels.

[35] Yan D., Wang C., Zhou J., Liu Y., Yang M., Xing J. (2014). Construction of reductive pathway in *Saccharomyces cerevisiae* for effective succinic acid fermentation at low pH value. Bioresour Technol.

[36] Raab A.M., Gebhardt G., Bolotina N., Weuster-Botz D., Lang C. (2010). Metabolic engineering of *Saccharomyces cerevisiae* for the biotechnological production of succinic acid. Metab Eng.

[37] Li P., Fu X., Li S., Zhang L. (2018). Engineering TATA-binding protein Spt15 to improve ethanol tolerance and production in *Kluyveromyces marxianus*. Biotechnol Biofuels.

[38] Yang X., Wang H., Li C., Lin C.S.K. (2017). Restoring of glucose metabolism of engineered *Yarrowia lipolytica* for succinic acid production via a simple and efficient adaptive evolution strategy. J Agric Food Chem.

[39] Cui Z., Gao C., Li J., Hou J., Lin C.S.K., Qi Q. (2017). Engineering of unconventional yeast *Yarrowia lipolytica* for efficient succinic acid production from glycerol at low pH. Metab Eng.

[40] Gao C.J., Yang X.F., Wang H.M., Rivero C.P., Li C., Cui Z.Y., Qi Q.S., Lin C.S.K. (2016). Robust succinic acid production from crude glycerol using engineered *Yarrowia lipolytica*. Biotechnol Biofuels.

[41] Herrero E., Ros J., Bellí G., Cabiscol E. (2008). Redox control and oxidative stress in yeast cells. Biochim Biophys Acta.

[42] Zhang M., Shi J., Jiang L. (2015). Modulation of mitochondrial membrane integrity and ROS formation by high temperature in *Saccharomyces cerevisiae*. Electron J Biotechnol.

[43] Smith E.H., Janknecht R., Maher L.J. (2007). Succinate inhibition of α-ketoglutarate-dependent enzymes in a yeast model of paraganglioma. Hum Mol Genet.

[44] Babaei M., Kildegaard K.R., Niaei A., Hosseini M., Ebrahimi S., Sudarsan S., Angelidaki I., Borodina I. (2019). Engineering *oleaginous* yeast as the host for fermentative succinic acid production from glucose. Front Bioeng Biotechnol.

[45] Yu Q.L., Cui Z.Y., Zheng Y.Q., Huo H.L., Meng L.L., Xu J.J., Gao C.J. (2018). Exploring succinic acid production by engineered *Yarrowia lipolytica* strains using glucose at low pH. Biochem Eng J.

[46] Sun W.C., Tang Y.L., Tian Y.H., Liu Z.K., Xiong W.W., Zhang H.S., Chen L., Wu H.Y., Ma Q., Xie X.X. (2025). Robust production of N-acetyl-glucosamine in engineered *Escherichia coli* from glycerol-glucose mixture. Synth Syst Biotechnol.

