# Supplementary material for: Development of a CRISPR/Cas12a genome editing toolbox in Kluyveromyces marxianus and its application in succinic acid biosynthesis
Source: Synth Syst Biotechnol. 2025 Sep 16;11:193–204. doi: 10.1016/j.synbio.2025.09.015 (PMC12510058; doi:10.1016/j.synbio.2025.09.015)
Supplement: Multimedia component 1 [file mmc1.doc]

Development of a CRISPR/Cas12a genome editing toolbox in *Kluyveromyces marxianus* and its application in succinic acid biosynthesis

Hao Zha^1^, Yanjie Li^1^, Zhongmei Hu, Jiacheng Li, Yujie Xie, Mingtao Zhao, Lili Ren^*^, and Biao Zhang^**^

Anhui Province Key Laboratory of Pollutant Sensitive Materials and Environmental Remediation, School of Life Sciences, Huaibei Normal University, Huaibei, Anhui 235000, P. R. China

^*^Corresponding author:

Lili Ren, renlili@chnu.edu.cn, Phone: +86 561-3802235, Fax: +86 561-3802235;

^**^Corresponding author:

Biao Zhang, [zhangbiao@chnu.edu.cn](mailto:zhangbiao@chnu.edu.cn), Phone: +86 561-3802235, Fax: +86 561-3802235.

^1^These authors contributed equally to this work.

**Construction process of an engineered *K. marxianus* strain for succinic acid production**

The strain construction involved sequential gene knockouts starting from YZB599: deletion of *SDH5* yielding YZH01, *SDH1* yielding YZH02, *SDH2* yielding YZH03, *SDH4A* yielding YZH04, *SDH4B* yielding YZH05, and *SDH3* yielding YZH06; YZH06 was then subjected to *ScURA3* deletion to generate YZH07, followed by *SDH4* knockout producing YZH08; YZH09 underwent *SDH4B* deletion resulting in YZH10; YZH11 had *SDH5* knocked out to create YZH12, which was further modified by *ScURA3* deletion to yield YZH13; YZH02 was derived from *ScURA3* knockout to form YZH14, followed by *SDH2* deletion generating YZH15; YZH13 was engineered with *SDH2* deletion to produce YZH16 and *SDH1* deletion to create YZH17; YZH17 then had *ScURA3* deleted yielding YZH18, followed by *SDH2* knockout producing YZH19; YZH15 underwent *ScURA3* deletion to generate YZH20, which was then modified by *SDH3* knockout yielding YZH21; YZH21 had *ScURA3* deleted to form YZH22, followed by *SDH5* knockout creating YZH23; YZH23 was subjected to *ScURA3* deletion yielding YZH24, followed by *SDH4A* knockout producing YZH25; YZH25 had *ScURA3* deleted to generate YZH26, followed by *SDH2* knockout yielding YZH27; YZH27 underwent *ScURA3* deletion forming YZH28; YZB599 was modified by *ACH1* deletion to create YZH29, followed by *ScURA3* deletion yielding YZH30; YZH28 underwent *ACH1* deletion to generate YZH31, followed by *ScURA3* deletion producing YZH32; YZH30 was engineered with *GPD1* deletion yielding YZH33, followed by *ScURA3* deletion forming YZH34; YZH32 had *GPD1* knocked out to create YZH35, followed by *ScURA3* deletion yielding YZH36; YZH34 was modified by *SDH3* deletion producing YZH37 and *SDH4* deletion yielding YZH38; Finally, YZH36 was used to generate derivative strains: deletion of *ADH1* yielding YZH39, *ADH2A* yielding YZH40, *ADH2B* yielding YZH41, *ADH3* yielding YZH42, and integration of the *NDE1* expression cassette at the *ADH2A* locus producing YZH43.


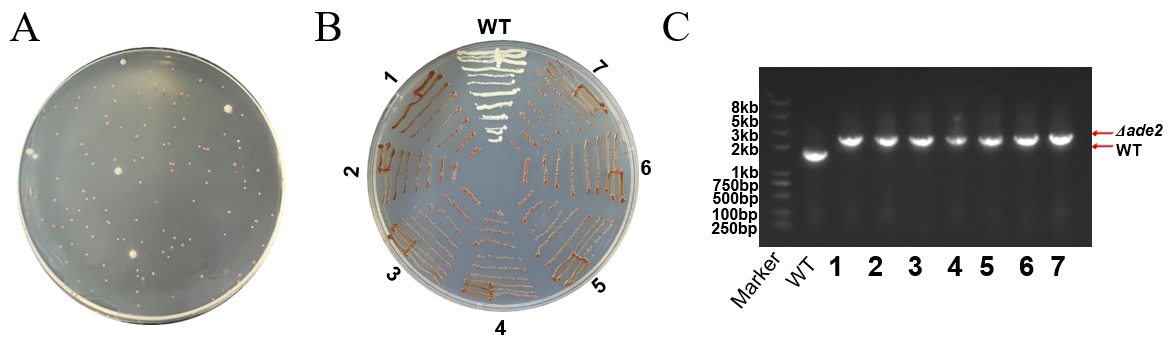


**Fig. S1 The transformation plates(A) and streak verification plates(B) for *ADE2* gene deletion in *K. marxianus*****,** **further verification was performed through streak purification and PCR identification of extracted genomic DNA(C).**


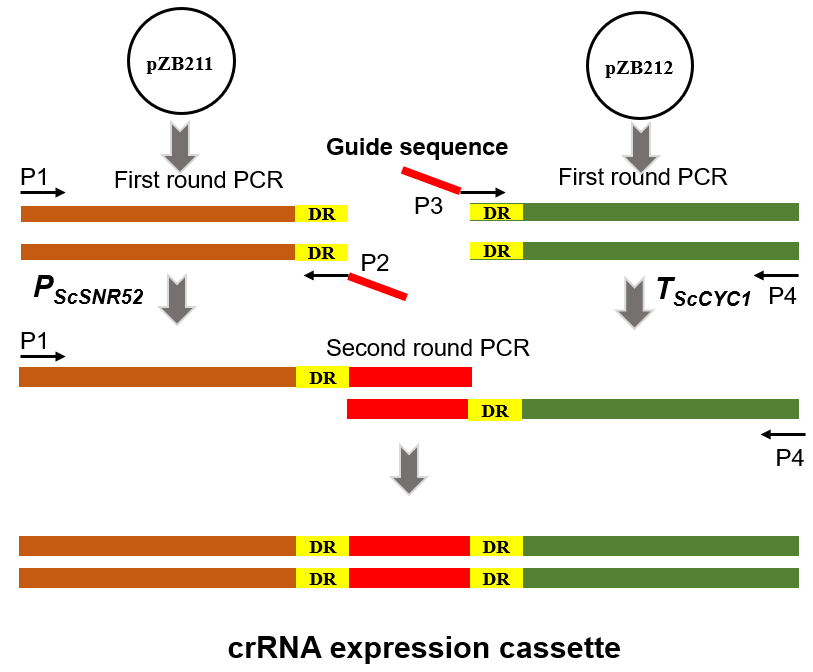


**Fig. S2 Schematic diagram of constructing crRNA using fusion PCR**


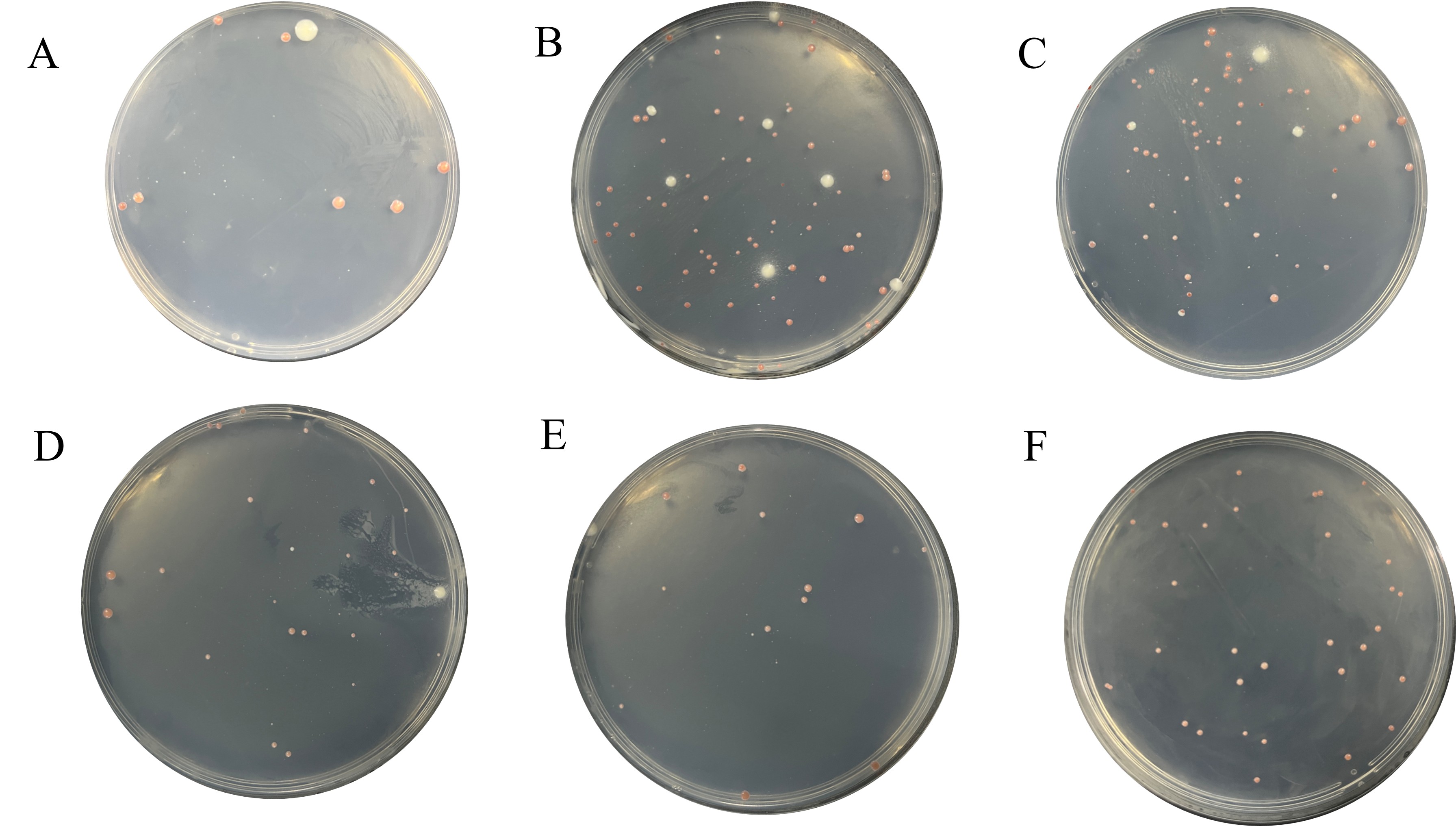


**Fig. S3 Gene editingefficiency of 40 or 50 μg of DNA fragments of Cas12a expression cassette, crRNA transcription cassette, and donor DNA with mass ratio of 1:1:2 (A), 1:2:1 (B), 2:1:1 (C), 2:2:1 (D), 2:1:2 (E), and 1:2:2 (F) were transformed into YZB101**


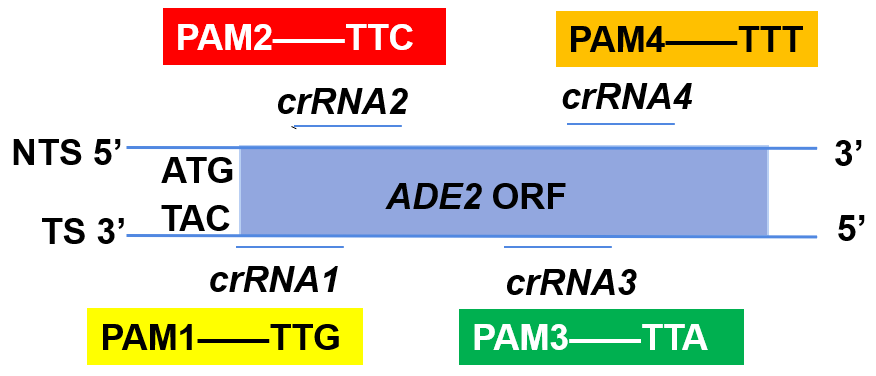


**Fig. S4 Schematic diagram of PAM sites location within the *ADE2***


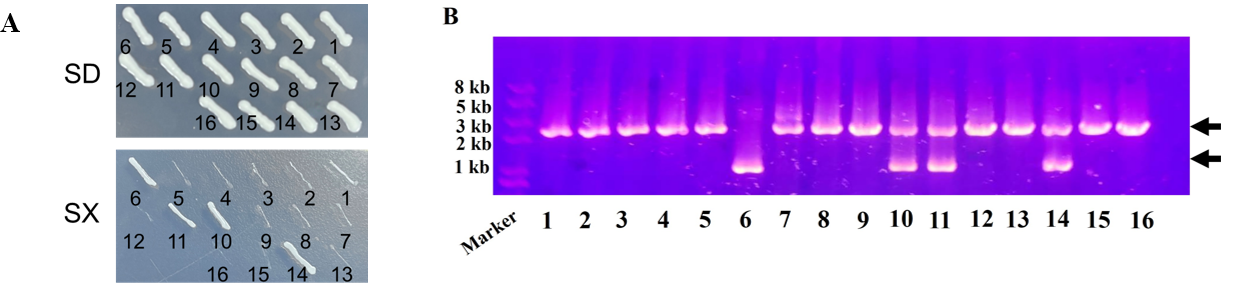


**Fig. S5 Verification of *XYL2* gene knockout by plate culture (A) and genomic PCR detection(B), samples 1-8: *XYL2*-2 locus, samples 9-16: *XYL2*-1 locus, positive band: 2.2 kb, negative band:1 kb**


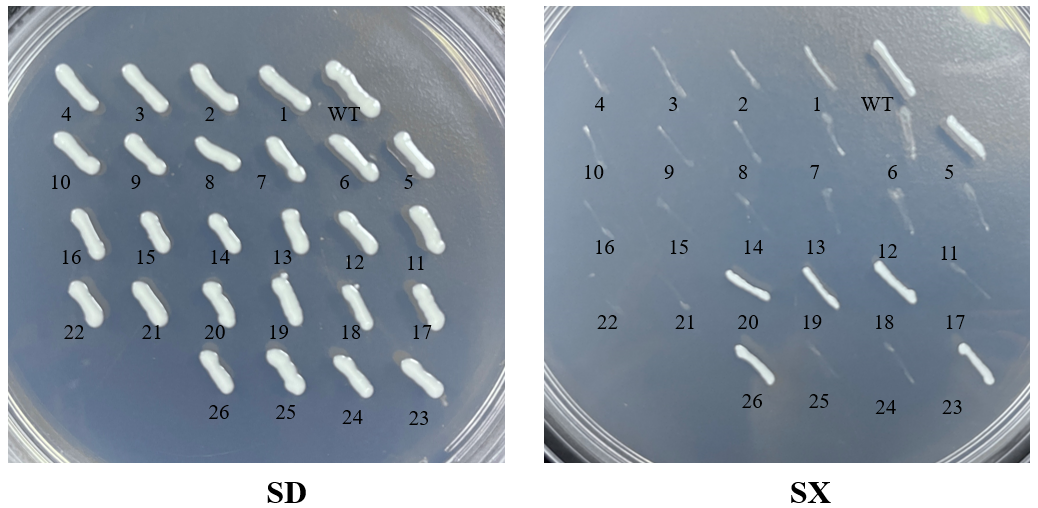


**Fig. S6 Verification of *XYL1* gene knockout by plate culture**


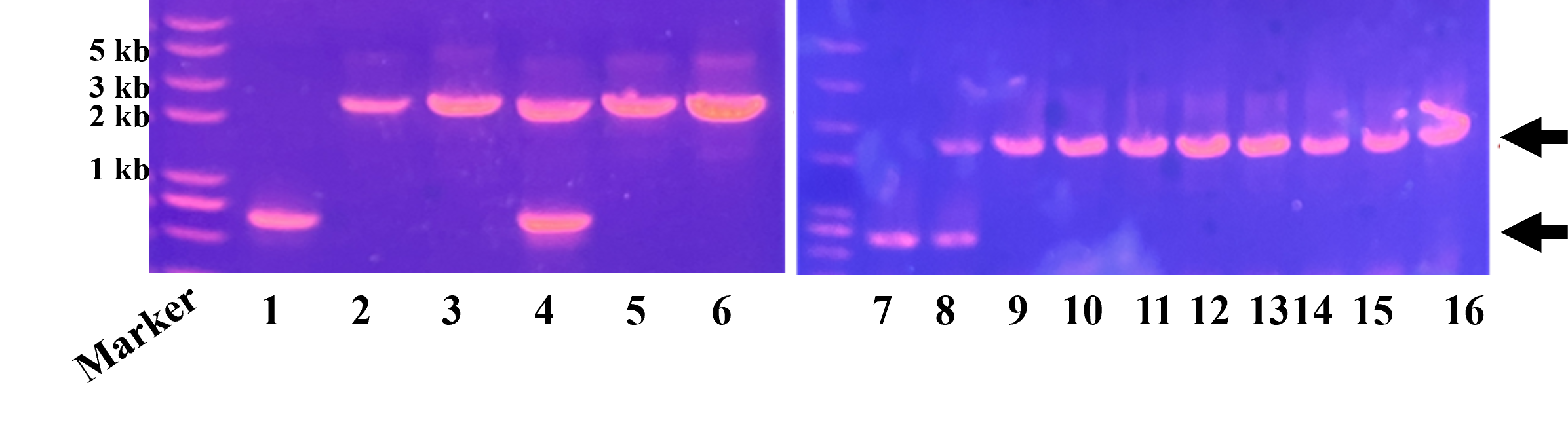


**Fig. S7 PCR verification of *TRP1* gene knockout，positive band: 2.2 kb, negative band: 600 bp**


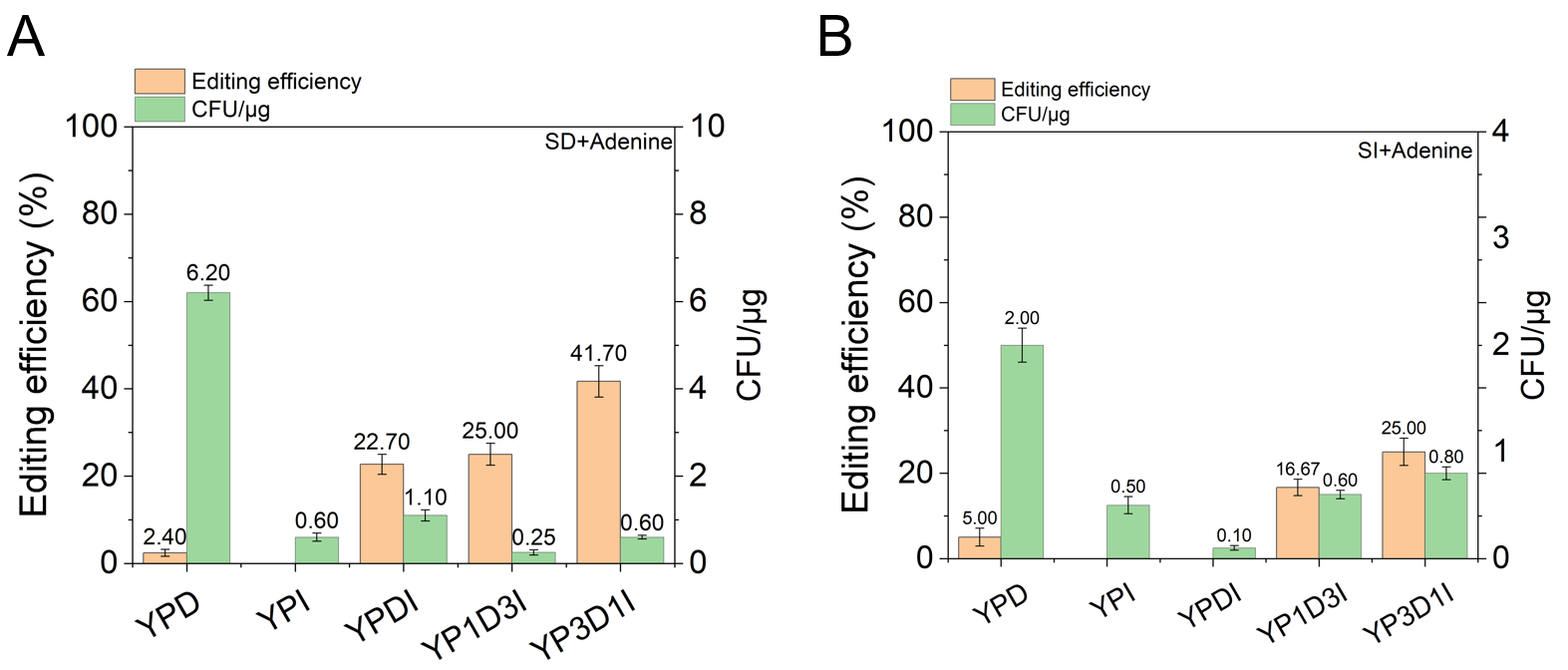


**Fig. S8 The strain integrated with the** ***P_KmINU1_*-CAS12a fragment was pre-cultured** **in mixed carbon sources (inulin/glucose), then plated on either SD(A) or SI(B) plates to test whether different pre-culture conditions and plating methods affect the editing efficiency.**


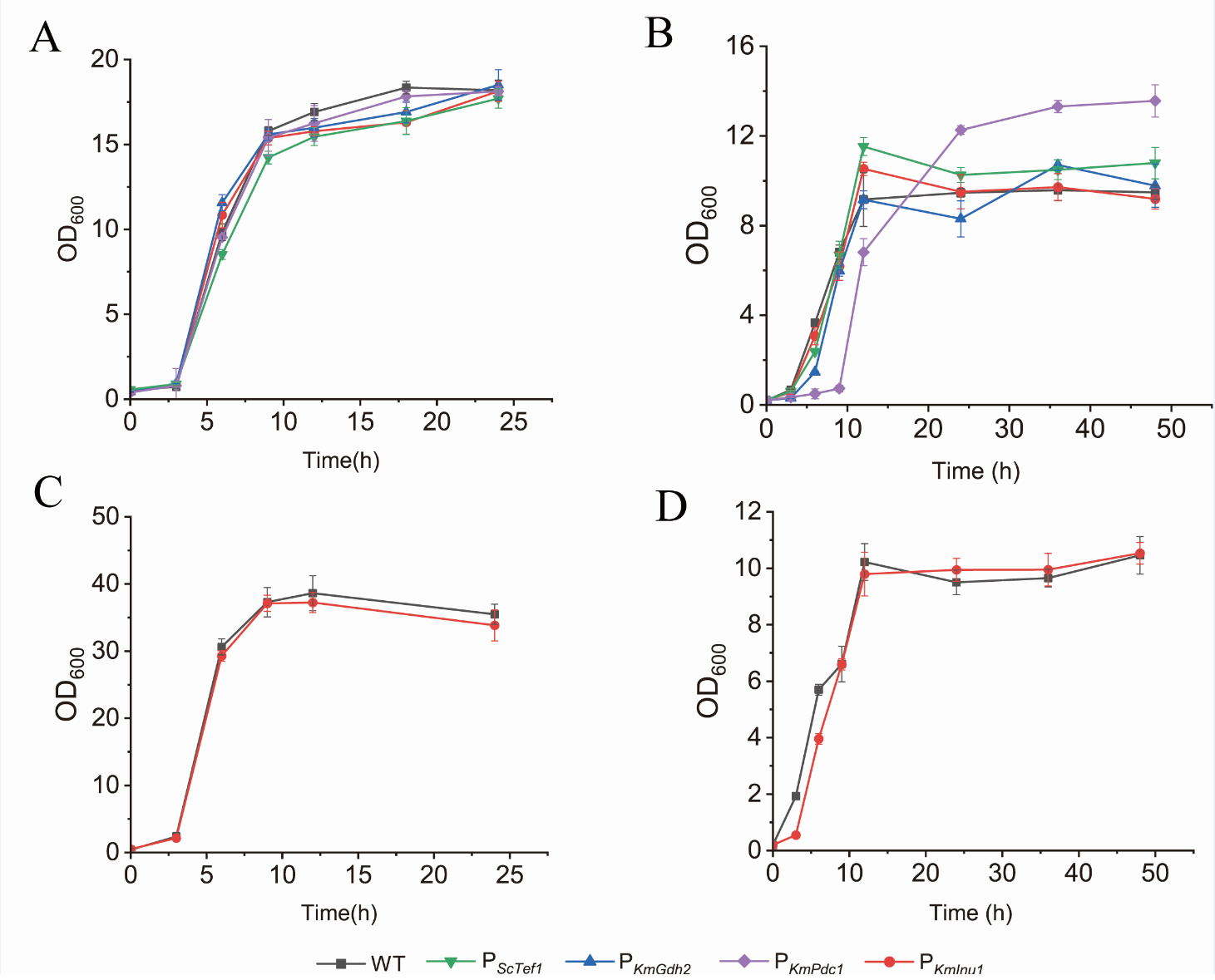


**Fig. S9** **To assess cellular toxicity caused by integrated Cas12a, growth curves of strains harboring*****P_ScTEF1_*, *P_KmGDH2_*, *P_KmPDC1_*, or *P_KmINU1_* promoters were analyzed in YPD and SD media.**


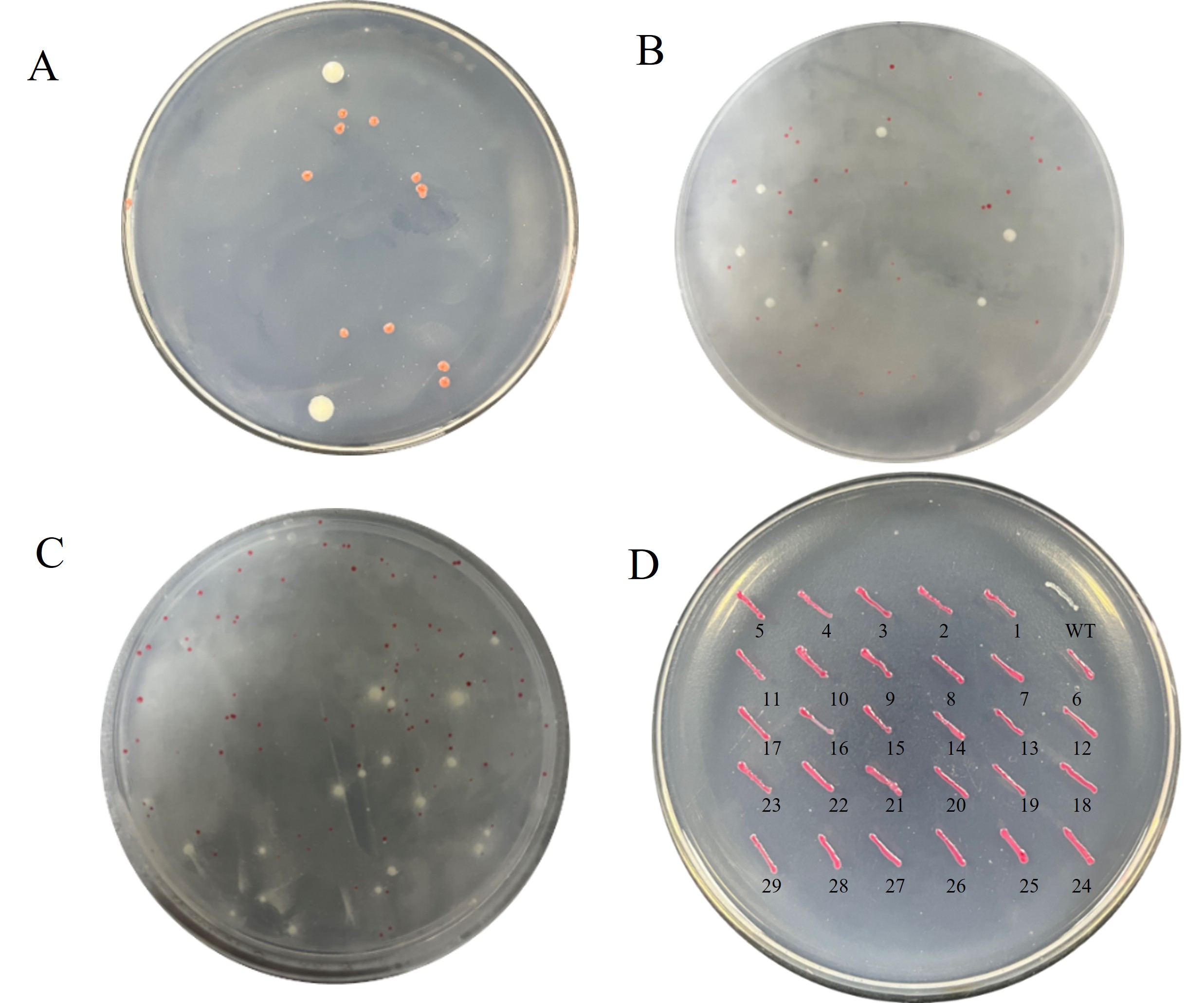


**Fig. S10 Transformation plates for single(A), double(B), and triple(C)-fragment one-step integration, and streaking results of selected colonies (D)**.


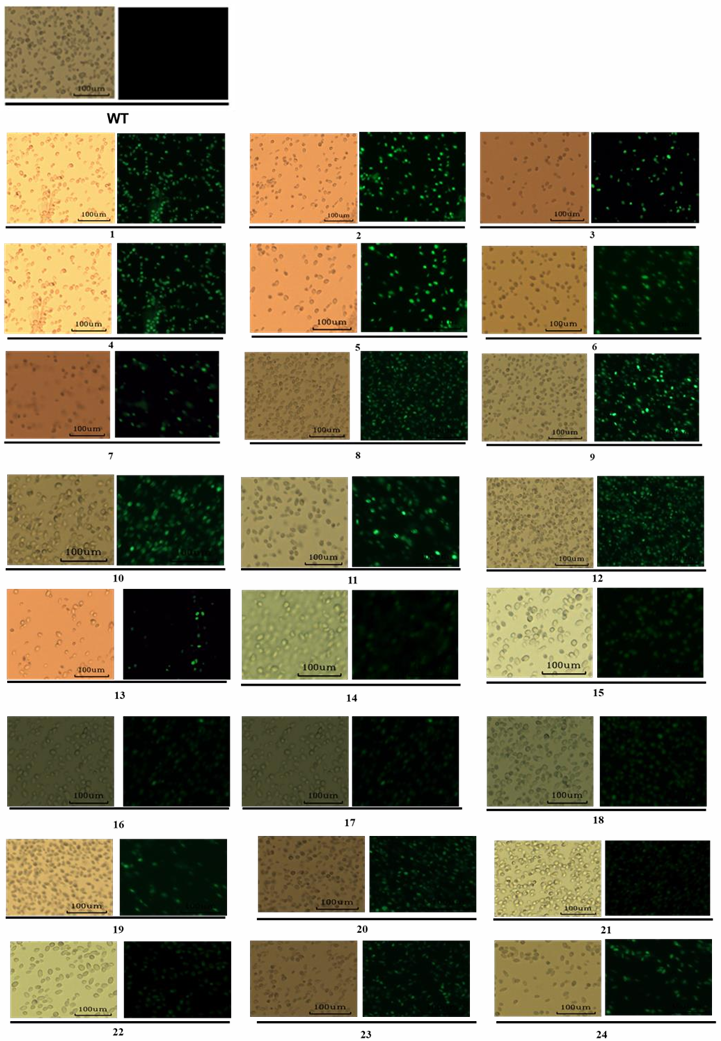


**Fig. S11 Fluorescence microscopic observation of *EGFP*-transformed red colonies**


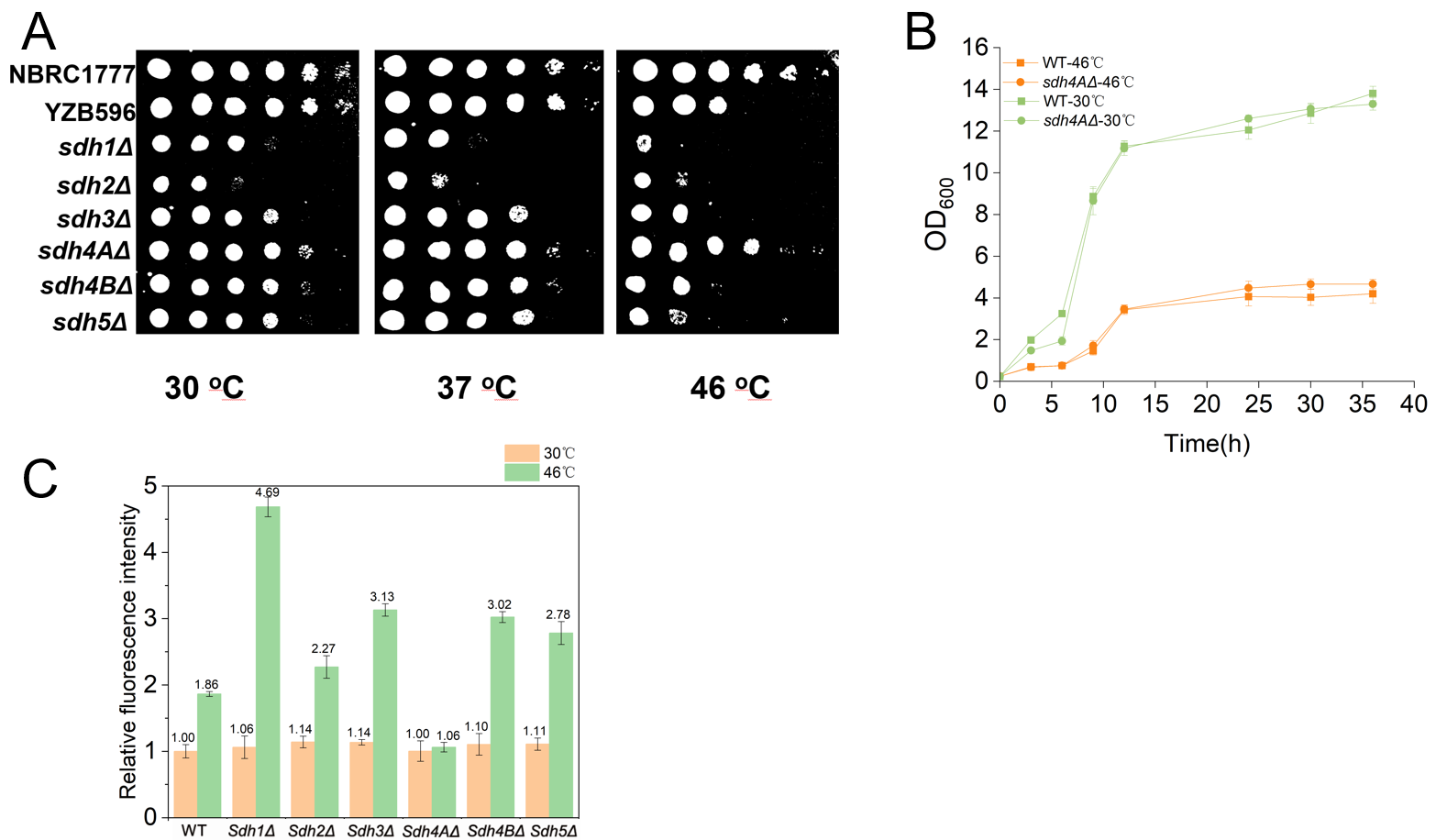


**Fig. S12 (A) Phenotypic plates of wild-type and (*SDH1*-*SDH5*) deficient strains at 30°C, 37°C, and 46°C. (B) Growth curves of WT and *sdh4A*Δ strains at 30°C and 46°C. (C) Reactive oxygen species (ROS) data measured by a microplate reader at 30°C and 46°C.**

**
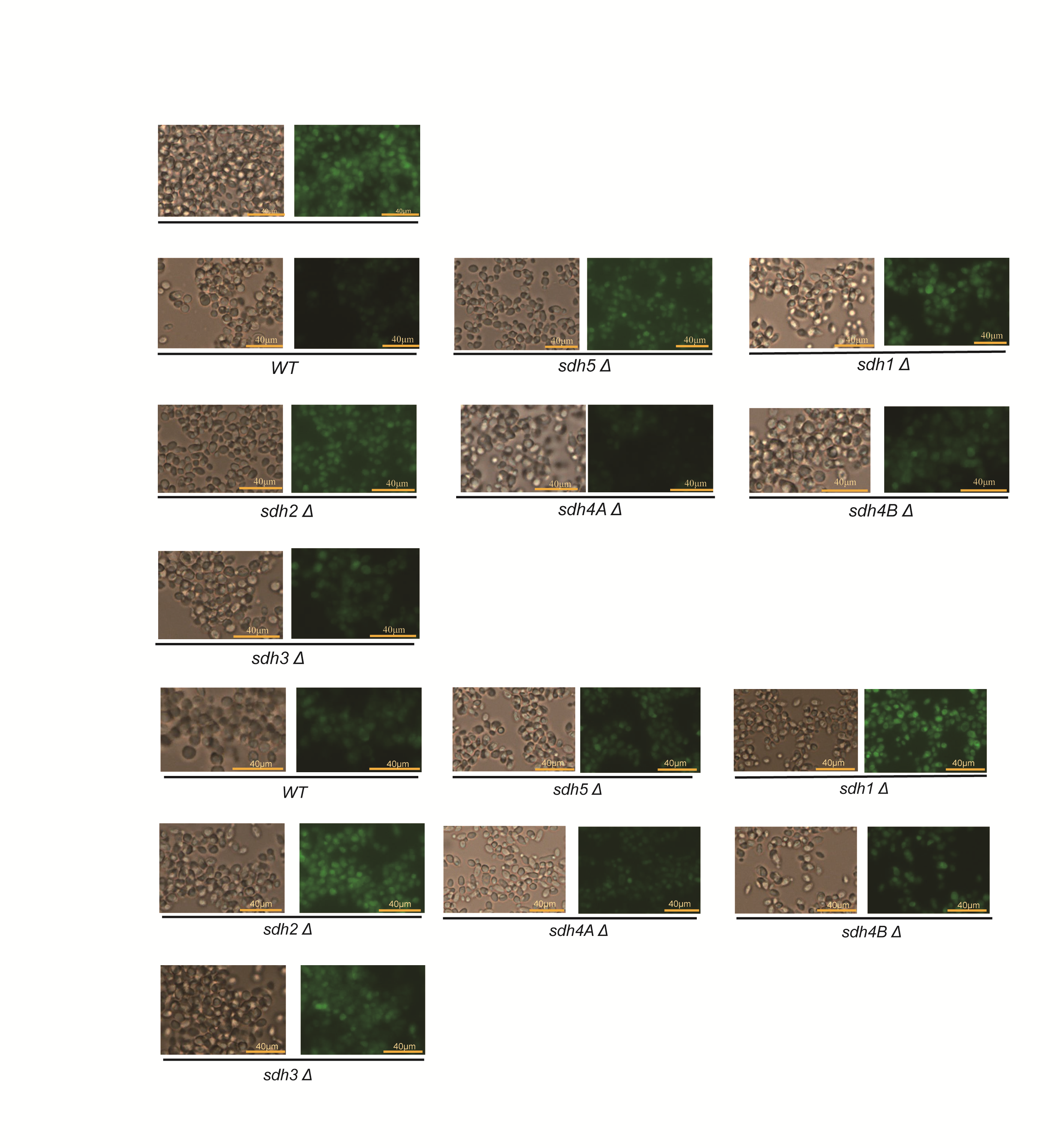
**

**Fig. S13 ROS levels were measured fluorescence microscopy (excitation: 504 nm; emission: 529 nm) of strains *sdh1Δ-sdh5Δ* at 30 ℃ (B) and 46 ℃ (C)**


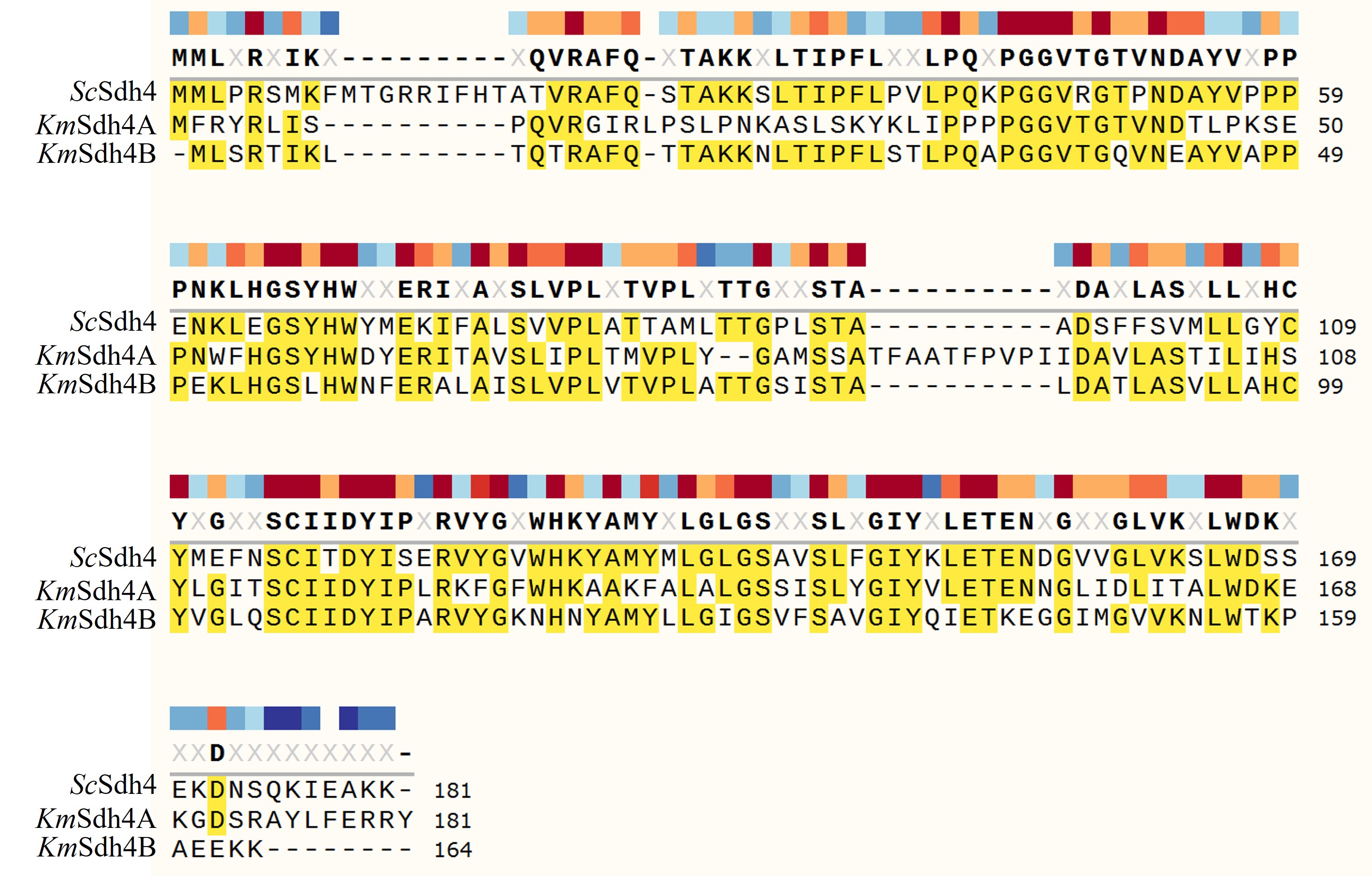


**Fig. S14 Results of protein sequence alignment among *Km*Sdh4A, *Km*Sdh4B, and *Sc*Sdh4**

**
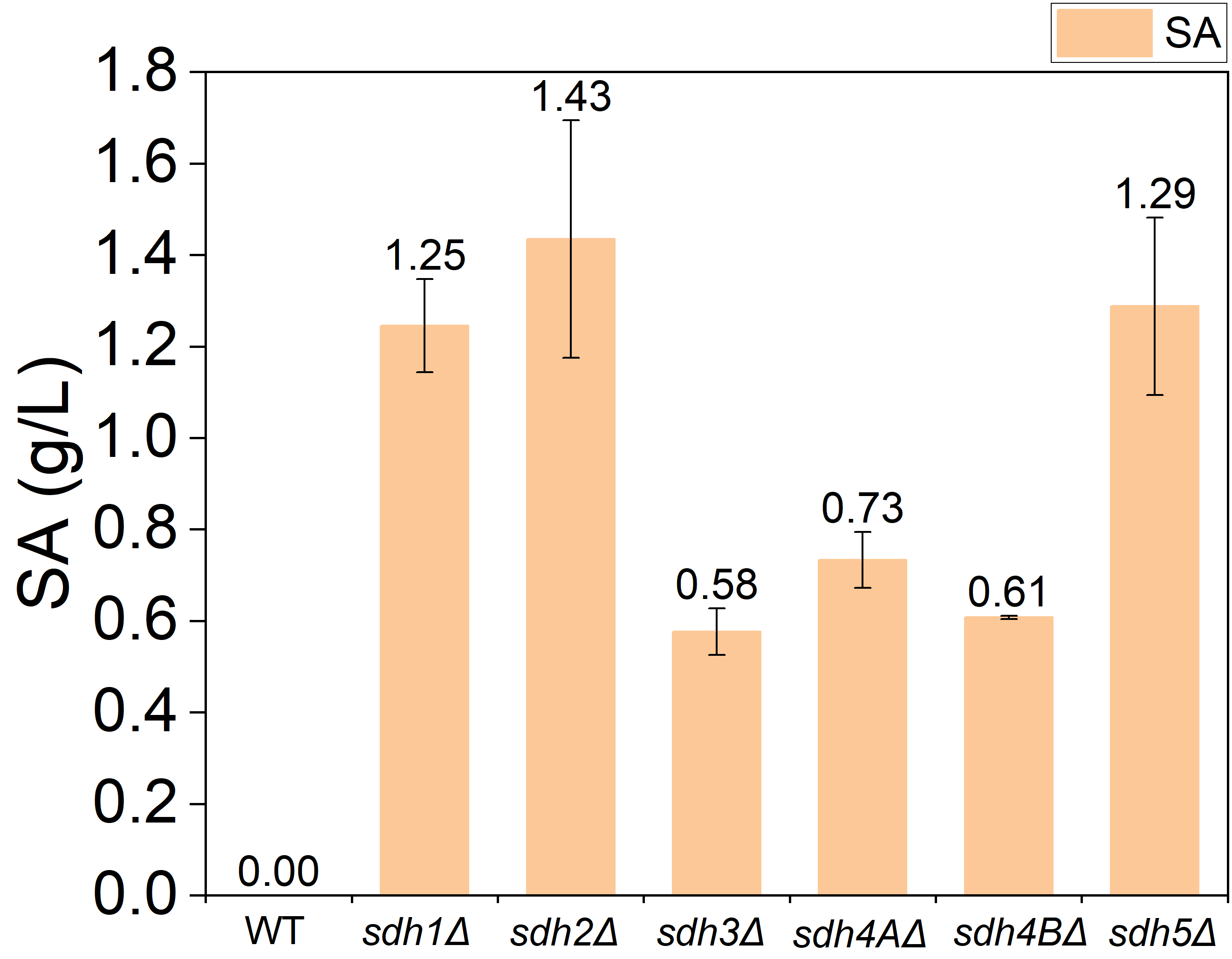
**

**Fig. S15 Knockout of SDH homologs in *K. marxianus* for succinic acid production using glycerol as substrate.**


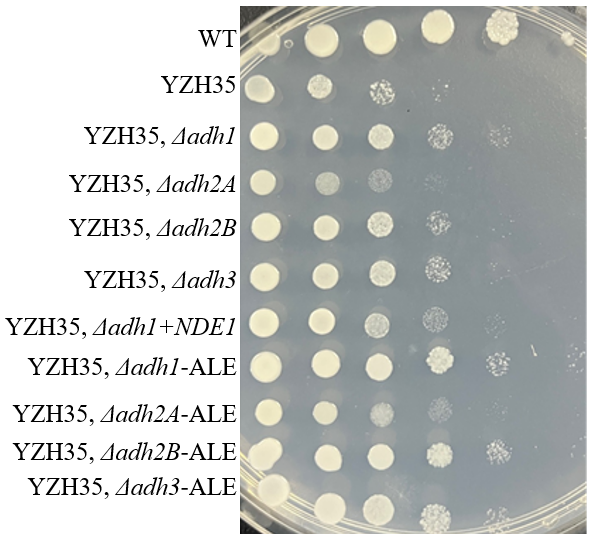


**Fig. S16 Impact of *SDH* and *ADH* genes knockouts, *NDE1* overexpression, and ALE on engineered strain growth**


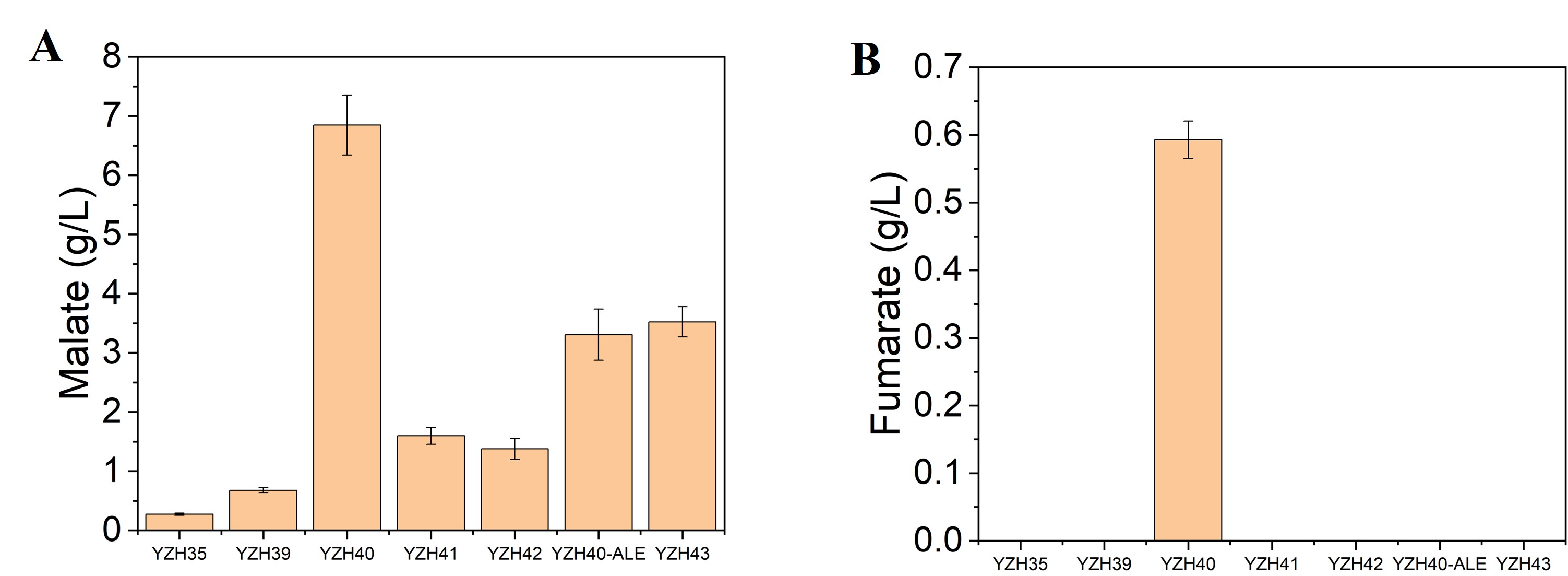


**Fig. S17 The accumulation status of malate and furmarate in different strains.**

**Table S1** Primers used in this study

| Primers | Sequences |
| --- | --- |
| M13-F | 5′-CGCCAGGGTTTTCCCAGTCACGAC-3′ |
| M13-R | 5′-AGCGGATAACAATTTCACACAGGA-3′ |
| 426P-F | 5′-PAATTTCTACTGTTGTAGATGGCCGGTACCCAATTCGCCC-3′ |
| 426P-R | 5′-ATTTAAAGTTCTTAGACCTCGCGTCTCTGATCATTTATCTTTCACTGC-3′ |
| 426T-F | 5′-CGTCTCTGAACTGATTGGTGCTTTTTTTGTTTTTTATGTCTTCG-3′ |
| 426T-R | 5′-PATCTACAACAGTAGAAATTAGCTCCAGCTTTTGTTCCC-3′ |
| NDE1-EcoRI-F | 5′-GACGTGAATTCCCACCATGTTTGTGAACAAGCATC-3′ |
| NDE1-NotI-R | 5′-ATAAGAATGCGGCCGCTTAAACAGAAGAATCTCTTC-3′ |
| RAD52-EcoRI-F | 5′-ACGTGAATTCGCCACCATGGATGATGGGCAGGGAAAC-3′ |
| RAD52-NotI-R | 5′-ATAAGAATGCGGCCGCCTATGAAATGTTTTTCCTGATG-3′ |
| EGFP-EcoRI-F | 5′-ACGTGAATTCGCCACCATGGTGAGCAAGGGCGAG-3′ |
| EGFP-NotI-R | 5′-ATAAGAATGCGGCCGCTTACTTGTACAGCTCGTCC-3′ |
| XYL1-F | 5′-ATGACATACCTCGCACCAAC-3′ |
| XYL1-R | 5′-TTAGATAAAGGTTGGGAATTC-3′ |
| EGFP-link1-F | 5′-ATACACCTCCCTCTCTGAGC-3′ |
| EGFP-link1-R | 5′-GATGAATTGAAAAGGTGGTACC-3′ |
| EGFP-link2-F | 5′-TTGCTCACCATTTTTTGTATC-3′ |
| EGFP-link2-R | 5′-GATACAAAAAATGGTGAGCAAGGGCGAGGAGCTGTTCACC-3′ |
| 60ADE2-SNR52p-F | 5′-ATGGACCAAAGAACTGTTGGTATTTTAGGTGGTGGCCAATTGGGCCGTATGATAGTGGATCTTTGAAAAGATAATGTATG-3′ |
| 60ADE2-SCUrA3p-R | 5′-GGCCTTAGTCAAGACCTCTTCCTCTTGTTTCATTAGGAATTTTTGCATCTTTACAAAGTAGCAGATTGTACTGAGAGTGC-3′ |
| 50ADE2-SNR52p-F | 5′-ATGGACCAAAGAACTGTTGGTATTTTAGGTGGTGGCCAATTGGGCCGTATTCTTTGAAAAGATAATGTATG-3′ |
| 50ADE2-SCUrA3p-R | 5′-GGCCTTAGTCAAGACCTCTTCCTCTTGTTTCATTAGGAATTTTTGCATCTGCAGATTGTACTGAGAGTGC-3′ |
| 40ADE2-SNR52p-F | 5′-ATGGACCAAAGAACTGTTGGTATTTTAGGTGGTGGCCAATTCTTTGAAAAGATAATGTATG-3′ |
| 40ADE2-SCUrA3p-R | 5′-GGCCTTAGTCAAGACCTCTTCCTCTTGTTTCATTAGGAATGCAGATTGTACTGAGAGTGC-3′ |
| pZB268-R | 5′-AGCTCCAGCTTTTGTTCCC-3′ |
| pZB268-F | 5′-AGAGACGCGAGGTCTAAGAAC-3′ |
| CZ-KmSRN52p-F | 5′-GGGAACAAAAGCTGGAGCTTGCCGCAAATAGGGCAGG-3′ |
| CZ-KmSRN52p-R | 5′-TTCTTAGACCTCGCGTCTCTGATTCGAACTGCGGACGTTG-3′ |
| CZ-KmTRNAGlyp-F | 5′-GGGAACAAAAGCTGGAGCTTTCCTTTGGAGTCCAGGATTG-3′ |
| CZ-KmTRNAGlyp-R | 5′-TTCTTAGACCTCGCGTCTCTTCTTTTTTGCAATTATTTTTATTTCAAATTTTAAAC-3′ |
| CZ-KmTEF1p-F | 5′-GGGAACAAAAGCTGGAGCTTCCCAAGACGTCCGTCTTAG-3′ |
| CZ-KmTEF1p-R | 5′-TTCTTAGACCTCGCGTCTCTCTTTAATGTTACTTCTCTTGGAGTTAG-3′ |
| CZ-PDC1p-F | 5′-TAAAGGGAACAAAAGCTGGAGCTCCGGGCTTCGGTGAAAATCG-3′ |
| CZ-PDC1p-R | 5′-CAAATTCTTGGTAGATGGACATTGCAATTATTTGGTTTGGGTGTG-3′ |
| SNR52p-F | 5′-TCTTTGAAAAGATAATGTATG-3′ |
| XDH-SNR52p-F | 5′-ATGACCAACACTCAAAAAGCCGTTGTTTTGAAGAAGCAAGTCTTTGAAAAGATAATGTATG-3′ |
| XDH-SCURA3p-F | 5′-TCATTCTGGACCATCAATGATAGTCTTGACAACTTCATTAGCAGATTGTACTGAGAGTGC-3′ |
| SCURA3p-F | 5′-GCAGATTGTACTGAGAGTGC-3′ |
| CR-TADE2p-SNR52-R1 | 5′-TTTGGTCCATGATTAAATCTACAACAGTAGAAATTATTTAAAGTTC-3′ |
| CR-TADE2p-CYC1T-F1 | 5′-TTAATCATGGACCAAAAATTTCTACTGTTGTAGATCGTCTCTG-3′ |
| CR-TADE2p-SNR52-R2 | 5′-ATGAATGATATTTTAAATCTACAACAGTAGAAATTATTTAAAGTTC-3′ |
| CR-TADE2p-CYC1T-F2 | 5′-TTAAAATATCATTCATAATTTCTACTGTTGTAGATCGTCTCTG-3′ |
| CR-TADE2p-SNR52-R3 | 5′-TGAAGATTGTAACCTAATCTACAACAGTAGAAATTATTTAAAGTTC-3′ |
| CR-TADE2p-CYC1T-F3 | 5′-TAGGTTACAATCTTCAAATTTCTACTGTTGTAGATCGTCTCTG-3′ |
| CR-TADE2p-SNR52-R4 | 5′-TATATTGATAGATGAGATCTACAACAGTAGAAATTATTTAAAGTTC-3′ |
| CR-TADE2p-CYC1T-F4 | 5′-CTCATCTATCAATATAAATTTCTACTGTTGTAGATCGTCTCTG-3′ |
| CR-TADE2p-SNR52-R5 | 5′-TTCAAGCTTTGCTCATATCTACAACAGTAGAAATTATTTAAAGTTC-3′ |
| CR-TADE2p-CYC1T-F5 | 5′-ATGAGCAAAGCTTGAAAATTTCTACTGTTGTAGATCGTCTCTG-3′ |
| ADE2-R1 | 5′-GAATTGAGTCCTTAGACTTGAC-3′ |
| ADE2-F1 | 5′-GTCAAGTCTAAGGACTCAATTCTTAGCGTTATTATGGGATCCG-3′ |
| 4LAC4-SCURA3p-F | 5′-GTTGCCGTGTTAAAGGATGATGCTGGTGTTCTAAAGGCAGGTCATGAAATTGCCTGGGGCGCAGATTGTACTGAGAGTGC-3′ |
| CR-ADE2p-SNR52-R1 | 5′-TGTAACCTACAAATATACGGAATATCTACAACAGTAGAAATTATTTAAAGTTC-3′ |
| CR-ADE2p-CYC1T-F1 | 5′-ATTCCGTATATTTGTAGGTTACAAATTTCTACTGTTGTAGATCGTCTCTG-3′ |
| CR-ADE2p-SNR52-R2 | 5′-GCAAAGCTTGAACATTTCACAGTATCTACAACAGTAGAAATTATTTAAAGTTC-3′ |
| CR-ADE2p-CYC1T-F2 | 5′-ACTGTGAAATGTTCAAGCTTTGCAATTTCTACTGTTGTAGATCGTCTCTG-3′ |
| CR-ADE2p-SNR52-R3 | 5′-ATGACGGCAGCAGGTCCTCAGTAATCTACAACAGTAGAAATTATTTAAAGTTC-3′ |
| CR-ADE2p-CYC1T-F3 | 5′-TACTGAGGACCTGCTGCCGTCATAATTTCTACTGTTGTAGATCGTCTCTG-3′ |
| CZ-CYC1T-R | 5′-CTCACTATAGGGCGAATTGGGTACCGGCCGCAAATTAAAGCCTTCGAGC-3′ |
| SNR52-F1 | 5′-CAAGGTGATTACATGTACGTTTGAAGTACAAC-3′ |
| CR-TRP1-SNR52-R | 5′-ATCCCCAAGGACATCACGTTGATATCTACAACAGTAGAAATTATTTAAAGTTC-3′ |
| CR-TRP1-CYC1T-F | 5′-ATCAACGTGATGTCCTTGGGGATAATTTCTACTGTTGTAGATCGTCTCTG-3′ |
| TRP1-M13-F | 5′-ATGCTCGTCAAGATCTGCGGCTTGCAGTCTGTTGAAGCTGCTCAAACAGCGCTGGATCGCAGGGTTTTCCCAGTCACGAC-3′ |
| TRP1-M13-R | 5′-TTAGATAGATAGACCCCTCGCCTGTGATATGAAGGCGGCGATCTTGGCCATGTCCTTATGAGCGGATAACAATTTCACAC-3′ |
| 414ARS-R | 5′-GCGATCCGTCTAAGAAACCATTATTATC-3′ |
| CR-ADE2-SNR52-R1 | 5′-GGATTTCAGCATAAATGGATATCATCTACAACAGTAGAAATTATTTAAAGTTC-3′ |
| CR-ADE2-CYC1T-F1 | 5′-GATATCCATTTATGCTGAAATCCAATTTCTACTGTTGTAGATCGTCTCTG-3′ |
| CR-ADE2-SNR52-R2 | 5′-AGAGTTCCAGACTCAGTGCAATTATCTACAACAGTAGAAATTATTTAAAGTTC-3′ |
| CR-ADE2-CYC1T-F2 | 5′-AATTGCACTGAGTCTGGAACTCTAATTTCTACTGTTGTAGATCGTCTCTG-3′ |
| CR-ADE2-SNR52-R3 | 5′-AATGGTTGAATGCGAACGTAAGTATCTACAACAGTAGAAATTATTTAAAGTTC-3′ |
| CR-ADE2-CYC1T-F3 | 5′-ACTTACGTTCGCATTCAACCATTAATTTCTACTGTTGTAGATCGTCTCTG-3′ |
| CR-XYL1-SNR52-R | 5′-CAACGTGCGTACCGCAGTCGAAAATCTACAACAGTAGAAATTATTTAAAGTTC-3′ |
| CR-XYL1-CYC1T-F | 5′-TTTCGACTGCGGTACGCACGTTGAATTTCTACTGTTGTAGATCGTCTCTG-3′ |
| CR-ADE2-SNR52-R4 | 5′-GCAAGGGTTCTGGATTTCAGCATATCTACAACAGTAGAAATTATTTAAAGTTC-3′ |
| CR-ADE2-CYC1T-F4 | 5′-ATGCTGAAATCCAGAACCCTTGCAATTTCTACTGTTGTAGATCGTCTCTG-3′ |
| 60ADE2-M13-F | 5′-ATGGACCAAAGAACTGTTGGTATTTTAGGTGGTGGCCAATTGGGCCGTATGATAGTGGAGAGGGTTTTCCCAGTCACGAC-3′ |
| 60ADE2-M13-R | 5′-GGCCTTAGTCAAGACCTCTTCCTCTTGTTTCATTAGGAATTTTTGCATCTTTACAAAGTAAGCGGATAACAATTTCACAC-3′ |
| ADE2-F | 5′-ATGGACCAAAGAACTGTTGG-3′ |
| ADE2-R | 5′-GGCCTTAGTCAAGACCTCTTC-3′ |
| 50ADE2-M13-F | 5′-ATGGACCAAAGAACTGTTGGTATTTTAGGTGGTGGCCAATTGGGCCGTATAGGGTTTTCCCAGTCACGAC-3′ |
| 50ADE2-M13-R | 5′-GGCCTTAGTCAAGACCTCTTCCTCTTGTTTCATTAGGAATTTTTGCATCTAGCGGATAACAATTTCACAC-3′ |
| 40ADE2-M13-F | 5′-ATGGACCAAAGAACTGTTGGTATTTTAGGTGGTGGCCAATAGGGTTTTCCCAGTCACGAC-3′ |
| 40ADE2-M13-R | 5′-GGCCTTAGTCAAGACCTCTTCCTCTTGTTTCATTAGGAATAGCGGATAACAATTTCACAC-3′ |
| 35ADE2-M13-F | 5′-ATGGACCAAAGAACTGTTGGTATTTTAGGTGGTGGAGGGTTTTCCCAGTCACGAC-3′ |
| 35ADE2-M13-R | 5′-GGCCTTAGTCAAGACCTCTTCCTCTTGTTTCATTAAGCGGATAACAATTTCACAC-3′ |
| 30ADE2-M13-F | 5′-ATGGACCAAAGAACTGTTGGTATTTTAGGTAGGGTTTTCCCAGTCACGAC-3′ |
| 30ADE2-M13-R | 5′-GGCCTTAGTCAAGACCTCTTCCTCTTGTTTAGCGGATAACAATTTCACAC-3′ |
| CR-1LAC4-SNR52-R | 5′-ATTTTAGTAAAGAAACGTCTCTGATCTACAACAGTAGAAATTATTTAAAGTTC-3′ |
| CR-1LAC4-CYC1T-F | 5′-CAGAGACGTTTCTTTACTAAAATAATTTCTACTGTTGTAGATCGTCTCTG-3′ |
| CR-SNR52-R2 | 5′-ATCTACAACAGTAGAAATTATTTAAAGTTCTTAGACCTCGCGTCTCTGATCATTTATCTTTCACTGCGG-3′ |
| CR-CYC1T-F2 | 5′-AATTTCTACTGTTGTAGATCGTCTCTGAACTGATTGGTGCTTTTTTTGTTTTTTATGTCTTC-3′ |
| CR-XYL2-SNR52-R1 | 5′-CTTAATACCAGAAGATTTTGTTGATCTACAACAGTAGAAATTATTTAAAGTTC-3′ |
| CR-XYL2-CYC1T-F1 | 5′-CAACAAAATCTTCTGGTATTAAGAATTTCTACTGTTGTAGATCGTCTCTG-3′ |
| CR-XYL2-SNR52-R2 | 5′-TCAACTCTAAAGATTATCCAAAGATCTACAACAGTAGAAATTATTTAAAGTTC-3′ |
| CR-XYL2-CYC1T-F2 | 5′-CTTTGGATAATCTTTAGAGTTGAAATTTCTACTGTTGTAGATCGTCTCTG-3′ |
| CR-SNR52-R | 5′-ATTTGAACAAATCTACAACAGTAGAAATTATTTAAAGTTCTTAGACCTCGCGTCTCTGATCATTTATCTTTCACTGCGG-3′ |
| CR-CYC1T-F | 5′-GTTCAGTATTATGAATTTCTACTGTTGTAGATCGTCTCTGAACTGATTGGTGCTTTTTTTGTTTTTTATGTCTTC-3′ |
| SDH1-F | 5'-ATGCAATTGTGCAGAAGAAATG-3' |
| SDH1-R | 5'-TCAGTAGGCTCTGATAGTTG-3' |
| SDH2-F | 5'-GTGTTGTCAGCTAGTGTAGTG-3' |
| SDH2-R | 5'-TACCCGTAAATTGAATAATG-3' |
| SDH3-F | 5'-CCAATTGTCAAGATAAATAA-3' |
| SDH3-R | 5'-TCCTTTGTCGTTTCTGCGGC-3' |
| SDH4A-F | 5'-AGGGGGTGTTTCTGATGTTG-3' |
| SDH4A-R | 5'-TTTTATGAGTAGACCGTTC-3' |
| SDH4B-F | 5'-CACGTGACACTGGTACACTG-3' |
| SDH4B-R | 5'-ATATGACGACGCGCACGCTG-3' |
| SDH5-F | 5'-GTGTTCCCGGGGTACTAAGTG-3' |
| SDH5-R | 5'-CCTGTTACATCCTTCTAG-3' |
| ACH1-F | 5'-ATGACAGTTTCTAGATTGTTG-3' |
| ACH1-R | 5'-GTATGGTGGGTTCACTGGCAT-3' |
| GPD1-F | 5'-ATGATTCAGTTTGGAAGATC-3' |
| GPD1-R | 5'-TTAGTGCTTCTTGACGTTG-3' |
| CR-SDH1-SNR52-R | 5'-CCTTGGTAACATCAACACCGGCGATCTACAACAGTAGAAATTATTTAAAGTTC-3' |
| CR-SDH1-CYC1T-F | 5'-CGCCGGTGTTGATGTTACCAAGGAATTTCTACTGTTGTAGATCGTCTCTG-3' |
| CR-SDH2-SNR52-R | 5'-ATGGAGTCCAGATACCCCAGCTGATCTACAACAGTAGAAATTATTTAAAGTTC-3' |
| CR-SDH2-CYC1T-F | 5'-CAGCTGGGGTATCTGGACTCCATAATTTCTACTGTTGTAGATCGTCTCTG-3' |
| CR-SDH3-SNR52-R | 5'-TCAAACCTAGACCGAACAAACTCATCTACAACAGTAGAAATTATTTAAAGTTC-3' |
| CR-SDH3-CYC1T-F | 5'-GAGTTTGTTCGGTCTAGGTTTGAAATTTCTACTGTTGTAGATCGTCTCTG-3' |
| CR-SDH4A-SNR52-R | 5'-TGCTAAGACAGCATCGATGATTGATCTACAACAGTAGAAATTATTTAAAGTTC-3' |
| CR-SDH4A-CYC1T-F | 5'-CAATCATCGATGCTGTCTTAGCAAATTTCTACTGTTGTAGATCGTCTCTG-3' |
| CR-SDH4B-SNR52-R | 5'-GAGCTCTTTCGAAATTCCAGTGTATCTACAACAGTAGAAATTATTTAAAGTTC-3' |
| CR-SDH4B-CYC1T-F | 5'-ACACTGGAATTTCGAAAGAGCTCAATTTCTACTGTTGTAGATCGTCTCTG-3' |
| CR-SDH5-SNR52-R | 5'-CCACGCTTACGGGACTGGTAGATATCTACAACAGTAGAAATTATTTAAAGTTC-3' |
| CR-SDH5-CYC1T-F | 5'-ATCTACCAGTCCCGTAAGCGTGGAATTTCTACTGTTGTAGATCGTCTCTG-3' |
| CR-ACH1-SNR52-R | 5'-TCACAATTTGGTGGAATTCTTCGATCTACAACAGTAGAAATTATTTAAAGTTC-3' |
| CR-ACH2-CYC1T-F | 5'-CGAAGAATTCCACCAAATTGTGAAATTTCTACTGTTGTAGATCGTCTCTG-3' |
| CR-GPD1-SNR52-R | 5'-AGAGAGTTGATATGTGGGTTTTCATCTACAACAGTAGAAATTATTTAAAGTTC-3' |
| CR-GPD-CYC1T-F | 5'-GAAAACCCACATATCAACTCTCTAATTTCTACTGTTGTAGATCGTCTCTG-3' |
| SDH1-M13-F | 5'-ATGCAATTGTGCAGAAGAAATGCCGCTAGAACTTTTTCTAACAGTGTGGTTTTGAGACACAGGGTTTTCCCAGTCACGAC-3' |
| SDH1-M13-R | 5'-TCAGTAGGCTCTGATAGTTGGTGGAACAGGAGGACATTCCTTTTCATCCAAGGTGGTAGCAGCGGATAACAATTTCACAC-3' |
| SDH2-M13-F | 5'-ATGTTCAACGTCATATTTAAGAGAGGTTTGGCCACTGAGGTCAAGGCTGCTGCTCCAAGAAGGGTTTTCCCAGTCACGAC-3' |
| SDH2-M13-R | 5'-CTAGTCCATAGCCAAAGCCTTCTTAATTTCAGCAATGGCTCTACCTGGGTTCAAACCCTTAGCGGATAACAATTTCACAC-3' |
| SDH3-M13-F | 5'-ATGTTCAAGAGTGTTTTGAAGCCAGGAATTATCAGCGGGGTCAGACCCAGCGTTTTGAATAGGGTTTTCCCAGTCACGAC-3' |
| SDH3-M13-R | 5'-TCACAAAGTGAGCAAGTAGGAACCTAGCACGGCAGTCAATGCCAAAACAGAGTACCCGGTAGCGGATAACAATTTCACAC-3' |
| SDH4A-M13-F | 5'-ATGTTTAGGTATAGACTGATATCTCCACAAGTTAGAGGGATTAGGCTCCCATCTTTGCCTAGGGTTTTCCCAGTCACGAC-3' |
| SDH4A-M13-R | 5'-TCAGTACCTTCTTTCAAATAAATATGCTCTTGAGTCACCCTTTTCTTTGTCCCAAAGAGCAGCGGATAACAATTTCACAC-3' |
| SDH4B-M13-F | 5'-ATGCTTTCCAGAACTATTAAATTGACTCAAACAAGAGCTTTCCAAACTACCGCCAAGAAGAGGGTTTTCCCAGTCACGAC-3' |
| SDH4B-M13-R | 5'-TCATTTCTTTTCTTCGGCAGGTTTGGTCCAGAGGTTCTTTACAACACCCATGATACCACCAGCGGATAACAATTTCACAC-3' |
| SDH5-M13-F | 5'-ATGATTGCACCAGGAATTTTGTTACAAGGAGCAAGGGCAGTGCCAAGGGCAGTGCCAAGGAGGGTTTTCCCAGTCACGAC-3' |
| SDH5-M13-R | 5'-TCAATACTTCGACAAGTCCGGCATGCTCAAGATCTCGCGGTCCTTGTTCTTGCTGAATTCAGCGGATAACAATTTCACAC-3' |
| ACH1-M13-F | 5'-ATGACAGTTTCTAGATTGTTGAAAGAGAGAGTGAGATATGCGCCATATTTGAAGAAGGTGAGGGTTTTCCCAGTCACGAC-3' |
| ACH1-M13-R | 5'-GTATGGTGGGTTCACTGGCATATCGATGTCGTGCAAACCTTCGAACGAAGGGGTTGCTGTAGCGGATAACAATTTCACAC-3' |
| GPD1-M13F-F | 5'-TAAAGCAAACTTCATCCATTCTTTCCAAGTCTGTTGAGCCAAAGGCCGACAGCCCATTCAAGGGTTTTCCCAGTCACGAC-3' |
| GPD1-M13R-R | 5'-GATACTTGACGTTTTCGTGACGTGTGTTGATGATTTCGGTCAACTTTTCGCCGTCAATTTAGCGGATAACAATTTCACAC-3' |
| GPD1-M13R-R | 5'-GATACTTGACGTTTTCGTGACGTGTGTTGATGATTTCGGTCAACTTTTCGCCGTCAATTTAGCGGATAACAATTTCACAC-3' |
| qPCR-Cas12a-F | 5'-CGCTAACAAGAACAAGGACAA-3' |
| qPCR-Cas12a-R | 5'-GTGATTGGACAGTGGAAGAAG-3' |
| qPCR-ACT1-F | 5'-CTTCAAGTGGACGATGGATG-3' |
| qPCR-ACT1-R | 5'-TGTCTGGATTGGTGGTTCTA-3' |
| ADH1-F | 5'-ATGGCTATTCCAGAAACTCAAAAG-3' |
| ADH1-R | 5'-TTATTTGGAAGTGTCAACGACAATTC-3' |
| ADH1-M13F-F | 5'-ATGGCTATTCCAGAAACTCAAAAGGGTGTTATCTTCTACGAAAACGGTGGTGAGTTGCAAAGGGTTTTCCCAGTCACGAC-3' |
| ADH1-M13R-R | 5'-TTATTTGGAAGTGTCAACGACAATTCTACCAATGATTTGGCCCTTGGACATCTTGTCGTAAGCGGATAACAATTTCACAC-3' |
| CR-ADH1-SNR52-R | 5'-TGAGCGTCTCTTGGCAAACCGACATCTACAACAGTAGAAATTATTTAAAGTTC-3' |
| CR-ADH1-CYC1T-F | 5'-GTCGGTTTGCCAAGAGACGCTCAAATTTCTACTGTTGTAGATCGTCTCTG-3' |
| ADH2A-F | 5'-ATGTCTATTCCAACTACTCAAAAG-3' |
| ADH2A-R | 5'-TTATTTGGAAGTGTCAACAACGTATC-3' |
| ADH2A-M13F-F | 5'-ATGTCTATTCCAACTACTCAAAAGGGTGTTATCTTCTACGAAAACGGTGGTCAATTGTACAGGGTTTTCCCAGTCACGAC-3' |
| ADH2A-M13R-R | 5'-TTATTTGGAAGTGTCAACAACGTATCTACCTAGAATCTTACCTTGTTCCATCTTTTCGTAAGCGGATAACAATTTCACAC-3' |
| CR-ADH2A-SNR52-R | 5'-TACCGTCGTTCTAGTCGGTTTGCATCTACAACAGTAGAAATTATTTAAAGTTC-3' |
| CR-ADH2A-CYC1T-F | 5'-GCAAACCGACTAGAACGACGGTAAATTTCTACTGTTGTAGATCGTCTCTG-3' |
| ADH2B-F | 5'-ATGTTTCGTAAGGTCACATCTG-3' |
| ADH2B-R | 5'-CTAAAAGTTAATAATAAGTTTCATAGCCTTTTC-3' |
| ADH2B-M13F-F | 5'-ATGTTTCGTAAGGTCACATCTGTTGCAGCAGCAAATTTAGGACGTTCTTTCAGCTCAAGTAGGGTTTTCCCAGTCACGAC-3' |
| ADH2B-M13R-R | 5'-CTAAAAGTTAATAATAAGTTTCATAGCCTTTTCTTCAGCAGCGTGCTTGAATACAGTGTAAGCGGATAACAATTTCACAC-3' |
| CR-ADH2B-SNR52-R | 5'-CTTGGAGAGTACAATTGTGATGTATCTACAACAGTAGAAATTATTTAAAGTTC-3' |
| CR-ADH2B-CYC1T-F | 5'-ACATCACAATTGTACTCTCCAAGAATTTCTACTGTTGTAGATCGTCTCTG-3' |
| ADH3-F | 5'-ATGCTTAGATTAACTAACGCCAG-3' |
| ADH3-R | 5'-TTATTTTTCAGTGTCGACGACG-3' |
| ADH3-M13F-F | 5'-ATGCTTAGATTAACTAACGCCAGAACATTTGTCAAGCCATTGCGCGCCACTGCATTCGGTAGGGTTTTCCCAGTCACGAC-3' |
| ADH3-M13R-R | 5'-TTATTTTTCAGTGTCGACGACGTATCTACCCAAGATCTTACCTTGTTCCATTAGTTCGTAAGCGGATAACAATTTCACAC-3' |
| CR-ADH3-SNR52-R | 5'-TTCTTCACCACCATCAATACCGAATCTACAACAGTAGAAATTATTTAAAGTTC-3' |
| CR-ADH3-CYC1T-F | 5'-TCGGTATTGATGGTGGTGAAGAAAATTTCTACTGTTGTAGATCGTCTCTG-3' |

**Table S2 Plasmids used in this study**

| strains | Relevant genotype | References |
| --- | --- | --- |
| yEUGAP | *ScURA3*, *P_ScTDH3_*, *T_ScTDH3_* |  |
| pMD18T-*Δ*Sc*URA3* | Amp, nonfunctional *ScURA3* |  |
| p414-TEF1p-Cas9-CYC1t | Amp, *P_ScTEF1_*-Cas9-*T_ScCYC1_* | Addgene |
| p426-SNR52p-gRNA.CAN1.Y-SUP4t | *P_ScSNR52_*-gRNA.CAN1.Y-*T_ScSUP4_* | Addgene |
| pCG | Amp, P*_T7_*-*EGFP*-T*_T7_* |  |
| pZB023 | Amp, *ScURA3*, pUC19-*P_KmPGK1_*-*T_ScTDH3_* |  |
| pZB047 | Amp, pZB023-*KmNDE1* | This study |
| pZB089 | Amp, pUC19- *P_ScSNR52_* |  |
| pZB090 | Amp, pUC19-gRNA.CAN1.Y- *T_ScSUP4_*- *_ScCYC1_* |  |
| pZB182 | Amp, pZB023-*KmRad52* | This study |
| pZB191 | Amp, pZB023-*EGFP* | This study |
| pZB210 | Amp, *P_ScTEF1_*-*Cas9^D10A, H840A^*-*T_ScCYC1_* | This study |
| pZB211 | Amp, *P_ScSNR52_*-DR | This study |
| pZB212 | Amp, DR*-T_ScCYC1_* | This study |
| pZB214 | Amp, *P_ScTEF1_*-*Cas12a*-*_ScCYC1_* | This study |
| pZB260 | Amp, *P_KmINU1_*-*Cas12a*-*_ScCYC1_* | This study |
| pZB261 | Amp, *P_KmGDH2_*-Cas12a-*_ScCYC1_* | This study |
| pZB273 | Amp, *P_KmPDC1_*-Cas12a-*_ScCYC1_* | This study |
| pZB281 | Amp, *P_KmSNR52_*-DR | This study |
| pZB282 | Amp, *P_KmTEF1_*-DR | This study |
| pZB283 | Amp, *P_KmtRNA_^GLY^*-DR | This study |

**Table S3 Strains used in this study**

| Strains | Relevant genotype | References |
| --- | --- | --- |
| *K. marxianus* NBRC1777 | Wildtype, from NBRC | NBRC |
| YZB040 | NBRC1777, *ΔKmura3* | Error: Reference source not found |
| YZB100 | YZB040, *Δku70*::*Scura3* | Error: Reference source not found |
| YZB101 | YZB100, *ΔScura3* | Error: Reference source not found |
| YZB358 | YZB101, *Δxyl2*:: *P_KmPGK1_*- *Rad52*-*URA3* expression cassette | This study |
| YZB359 | YZB358, *ΔScura3* | This study |
| YZB567 | YZB101, *Δxyl1*:: *P_KmINU1_*-Cas12a-*URA3* expression cassette | This study |
| YZB568 | YZB101, *Δxyl1*:: *P_KmGDH2_*-Cas12a-*URA3* expression cassette | This study |
| YZB569 | YZB101, *Δxyl1*:: *P_ScTEF1_*-Cas12a-*URA3* expression cassette | This study |
| YZB570 | YZB567, *ΔScura3* | This study |
| YZB571 | YZB568, *ΔScura3* | This study |
| YZB572 | YZB569, *ΔScura3* | This study |
| YZB596 | YZB101, *Δxyl1*:: *P_KmPDC1_*-Cas12a -*URA3* expression cassette | This study |
| YZB599 | YZB596, *ΔScura3* | This study |
| YZH01 | YZB599, *Δsdh5* | This study |
| YZH02 | YZB599, *Δsdh1* | This study |
| YZH03 | YZB599, *Δsdh2* | This study |
| YZH04 | YZB599, *Δsdh4A* | This study |
| YZH05 | YZB599, *Δsdh4B* | This study |
| YZH06 | YZB599, *Δsdh3* | This study |
| YZH07 | YZH06, *ΔScura3* | This study |
| YZH08 | YZH07, (*Δsdh3*, *Δsdh4A*) | This study |
| YZH10 | YZH09*,* (*Δsdh3*, *Δsdh4A*, *Δsdh4B)* | This study |
| YZH11 | YZH11, *ΔScura3* | This study |
| YZH12 | YZH11, (*Δsdh3, Δsdh4A, Δsdh4B, Δsdh5)* | This study |
| YZH13 | YZH12, *ΔScura3* | This study |
| YZH14 | YZH02, *ΔScura3* | This study |
| YZH15 | YZH14, *Δsdh2* | This study |
| YZH16 | YZH13, (*Δsdh3, Δsdh4A, Δsdh4B, Δsdh5, Δsdh2*) | This study |
| YZH17 | YZH13, (*Δsdh3, Δsdh4A, Δsdh4B, Δsdh5, ΔSdh1*) | This study |
| YZH18 | YZH17, *ΔScura3* | This study |
| YZH19 | YZH18, (*Δsdh3, Δsdh4A, Δsdh4B, Δsdh5, ΔSdh1, Δsdh2*) | This study |
| YZH20 | YZH15, (*ΔScura3*) | This study |
| YZH21 | YZH20, (*Δsdh1, Δsdh3*) | This study |
| YZH22 | YZH21, *ΔScura3* | This study |
| YZH23 | YZH22, (*Δsdh1, Δsdh3, Δsdh5*) | This study |
| YZH24 | YZH23, *ΔScura3* | This study |
| YZH25 | YZH24, (*Δsdh1, Δsdh3, Δsdh5, Δsdh4A*) | This study |
| YZH26 | YZH25, *ΔScura3*) | This study |
| YZH27 | YZH26, (*Δsdh1, Δsdh3, Δsdh5, Δsdh4A, Δsdh2*) | This study |
| YZH28 | YZH27, *ΔScura3* | This study |
| YZH29 | YZB599, *Δach1* | This study |
| YZH30 | YZH29, *ΔScura3* | This study |
| YZH31 | YZH28*,* (*Δsdh1, Δsdh3, Δsdh5, Δsdh4A, Δsdh2, Δach1*) | This study |
| YZH32 | YZH31, *ΔScura3* | This study |
| YZH33 | YZH30, (*Δach1, Δgpd1)* | This study |
| YZH34 | YZH33, *ΔScura3* | This study |
| YZH35 | YZH32, (*Δsdh1, Δsdh3, Δsdh5, Δsdh4A, Δsdh2, Δach1, Δgpd1*) | This study |
| YZH36 | YZH35, *ΔScura3* | This study |
| YZH37 | YZH34, (*Δach1, Δgpd1, Δsdh3*) | This study |
| YZH38 | YZH34, (*Δach1, Δgpd1, Δsdh1*) | This study |
| YZH39 | YZH36(*Δsdh1, Δsdh3, Δsdh5, Δsdh4A, Δsdh2, Δach1, Δgpd1, Δadh1*) | This study |
| YZH40 | YZH36, (*Δsdh1, Δsdh3, Δsdh5, Δsdh4A, Δsdh2, Δach1, Δgpd1, Δadh2A*) | This study |
| YZH41 | YZH36, (*Δsdh1, Δsdh3, Δsdh5, Δsdh4A, Δsdh2, Δach1, Δgpd1, Δadh2B*) | This study |
| YZH42 | YZH36, (*Δsdh1, Δsdh3, Δsdh5, Δsdh4A, Δsdh2, Δach1, Δgpd1, Δadh3*) | This study |
| YZH43 | YZH36, (*Δsdh1, Δsdh3, Δsdh5, Δsdh4A, Δsdh2, Δach1, Δgpd1, Δadh2A:: P_KmPGK1_*-*NDE1- T_ScTDH3_*) | This study |

**Table S4 The gene sequences used in this study**

| *FnCAS12a* | ATGTCCATCTACCAAGAATTTGTTAACAAGTACTCCTTGTCCAAGACCTTGAGATTCGAATTGATCCCACAAGGTAAAACCTTGGAAAACATCAAGGCTAGAGGTTTGATCTTGGATGACGAAAAGAGAGCTAAGGATTACAAGAAGGCTAAGCAAATCATCGACAAGTACCACCAATTCTTCATCGAAGAAATCTTGTCTTCTGTTTGTATCTCCGAAGATTTGTTGCAAAACTACTCTGACGTTTACTTCAAGTTGAAGAAGTCTGATGACGACAACTTGCAAAAGGATTTCAAGTCCGCTAAGGACACCATCAAGAAGCAAATCTCTGAATACATCAAGGACTCTGAAAAGTTCAAGAACTTGTTCAACCAAAACTTGATCGATGCTAAGAAGGGTCAAGAATCTGACTTGATCTTGTGGTTGAAGCAATCTAAGGACAACGGTATCGAATTGTTCAAGGCTAACTCCGATATCACCGATATCGATGAAGCTCTTGAAATCATCAAGTCCTTCAAGGGTTGGACTACTTACTTCAAGGGTTTCCACGAAAACAGAAAGAACGTTTACTCCTCTAACGATATCCCAACTTCCATCATCTACAGAATCGTTGACGACAACTTGCCAAAGTTCTTGGAAAACAAGGCTAAGTACGAATCTTTGAAGGATAAGGCTCCAGAAGCTATCAACTACGAACAAATCAAGAAGGACTTGGCTGAAGAATTGACTTTCGACATCGACTACAAGACCTCTGAAGTTAACCAAAGAGTTTTCTCCTTGGACGAAGTTTTCGAAATCGCTAACTTCAACAACTACTTGAACCAATCCGGTATCACCAAGTTCAACACTATCATCGGTGGTAAATTCGTTAACGGTGAAAACACCAAGAGAAAGGGTATCAACGAATACATCAACTTGTACTCCCAACAAATCAACGACAAGACCTTGAAGAAGTACAAGATGTCCGTTTTGTTCAAGCAAATCTTGTCCGATACCGAATCCAAGTCCTTCGTTATCGACAAGTTGGAAGATGACTCCGATGTTGTTACTACTATGCAATCCTTCTACGAACAAATTGCTGCTTTCAAGACTGTTGAAGAAAAGTCCATCAAGGAAACTTTGTCTTTGTTGTTCGACGACTTGAAGGCTCAAAAGTTGGATTTGTCCAAGATCTACTTCAAGAACGACAAGTCTTTGACTGACTTGTCCCAACAAGTTTTCGATGACTACTCCGTTATCGGTACTGCTGTTTTGGAATACATCACCCAACAAATCGCTCCAAAGAACTTGGATAACCCATCTAAGAAGGAACAAGAATTGATCGCTAAGAAGACTGAAAAGGCTAAGTACTTGTCCTTGGAAACCATCAAGTTGGCTTTGGAAGAGTTTAATAAGCACAGAGATATCGATAAGCAATGTAGATTCGAAGAAATCTTGGCTAACTTCGCTGCTATCCCAATGATCTTCGATGAAATCGCTCAAAACAAGGACAACTTGGCTCAAATCTCCATCAAGTACCAAAACCAAGGTAAAAAGGATTTGTTGCAGGCTTCTGCTGAAGACGACGTTAAGGCTATCAAGGACTTGTTGGATCAAACCAACAACTTGTTGCACAAGTTGAAGATCTTCCACATCTCCCAATCTGAAGATAAGGCTAACATCTTGGATAAGGATGAACACTTCTACTTGGTTTTCGAAGAATGTTACTTCGAATTGGCTAACATCGTTCCATTGTACAACAAGATCAGAAACTACATCACTCAAAAGCCATACTCCGACGAAAAGTTCAAGTTGAACTTCGAAAACTCCACCTTGGCTAACGGTTGGGATAAGAACAAGGAACCAGATAACACCGCTATCTTGTTCATCAAGGACGATAAGTACTACTTGGGTGTTATGAACAAGAAGAACAACAAGATCTTCGACGATAAGGCTATCAAGGAAAACAAGGGTGAAGGTTACAAGAAGATCGTTTACAAGTTGTTGCCAGGTGCTAACAAGATGTTGCCAAAGGTTTTCTTCTCCGCTAAGTCCATCAAGTTCTACAACCCATCCGAAGACATCTTGAGAATCAGAAACCACTCCACTCACACTAAGAACGGTTCTCCACAAAAGGGTTACGAAAAGTTCGAGTTTAATATCGAAGACTGTAGAAAGTTCATCGACTTCTACAAGCAATCCATCTCCAAGCACCCAGAATGGAAGGACTTCGGTTTCAGATTCTCTGACACCCAAAGATACAACTCTATCGACGAATTTTACAGAGAAGTTGAAAACCAAGGTTACAAGTTGACTTTCGAAAACATCTCCGAATCCTACATCGATTCCGTTGTTAACCAAGGTAAATTGTACTTGTTCCAAATCTACAACAAGGATTTCTCTGCTTACTCCAAGGGTAGACCAAACTTGCACACCTTGTACTGGAAGGCTTTGTTCGACGAAAGAAACTTGCAAGATGTTGTTTACAAGTTGAACGGTGAAGCTGAATTGTTCTACAGAAAGCAATCTATCCCAAAGAAGATCACCCACCCAGCTAAGGAAGCTATCGCTAACAAGAACAAGGACAATCCAAAGAAGGAATCCGTTTTCGAATACGATTTGATCAAGGACAAGAGATTCACCGAAGATAAGTTCTTCTTCCACTGTCCAATCACCATCAACTTCAAGTCTTCTGGTGCTAACAAGTTCAACGATGAAATCAACTTGTTGTTGAAGGAAAAGGCTAACGATGTTCACATCTTGTCTATCGATAGAGGTGAAAGACACTTGGCTTACTACACCTTGGTTGACGGTAAAGGTAACATCATCAAGCAAGATACCTTCAACATCATCGGTAACGACAGAATGAAGACCAACTACCACGACAAGTTGGCTGCTATCGAAAAGGACAGAGATTCTGCTAGAAAGGATTGGAAGAAGATCAACAACATCAAGGAAATGAAGGAAGGTTACTTGTCCCAAGTTGTTCACGAAATCGCTAAGTTGGTTATCGAATACAACGCTATCGTTGTTTTCGAAGATTTGAACTTCGGTTTCAAGAGAGGTAGATTCAAGGTTGAAAAGCAAGTTTACCAAAAGTTGGAAAAGATGTTGATCGAAAAGTTGAACTACTTGGTTTTTAAGGACAACGAATTTGATAAGACTGGTGGTGTTTTGAGAGCTTACCAATTGACCGCTCCATTCGAAACCTTCAAGAAGATGGGTAAACAAACCGGTATCATCTACTACGTTCCAGCTGGTTTCACCTCTAAGATCTGTCCAGTTACTGGTTTCGTTAACCAATTGTACCCAAAGTACGAATCCGTTTCTAAGTCTCAAGAATTTTTCTCTAAGTTCGACAAGATCTGTTACAACTTGGACAAGGGTTACTTCGAATTTTCTTTCGATTACAAGAACTTCGGTGACAAGGCTGCTAAGGGTAAATGGACCATCGCTTCCTTCGGTTCTCGTTTGATCAACTTCAGAAACTCCGACAAGAACCACAACTGGGACACTAGAGAAGTTTACCCAACTAAGGAATTGGAAAAGTTGTTGAAGGACTACTCCATCGAATACGGTCACGGTGAATGTATCAAGGCTGCTATCTGTGGTGAATCTGATAAGAAGTTCTTCGCTAAGTTGACTTCTGTTTTGAACACCATCTTGCAAATGAGAAACTCCAAGACTGGTACTGAATTGGATTACTTGATCTCTCCAGTTGCTGATGTTAACGGTAACTTCTTCGATTCCAGACAAGCTCCAAAGAATATGCCACAAGATGCTGATGCTAACGGTGCTTACCACATCGGTTTGAAGGGTTTGATGTTGTTGGGTAGAATCAAGAACAACCAAGAAGGTAAAAAGTTGAACTTGGTTATCAAGAACGAAGAATACTTCGAATTTGTTCAAAACAGAAACAACTAA |
| --- | --- |
| *KmADE2* | ATGGACCAAAGAACTGTTGGTATTTTAGGTGGTGGCCAATTGGGCCGTATGATAGTGGAGGCCGCTCACAGATTGAACGTCAAGACTATCATATTGGATGCTGAAAACTCGCCAGCAAAGCAGATCAATGCTTTGTCCGAACATGTTAACGGTTCGTTTTCAAAACCAGAAGATATCAAGAAAATCGCTTCCATGTGCGATGTTTTAACTGTGGAAATTGAGCATGTCGATGTCCCAACTCTTAAGCAGGTCCAAATCGACCATCCAAACTTGAAGATATATCCAGACCCAGAAACAATTGCCTTAATTCAAGATAAGTTCACTCAAAAGCAATACTTGATCAAGAACAATATTAGCGTTGCTGAATCTGTTTCAGTCGAAAGTGACGAAAAATCCTTGGCTGAGGTCGGCTCTCAATTTGGATATCCATTTATGCTGAAATCCAGAACCCTTGCTTATGATGGCAGAGGAAATTTTGTAGTCAAGTCTAAGGACTCAATTCCAGAAGCACTCAGTGTGTTAAAAGATCGCCCGCTATACGCAGAAAAATGGGCACCATTCGTAAAGGAACTAGCAGTGATGGTAGTAAGATCCCCAGAAAATGCAGTGTTTTCTTATTCGACGGTAGAGACCATCCACAAAGATAACATCTGTCACACCTGTTACGCCCCAGCTAGAGTTCCAGACTCAGTGCAATTGAAAGCAAAGTTGTTGGCTGAACAAGCAGTTGCAGCATTCCCAGGTGCTGGTATTTTCGGTGTAGAGATGTTCTACTTAGAAGACGGGGAGTTATTGATTAACGAGATCGCGCCAAGACCCCATAATTCCGGCCATTATACCATCGACGCATGCGTCACCTCTCAATTCGAAGCCCATATCAGATCTATATTAAATTTGCCTATGCCAAAAGAATTCACTTCCTTGTCCACAACTAACACTAACGCTATTATGTTGAACATATTAGGAGATAACGAAGTGAAGAATAATGAACTAAAAATCTGTGAAAGAGCACTAAATACGCCGGGTGCATCTGTGTACTTATACGGTAAGGAATCAAGACCAAAAAGAAAAATGGGTCATATTAACATTATTGGTTCTTCAATGGTTGAATGCGAACGTAAGTTAAATTTCATCCAGTACGGTGAAGAAACTTATAAAAACAGCGAAGCTAGCAAATCTGCCGTTCCTGGTACTTCTTCGAAACCATTAGTTAGCGTTATTATGGGATCCGATTCTGATCTTCCTGTCATGTCTAAAGCCTGCGATATTCTCAATGATTTTAACGTCCCATACGAGGTTACTATCGTTTCGGCCCATAGAACTCCTCACAGAATGTCCAAGTTCGCTATCGAAGCAGGTAACCGTGGTATAAAGGCGATCATTGCTGGTGCAGGAGGTGCTGCTCATTTGCCAGGTATGGTTGCAGCCATGACACCACTACCTGTTATCGGTGTTCCTGTCAAGGGATCATGTTTGGATGGTGTTGATTCTTTGCATTCTATCGTCCAAATGCCAAGAGGTATACCAGTTGCTACTGTTGCAATCAACAATAGTACCAACGCTGCTTTACTAGCTATTAGAATTCTTGGTGCTTATGATTCTAGTTACTTTGTAAAGATGCAAAAATTCCTAATGAAACAAGAGGAAGAGGTCTTGACTAAGGCCGAAAAGATTGAAAATATCGGATACCAAGAATATTTGAGGAAATAA |
| *KmXYL1* | ATGACATACCTCGCACCAACAGTTACCTTGAACAATGGATCCAAGATGCCGCTAGTCGGCTTGGGATGCTGGAAAATCCCAAACGAAGTGTGTGCCGAACAGGTGTACGAAGCCATCAAGTTGGGCTACCGCTTGTTCGACGGCGCGCAGGACTACGCCAACGAAAAAGAGGTGGGCCAAGGTATTAACAGAGCCATCAAGGAAGGAATCGTCAAGAGAGAAGACTTGGTCGTCGTTTCTAAGTTGTGGAACAGTTTCCACCACCCAGACAACGTGCGTACCGCAGTCGAAAGAACTTTGAACGACTTGCAATTGGACTACTTGGACTTGTTCTACATCCATTTCCCATTGGCTTTCAAGTTCGTGCCACTAGACGAGAAGTACCCTCCAGGTTTCTACACAGGTAAGGACAATTTCGCCAAGGAAATCATCGAAGAGGAGCCTGTCCCAATCTTGGACACCTACAGAGCCCTTGAGAAGTTGGTCGACGAAGGTTTGATCAAATCTTTGGGTATCTCAAACTTTTCGGGTGCATTGATCCAGGACTTGTTGCGTGGCGCCCGTATCAAGCCAGTCGCCTTGCAGATCGAACACCACCCATACTTGGTCCAGGACCGCTTGATCACGTACGCCCAAAAGGTGGGCTTGCAAGTCGTCGCCTACTCCAGTTTCGGCCCACTATCCTTTGTCGAGTTGAACAACGAAAAGGCCTTGCACACAAAGACTTTGTTCGAAAACGACACCATCAAGGCCATCGCTCAAAAACACAACGTCACCCCATCCCACGTCTTGTTGAAGTGGTCCACCCAACGTGGTATCGCCGTCATTCCAAAGTCCTCCAAGAAGGAACGTCTCCTCGAGAACTTGAAGATCGAAGAGACCTTTACCTTGTCCGACGAAGAGATCAAGGAGATCAACGGCTTGGACCAGGGATTGAGATTTAACGACCCATGGGACTGGTTGGGCAACGAATTCCCAACCTTTATCTAA |
| *KmXYL2* | ATGACCAACACTCAAAAAGCCGTTGTTTTGAAGAAGCAAGGAGAGATTGCTTTCGAAGAGAGACCTGTCCCAGAAATTAAAGATCCTCATTACGTGAAGGTTCACATCAAGAAGACAGGTATTTGCGGTTCTGATGTTCACTACTATACTCATGGTGCTATTGGTGACTTTGTTGTCAAAGAACCAATGGTTCTAGGTCATGAATCAAGTGGTGTTGTTGTTGAAGTAGGCGAAGCAGTTACCCTGGTCAAGGTTGGCGATCGTGTCGCTGTAGAGCCAGGTGTTCCCAGCAGATACTCTGATGAGACTAAGTCCGGTCATTACAACTTATGTCCTCACATGGAATTCGCTGCGACCCCTCCAATTGATGGTACTTTGGTTAAGTACTACTTAATACCAGAAGATTTTGTTGTAAAGTTACCAGATCATGTTTCACTTGAAGAGGGTGCTTGTATTGAGCCTTTGTCTGTTGGTGTGCACGCTAACAGACTTGCCGGTACTGGATTTGGTAAAAAAGTTGTTATTTTTGGCGCAGGCCCTGTTGGTTTAGTTACTGGTAACGTTGCCTCTGCTTTTGGCGCCTCTGATGTTGTATACGTGGATGTTTTTGAACACAAATTGAAGAGAGCTAAAGAATTCGGTGGTACTCAGATTATCAACTCTAAAGATTATCCAAAGGAAGATGATTTAGTCAAGGCAATTCAAGATAAGTTGGGCGGTAAATCGCCTGAAATCGCTATTGATTGCTCAGGAGCTGAGGTTTGTATCAGATCGGCAATTAAAGTCTTAAAAGTTGGCGGTACCTTCGTTCAAGTGGGTATGGGTAGAGATGATGTTAACTTCCCTATCACTCTTATCATTACAAAGGAATTGCGTGTTTTGGGATCCTTTAGATATTACTTTGATGACTACAACATCGCTGTCAAGTTAGTGTCTGAAGGCAAAGTCAATGCCAAAGCTTTGATTACTCACACCTTCAAGTTTGACGAAGCAATCGATGCTTATAATTTCAACAGAGATCACGGTAATGAAGTTGTCAAGACTATCATTGATGGTCCAGAATGA |
| *KmTRP1* | ATGCTCGTCAAGATCTGCGGCTTGCAGTCTGTTGAAGCTGCTCAAACAGCGCTGGATCGCGGCGCAGACCTGCTGGGAGTCATATGCGTCCCCAACAGGAAACGCACCGTCACGCCAGCAACAGCAAAACAAATCTCACAACTGGTTCACCAGGGCAATCATTCCCAAGGGAATCACCAGGCCAGGCTTGTCGGGGTGTTCCGGAACCAGCCTCTGGAAGAAGTGCTCGCCCTGTACCACGAATACAACCTAGACGTTATCCAGCTTCACGGCAACGAAGATGTGGTCCAATGGAGAAAATGGATCCCCAAGGACATCACGTTGATCAAGGCGTTCCAGTTCCCTGGCGACTGCGACGTGGTGACATCGCCAGCGGTCGCACAGCTCCAGCTGGAAAACGTGCTGGTGCTGTTCGACTCGGGCGAAGGTGGCACGGGCCAGCAGCTCGACTGGAACGGCATGGCCAGCTGGTGTCAGAACCAAGGTAATACCACCCGCTTCATACTCGCGGGAGGACTCACCCCAGATAACGTGCGCCACGCCATCACAAGCCTCGCGCCCCATGCCATCGGAGTCGACGTCAGCGGAGGTGTCGAGACTAACGGCCATAAGGACATGGCCAAGATCGCCGCCTTCATATCACAGGCGAGGGGTCTATCTATCTAA |
| *P_KmGDH2_* | TCACTCATGGAGAGGGCGGGAAGGGTTTTGAACGCGAGGCTTTTTCCACTTTGACACATTTTTTCTTAGTGAAAACTGAAGAAAGAAGTAGTAGGGTTGAGAGTAGAGTAGAAGTGGAATGTCAATAATGACTTTAGCAGGCTCTAAAGCAAAAAGAAAACAGACAAACCAAAATTAAAATGCCTTCAAAATCACTCAATATGTAAACAAACACCCAATTAATTACATACGCATTACTGTCGCTACGATACTCCACTATTGCGCTTCTCCGAACCTCAATGTTATTGTTTGTCCCACACAGTCATGATCAAGTTCTAAGTACTGATCATTAACTAAACAAAATCACTAACAACACACACTAGAAAGTCCTTACATAGAAATTCCCGAAGAATGTCAAAGCGCTTCTATTTACTTACTCAACTGTTCACTTCCGACAAGGATGGAAGGGGAGAAACAATAGTGAAAAAACATATATAAAGGCTGTGTAACATGTGCTCGGAAAGAAAACATGTTGGGATTAATTATTACTCTTGTTCTATAATTAGGAATTTGCTGTCCATAAACGGTATAGTAACTGAAAGACTTCTCACTATTTAATTGGTCAATATCCAATTGGTTTAAAGACAGACAGACCACAAATACGAAAATTTAATTTAACCGCCTTGCTAGCAACTCTAATCCTGTTCGGAGTATAGTGAAATAAGCAACGACGAAAAAACACACACACGCGCTTACACAACATTAGTGAAAACGAAGTAACATTTATTACTTAATTACTATAGTTGAAAAAAGAACCGAAAATAGCAGGCACGACCAACAAAAGCTACTTTCTAACAGACAAGGACATATTAATCAATTTCGAATATTTAGTATTTCCTCTAACACAATAAACACTCCCCCTTCCTTATTGGCCCCTCATTTTCAAAGAGTAAGACAAAAATCAAGAGGAAAATTAACGCCCCACAGTACAATATCATAATATCAAAAAAAAAATTCTACA |
| *P_KmPDC1_* | AGAAAATTAACAAGAGCGAAAAAAAAACGGGCTTCGGTGAAAATCGGGTGAATATGCAACTAGCGGGACGAATGCTCTGGAAATGCATATCCTATGCAACTAGCGGGATGAACAAATCTCACCCCAGAATTCGCAGGAAAAAACAGGAAAAAAAAAAAAGAAGGCCACCACGGCCACAAAGACCACAAAGACCACAAAAAAAAACAAAAAACAACCGTCCCAGCTTCCAGTGTTTTTTTTTTTTTGGAATACTGGAACACAGGAAGCCGCATAAGAGTGGGCGTTGCACAGGAAGCCAGGCCCAGAAGCCCCAGAGTTACTTTTTTTTTTTTTGTTTTTTCCTTCTGTTCGCTGTGCCCGCATCAGATGATGCGCCTTTATTTACGATGCCAATGCGAATAGCACCAGTGAGAGCACCAGTAAAAGCATACGCATACACATACACACATACACACATACACACATACACACATAGAGCAAGCAAGCAGGCTAGCAACCAGGAAGGCTGCCAGTGACTGCTACTGGGTGTCTAAGAACCGTAGGGCGGATTATTGTTGCGGTGGTTGGTTGCGGGTGGTTATGCGATGGTACGGTGCAGAATCGTACGGTGTTGGTTATGGAATTAGTATGGGTATGTGATATGTGGTAATATGTGATATTGGGTTATTGTGATTTGGAATACTGAATATCGAATATGGGATATGGAATATGGCCATGGCATGGTATGGTATGGGATGGGAGTATTCTATTTTATTTTATTTTATTCTGGTTCCTGCGTTTAGGGTAGGGTAGGAAGAAGGTGAGTGCTTTTGTATATAAGTGGAGTGTCTGGATCAGTTTTGTGGATTGTGAATGTTGTTAGTTTCCCCTTTAATGTATATTTGTATTATTTGCTTTTGAGTACTCAATAACCAAGCACAACTACTAGTTTTAAAGGATCCATCCTCTTAAACAGTACAAATCGCAAAGAAAAGCTCCACACCCAAACCAAATAATTGCA |
| *P_KmSNR52_* | TGCCGCAAATAGGGCAGGCGATATACAAGGCGGTATACAGGGCGGTATACAAGGCGTATGCCAGACGGTATGCCTGGAGGCATTGTCAGGAGGCATTGTCAGGAGGCATTGCCGGGCGAAATCGCCAGCTTGAGTATGGCCCCAGAGGGAAGAAAAAACAGTTACCCAGGTGTGTGCCCCTGTATTACCCTGACTTTGAAATGTACGCGAAGTACACTTCGTGCAGGGTGATGATGCTCGTATCAGGTGGACTTGGCGGACACGGTACGGTACGGTCCCCTTTTTTTATTTTTTCTTTTGGGAATGAGAAGGCCACGTGACGTTACCCTGGCCCTGTGTTTCCCCCCTCGCATCTTGTACTGCCTTAGGACCCCTCGTAGTGTAGCGGTCCGGGCCCCCAGATTGCGATGTGTTTCGAGAAAAACAACAAAAAAAATAAGTTGAAAAGCGCGGATCAGGCGGTCCGCCCGGAGTGCGCGCGCTGTATTCTCGCCTGACAGGGCGGGATTCGCAACGTCCGCAGTTCGAATC |
| *P_KmTEF1_* | CCAACGCATATGCTGCAATCACCCAACGGAATTAATTAATTAAGTAGTTACTTACAGTATTGTAATGCCATCCCAGCGTATCCCAGCCTAGTGTATCCCAGCCTAGCCTAGCCTAGGCCAAACCTAGCCCTCTCTAGCCTAGCGCCCAGCAGAAACACCGATGAAGCAAAGAAGTAACAGCAGGAAAGAAAAACAAACACAACAAAAAAAAACAAGCAGCATAGCATCAACAGAAATTTCTAAAGAGAACCAAATTCACCCCAGAAACAACCGCACAAATACGACATCCATCCACCTTTCTTTTATCTTCTTTTTCTGATCTGATAATTAGTTTCATATACAATACGTAGAAACAGGCGCACAGCACCCAGACCTGGCTTCTGCCCCAGTGTATAAGCAATGTAGCATAATTGGAAAAAAAAACGAAAAATACCGAAAATAAGTGGGAAGCTGGGCCACAGGAGTGGGGCGGGATGCGACTGCTTCTGAGCGGGACCGGGTAATAAGGTTGAAAAACTTTGAATTGATGGAATAAGAAACTTCTTTCTTTTCGCTGGCGGGAGAAGGAAAAAAAAAATTTTTTTTTTCCTTCTGTTTAGTACTGGAACATTGAGAAGGCGTGTCAATTTTGAATAATTAGAGTGGTCAAAAAAATTTTTTTTGCTTGGGATACCCTTTTTCGATAATGTAAATTTTTTTTGAATATAAAAGGAGATTGAAAAATTTTTTCTAGCAGAAATGTTTTCAAGTTTTAATTGCAAGTTTCGTTTGAGTATTCAGTTGTATTTTAGTTGATTTGTAGTTTATTTACTAGTATTCTCATAGTTCTAACTCCAAGAGAAGTAACATTAAAG |
| *P_KmtRNA_^GLY^* | TTCCTTTGGAGTCCAGGATTGGTTAGCAACTCTTTGGCCAGATTTCAATAGTTCGATAATTCTTTCAACCAATTGAGCTTCTTCCTTCTTTTGCTTGACTTCCAAAGATTGACCACCTCTTCTAGTGATCTTTTCGATATTTTCCAAGTGCTTTTCAGCGAATTCGATATCCTTCAATCTCAATTCAGTGTTGATGATATCCAAATCACGGACTGGATCAACGTCACCTTCAATGTGAATAATTTCAGCGTCATCGAAACAACGAACGACTTGGTAAATGGAGTCGACAGCTCTGATGTGGGACAAAAAGGCGTTACCCAAACCTTCACCGGCGGAAGCACCTTTAGTCAAACCTGCAATATCGTAGACAGTCAAATGAGCTGGAACCTTGGAAGCTGGTTTGTAGATTTCACTTAATTCCTCGAATCTAGGAGAAGGAACAATAACTCTAGCTTCTTCTGGGTCAATAGTTGCGAATGGGTAGTTAGCTGGGTTACCCAATGGACATCTGGTAATAGCTTGGAAGAAGGTAGACTTACCGACGTTGGCCAAACCAACAATACCAGCCTTCAAGTTATTACCTGGACGACCTAATAAGACCTTCTTTTCTTCGACTTGCTTCTTTGGTGGCATGATTTATACTAACTGTGACAGCCTAGTGAAACTTGAGTTCTTATGTATCAAGAGAAGATGAAAAGTGTAATAGATAAAGACAAAAACAAAAGCTTTCAACAAATAAATACTACAGAAATGATTGAAATTGAAGAAACCTTTAACAGAGCATGATGCTTTAAGTCTTTGCTAATATTTTTTCATTAAGATGGGATGAGGAAACTTTTCAAAAATATTTCGATGACGTATCGCTTCGTACGTTGTTTGTTTAAAATTTGAAATAAAAATAATTGCAAAAAAGA |
| *KmSDH1* | ATGCAATTGTGCAGAAGAAATGCCGCTAGAACTTTTTCTAACAGTGTGGTTTTGAGACACGCATACGCTGCCAAGGACGATGTTGACGGTCAACAGCAAGAAAGTGCGCAAAGCGGACAATACAACATTGTGGACCACGAATACGACTGTGTTGTTGTTGGTGCTGGTGGTGCTGGTTTGCGTGCTGCGTTTGGTCTAGCCGAGGCTGGTTACAAGACTGCTTGTATCTCGAAGTTGTTCCCAACCAGATCGCATACTGTTGCTGCTCAAGGTGGTATCAACGCAGCCTTGGGTAACATGCATAAAGACGACTGGAAATGGCACATGTACGATACCGTGAAGGGTTCCGATTGGTTAGGTGACCAGGACTCTATCCACTACATGACCCGTGAAGCTCCAAAGTCTATCATCGAGTTGGAACACTTTGGTGTGCCATTCTCCAGAACCGAAGAAGGTAGAATCTACCAAAGAGCCTTTGGTGGTCAATCCAAGGAATACGGTAAGGGTGGTCAAGCATACAGAACCTGTGCCGTCGCTGACCGTACCGGTCACGCCTTGTTGCATACCTTGTACGGTCAAGCTTTGAGACACAACACTCATTTCTTCATCGAATTCTTCGCTATGGACCTTTTGACCCACAACGGTGAAGTTGTCGGTGTCATGGCCTACAACCAAGAAGACGGTACGATACATAGATTCCGTGCCCACAAGACAATCATTGCCACTGGTGGTTATGGGCGTGCTTACTTCTCTTGTACCTCCGCTCACACTTGTACCGGTGATGGTTACGCTATGGTCTCCCGTGCCGGTTTCCCATTGCAAGATTTGGAATTCATTCAATTCCATCCATCCGGTATTTACGGTTCCGGTTGTTTGATCACCGAAGGTGCTCGTGGTGAAGGTGGTTTCTTGGTCAACTCCGAAGGTGAACGTTTCATGGAACGTTACGCCCCAACCGCCAAGGATTTGGCTTGTAGAGACGTTGTCTCCCGTGCCATCACCATGGAAATCAGAGAAGGTAGAGGTGTCGGTAAGGAAAAGGACCACATGTTCTTGCAACTAAACCACTTGCCTCCATCCGTCTTGCACGAACGTTTGCCAGGTATCTCTGAAACCGCTGCTATTTTCGCCGGTGTTGATGTTACCAAGGAACCAATCCCAATTCTACCTACCGTCCACTACAACATGGGTGGTATCCCAACCAAGTGGAACGGTGAAGCTTTGACCATCGATGAAGAAACTGGTGAAGATAAGCCAATTCCAGGTTTGCTTGCCTGTGGTGAAGCTGCCTGTGTTTCCGTTCACGGTGCTAACAGATTGGGTGCTAACTCCTTGTTGGATTTGGTCGTCTTTGGTCGTGCCGTTGCTCACACTATTGCTGACACTCTACAACCAGGTTTGCCACACAAGCCTTTGCCAGCTGACTTGGGTAAAGAATCCATTGCTAACTTGGACAAGTTGAGAAACGCTACTGGTTCTAAGCCAACTGCTGAGATCAGATTGGCTATGCAAAAGACTATGCAAAAGGATGTCTCCGTCTTTAGAACACAAGAATCTTTGGACGAAGGTGTTAAGAACATCACCGCCGTCGACAAGACCTTCGCTGATGTCAAGACCACTGACAGATCCATGATCTGGAACTCTGACTTGGTCGAAACCCTTGAATTGCAAAACTTGTTGACTTGTGCTACTCAAACCGCCAAATCTGCTGCTGAAAGAAAGGAATCTCGTGGTGCTCATGCCAGAGAAGATTACCCAGAAAGAGATGATGTTAACTGGATGAAGCACACTCTATCATGGCAACGTGACAGTGGTGACGAAGTTACCTTGAAATACAGAAACGTCATCGCTACCACCTTGGATGAAAAGGAATGTCCTCCTGTTCCACCAACTATCAGAGCCTACTGA |
| *KmSDH2* | ATGTTCAACGTCATATTTAAGAGAGGTTTGGCCACTGAGGTCAAGGCTGCTGCTCCAAGATTAAAGACCTTCAAGATTTACAGATGGAGTCCAGATACCCCAGCTGAAAAGCCACGTTTGCAAGAATACAAGGTCGACTTGAACCAATGTGGTCCTATGGTTTTGGATGCTTTGATCAAGATTAAGAACGAACAAGATCCAACCTTGACTTTCAGAAGATCCTGTAGAGAAGGTATCTGTGGTTCTTGTGCTATGAACATTGGTGGTAGAAACACTTTAGCCTGTTTGTGCAGAATTGATCAAAATGAAAATAAGCAAACTAAGATTTACCCATTGCCTCACATGTACATTGTGAGAGATTTGGTGCCTGACTTGACTCACTTCTACAAGCAATACAAGTCTATCCAACCATATTTACAAAGAGAAAAGGTCCCAGAAGATGGTAAGGAAAACTTGCAAAGTATTGCAGACCGTAAGAAGTTGGATGGTTTGTACGAATGTATCTTGTGTGCTTGTTGTTCCACATCTTGTCCATCTTACTGGTGGAATCAAGAACAATACCTAGGTCCAGCCGTGTTGATGCAAGCTTACCGTTGGTTAATCGACTCTAGAGATGAGGCCTCGGGTATCAGAAAGCAAATGCTTCAAAACTCCATGTCTTTGTACAGATGTCACACAATTATGAACTGTACCAGGACTTGTCCAAAGGGTTTGAACCCAGGTAGAGCCATTGCTGAAATTAAGAAGGCTTTGGCTATGGACTAG |
| *KmSDH3* | ATGTTCAAGAGTGTTTTGAAGCCAGGAATTATCAGCGGGGTCAGACCCAGCGTTTTGAATGTCAACACAAGATTGATGATGACCAACTTTGTTCGTGGTCAATCCAACATTATGGCCAAGAAGACAACCACTGCTGAAGAATACGAGCTACTTGTTGCTCAAAGAAAGGCCAGACCAGTGTCTCCACATTTGACTATCTACCAACCACAATTGACATGGTATTTGTCGTCCGTACACCGTGTTTCTGGTGTTCTTTTGGGTTTTGCATTCTATGCGATTACCATTGGGTTTGGTGTTTCGAGTTTGTTCGGTCTAGGTTTGAACACTGAGAACTTGGTTCAGTTCTACAACGAGAAGATCCCATCGTGGTTGGATATCACTGCTAAGGTTGGTTGTGCGTACCTATTTGCGTTCCATTTCGGTAACGGTATCAGACATTTGGTCTGGGATGCCGGTAAGGAATTGACTTTGAAGGGTGTTTACAGAACCGGGTACTCTGTTTTGGCATTGACTGCCGTGCTAGGTTCCTACTTGCTCACTTTGTGA |
| *KmSDH4A* | ATGTTTAGGTATAGACTGATATCTCCACAAGTTAGAGGGATTAGGCTCCCATCTTTGCCTAATAAAGCTTCTCTATCCAAATACAAGTTAATTCCTCCACCTCCAGGAGGAGTTACTGGAACTGTAAATGATACCTTACCCAAATCAGAACCAAACTGGTTTCATGGTTCGTATCACTGGGACTATGAACGTATTACCGCTGTGTCCCTAATCCCATTAACAATGGTACCACTCTACGGGGCGATGTCATCTGCTACCTTCGCAGCTACATTTCCAGTTCCAATCATCGATGCTGTCTTAGCAAGTACTATACTTATACATTCATATTTGGGTATCACTAGTTGTATTATTGACTATATCCCACTAAGAAAGTTTGGTTTTTGGCATAAGGCAGCAAAGTTTGCCTTGGCCCTTGGAAGTTCAATAAGTCTTTACGGGATCTACGTTTTAGAAACGGAGAATAATGGATTGATAGATTTGATTACAGCTCTTTGGGACAAAGAAAAGGGTGACTCAAGAGCATATTTATTTGAAAGAAGGTACTGA |
| *KmSDH4B* | ATGCTTTCCAGAACTATTAAATTGACTCAAACAAGAGCTTTCCAAACTACCGCCAAGAAGAACTTGACTATCCCATTCTTGTCTACTCTACCACAAGCACCTGGTGGTGTCACAGGCCAGGTCAACGAAGCTTATGTTGCTCCCCCTCCAGAAAAGCTACATGGTTCTTTACACTGGAATTTCGAAAGAGCTCTTGCGATCTCTTTGGTTCCATTAGTGACTGTTCCATTGGCTACTACGGGTTCTATTTCTACCGCTTTAGATGCCACTCTCGCTTCCGTCCTATTGGCTCACTGTTACGTTGGTCTCCAATCATGTATCATTGATTATATCCCAGCTAGAGTTTACGGAAAGAATCATAACTATGCGATGTACTTGCTAGGTATTGGTTCTGTTTTCTCTGCAGTCGGAATCTACCAAATTGAAACAAAGGAAGGTGGTATCATGGGTGTTGTAAAGAACCTCTGGACCAAACCTGCCGAAGAAAAGAAATGA |
| *KmSDH5* | ATGATTGCACCAGGAATTTTGTTACAAGGAGCAAGGGCAGTGCCAAGGGCAGTGCCAAGGACAATGCCACGGGTAGTGTCAGTGGCCAGGCCACTGGCCTGGCCACTGGCAAGACACTTCCATGTGTCGTCCCAGGTCAGCAATGGCAAATCCCAGGACAAATCCCAGGACAAATCCCAGGACAAATCTCAGGACAAATCACCTGCTTCTAGCGAAGCAGGTGAAGCAGCTGATGAAGACGACGTCACCCTCAGAATCAGAATCCCCCCCATCGAGAGAACAGGCGAGTCCCTCGACAGAAAGCGTGCCCGCTTGATCTACCAGTCCCGTAAGCGTGGTATTCTCGAAACGGACTTGCTCTTGTCCCGTTTCGCTGCCAAGTACTTGAAAACCATGTCTCCGGAAGAGTTGCAGGAGTACGACGAGTTGTTGAACGAGCTCGACTGGGACATCTACTACTGGGCCACCGAGAACTACTCCATCACGCCGCTCCCAGACAAGTGGAAGGACTCCAAGATGCTCAAGAGGCTCCAGGAATTCAGCAAGAACAAGGACCGCGAGATCTTGAGCATGCCGGACTTGTCGAAGTATTGA |
| *KmGPD1* | ATGATTCAGTTTGGAAGATCTTTTTGTTACAATAGTATTGCTAAGTCGTTTCAAATCCGTAAATCAATAACCAGATTCAGATTAACTGACATCTACACTAATAGAGTAACAATCAACACAAGAAGAAATATTTCCAATATGAGTCCAGCTTCCGATAGATTAAAGCAAACTTCATCCATTCTTTCCAAGTCTGTTGAGCCAAAGGCCGACAGCCCATTCAAGGTCACTGTTATCGGTTCCGGTAACTGGGGTACTACAATTGCTAAGGTTGTTGCTGAAAACTGTGCTTTGAGACCAAACTTGTTTGTCAAGAGAGTTGATATGTGGGTTTTCGAAGAACAAATTGACGGCGAAAAGTTGACCGAAATCATCAACACACGTCACGAAAACGTCAAGTATCTACCGGGTATCAAGCTGCCTAACAACTTGGTTGCCAACCCAGACATTGTTGATGCTGCTAAGGATGCTGATATTCTAGTGTTTAACATTCCTCATCAATTTTTGCCAAAGGTCTGCTCTCAACTAAAGGGTAAGATTAAGCCTCAAGCTCGTGCTATTTCCTGTTTGAAGGGTTTCGACGTCGGTAAGGACGGTGTCAAGCTATTGTCTACATACATCAAGGACACTTTGAACATTGAGTGTGGTGCTCTATCCGGTGCTAACTTGGCTCCAGAAGTTGCTAAGGAAAACTGGTCCGAGACCACTGTTGCATACCAAATTCCAGAAGATTACAAGGGCGCTGGTAAGGACGTCGACCACTCTCTATTAAAGGCTTTGTTCCACAGACCATACTTCCACGTTAATGTCATTCACGATGTTGCCGGTATTTCCGTTGCCGGTGCTCTAAAGAACGTTATTGCATTGGGTTGTGGTTACGTTGAAGGTCTAGGATGGGGTAACAATGCTAGTGCTGCTATCCAAAGAGTTGGTCTATCAGAAATGATCACTTTCGGACGTATGTTCTTCCCAGAATGTAGGGTCGAAACCTTCTACAAGGAATCCGCCGGTGTCGCTGATTTGATCACAACCTGTGCTGGTGGTAGAAACGTCAGAGTTGCTAAGCACATGGCCATCACAGGTAAGTCCGCTTTGGAAGCCGAAAAGGAACTATTGAACGGTCAATCTGCACAAGGTATCATCACTACAAAGGAAGTACATGAATGGCTAGAAACTTGCGGTAAGATCAACGAATTCCCATTATTCGAAGCTATTTACCAAATCACATACGGTAACGCATCTATGGAACAAATTCCAGAGATGATTGAAGAATTGGAATGCATTGATTACAACGTCAAGAAGCACTAA |
| *KmACH1* | ATGACAGTTTCTAGATTGTTGAAAGAGAGAGTGAGATATGCGCCATATTTGAAGAAGGTGCGTCCTGTGGAAGAACTGATTCCATTGTTTAAGAACGGACAATACATTGGGTGGTCTGGTTTCACCGGTGTGGGTGCTCCAAAAGCTGTTCCTGAGGCATTGATCAAGCATGTTGAGGAAAATAACTTGCAAGGGAAGCTTAGATTCAACCTCTTTGTGGGTGCTTCTGCTGGACCAGAAGAATGTAAGTGGGCAGAACACGATATGATTATTAGAAGAGCACCACATCAAGTGGGTAAGCCAATTGCCAAGGCTATCAACAACGGTCAGATCCAATTTTTCGACAAGCATCTTTCGATGTTCCCCCAGGATTTGACCTACGGTTACTACACGAGGAACAGAACGGACGGTAAGATCTTGGATTACACAATTATCGAGGCCACTGCCATTAAGGAAGATGGTTCGATTGTGCCAGGTCCTTCTGTGGGTGGGTCTCCAGAATTCATTGCCGTTTCGGATAAGGTCATCATCGAAGTTAACACAGCAACCCCTTCGTTCGAAGGTTTGCACGACATCGATATGCCAGTGAACCCACCATACAGACAGCCTTACCCATACACTAGAGTCGACCAAAAGAGCGGTCTTGATTCGATCCCAGTCGACCCAGAACGTGTCATTGCCGTGGTTGAATCGACTGAAAGAGACAAGGTTGGGCCAAACACCCCATCTGACGCTGTGTCTCAGTCCATTGCTCACAATTTGGTGGAATTCTTCGAAAACGAAGTTAGAGCCGGCAGAATGCCAGAAAACTTGCATCCTTTGCAATCCGGTATCGGTAACATCGCTAACGCTGTCATTGAAGGTTTGACCGACTCCTCGTTCAAAGACTTGACCGTGTGGACCGAAGTCTTGCAAGATTCGTTCTTGGACTTGTTCGAGAGCGGTGCTTTGACCTACGCTACAGCAACCTCCGTTAGACTCACGGACGCTGGGTTCGACAAGTTCTTCGCCAACTGGGACGATTTCTCAAAGAAGTTGTGCTTGAGATCCCAAGTGGTCTCCAACAACCCTGAGTTGATCCGTCGTTTGGGTGTCATCGCCATGAACACCCCTGTGGAAGTGGACATTTACGCTCACGCTAACTCCACAAACGTGTCCGGTTCCCGTATGTTGAACGGTTTGGGTGGTTCTGCCGACTTCTTGAGAAACGCCAAGTTGTCCGTCATGCACACCCCATCAGCAAGACCAACAAAGACTGACCCTACCGGTATCTCTACCATCGTCCCAATGGCTTCTCATGTCGACCAAACCGAACACGACTTGGATGTCTTGGTCACCGAACAAGGTCTAGCTGACTTGAGAGGTCTCTCGCCAAGAGAAAGAGCCAGAGAAATCATCAAGCAATGTGCCCACCCAGATTACAAGCCAATCTTGACCGATTACTTGGACAGAGCCGAGCATTATGCAAAGCTCCACGGATGCTTGCACGAACCTCACATGTTGCAAAATGCCTTCAAGTTCCACTTGAACTTGAGCGAAAAGGGTACCATGAAGGTCGACAAATGGGATTAA |
| *KmADH1* | ATGGCTATTCCAGAAACTCAAAAGGGTGTTATCTTCTACGAAAACGGTGGTGAGTTGCAATACAAGGACATTCCAGTTCCAAAGCCAAAGCCAAACGAACTTTTGATCAACGTTAAGTACTCTGGTGTGTGTCACACCGATTTGCACGCATGGCAAGGTGACTGGCCATTGGACACCAAGTTGCCATTGGTGGGTGGTCACGAAGGTGCTGGTATTGTTGTTGCCATGGGTGAGAACGTTACTGGCTGGGAAATCGGTGACTATGCTGGTATCAAGTGGTTGAACGGTTCCTGTATGTCTTGTGAGGAGTGTGAGTTGTCGAACGAACCAAACTGTCCAAAGGCCGACTTGTCTGGTTACACACACGACGGTTCTTTCCAACAATACGCTACCGCTGACGCTGTCCAGGCTGCCAGAATTCCAAAGAACGTCGACTTGGCCGAGGTTGCCCCAATCTTGTGTGCCGGTGTTACCGTGTACAAGGCTTTGAAGTCTGCTCACATCAAGGCTGGTGACTGGGTCGCCATCTCTGGTGCATGTGGTGGTCTAGGTTCCTTGGCCATCCAATACGCCAAGGCTATGGGTTACAGAGTGCTAGGTATCGATGCTGGTGACGAAAAGGCCAAATTGTTCAAGGAATTGGGCGGTGAATACTTTATCGACTTTACCAAGACCAAGGATATGGTAGCAGAAGTCATTGAGGCTACCAACGGTGGTGCCCACGCCGTCATTAACGTGTCTGTGTCCGAAGCCGCCATCTCTACCTCTGTCTTGTACACCAGATCAAACGGTACCGTCGTCTTGGTCGGTTTGCCAAGAGACGCTCAATGTAAGTCTGATGTCTTCAACCAAGTCGTCAAGTCCATCTCCATTGTTGGTTCTTACGTTGGTAACAGAGCAGACACCAGAGAAGCCCTAGACTTCTTCTCCAGAGGTTTGGTCAAGGCCCCAATTAAGATTCTCGGCTTGTCCGAATTGGCAACCGTTTACGACAAGATGTCCAAGGGCCAAATCATTGGTAGAATTGTCGTTGACACTTCCAAATAA |
| *KmADH2A* | ATGTCTATTCCAACTACTCAAAAGGGTGTTATCTTCTACGAAAACGGTGGTCAATTGTACTACAAGGACATCCCAGTCCCAAAGCCAAAGTCTAACGAACTTTTGATCAACGTTAAGTACTCCGGTGTCTGCCACACCGATTTGCACGCCTGGAAGGGTGACTGGCCATTGGACACCAAGTTGCCATTGGTCGGTGGTCACGAAGGTGCCGGTGTCGTCGTCGCCATGGGTGACAACGTCAAGGGCTGGAAGATCGGTGACCTTGCCGGTATCAAATGGTTGAACGGTTCTTGTATGAACTGTGAAGAATGTGAATTGTCCAACGAATCCAACTGTCCAGACGCTGACTTGTCCGGTTACACCCACGACGGTTCTTTCCAACAATACGCTACCGCTGACGCTGTCCAAGCCGCTCACATCCCAGCTGGTACCGACTTGGCTCAAGTCGCCCCAATCTTGTGTGCCGGTGTTACCGTCTACAAGGCTTTGAAGACCGCTGAAATGAAGGCTGGTGACTGGGTCGCCATCTCCGGTGCTGCTGGTGGTCTAGGTTCCTTGGCCGTCCAATACGCCAAGGCCATGGGTTTCAGAGTCCTAGGTATCGATGGTGGTGAAGGTAAGGAAGAATTGTTCAAGAGCTTGGGTGGTGAAGTCTTCATTGATTTCACCAAGTCTAAGGACATTGTCGGTGAAGTCATCAAGGCTACCAACGGTGGTGCTCACGGTGTCATCAACGTCTCCGTCTCCGAAAAGGCCATCGAATCCTCCATCGAATACTGTAGATCCAACGGTACCGTCGTTCTAGTCGGTTTGCCAAAGGACGCCAAGTGTAAGTCCGATGTCTTCAACCAAGTCGTTAAGTCCATCCACATCGTTGGTTCTTACGTCGGTAACAGAGCTGACACCAGAGAAGCTCTTGACTTCTTCTGCAGAGGTCTAGTCCACGCCCCAATCAAGGTTGTCGGTTTGTCCACCTTGCCAGAAATTTACGAAAAGATGGAACAAGGTAAGATTCTAGGTAGATACGTTGTTGACACTTCCAAATAA |
| *KmADH2B* | ATGTTTCGTAAGGTCACATCTGTTGCAGCAGCAAATTTAGGACGTTCTTTCAGCTCAAGTCCCATCAATCTCTCAGCTCAAGCCAAAATGATGAAAGCTATGGTATATTATGGTGCGAACAACTTGAAATTTGAAAATAGGGCTGTTCCAAAAATTATTGACCCAACAGATGCTATTATTAAGATGACCCAAACTTCTATATGCGGTACGGATCTCGGTATTTGGAAGGGTAAGAACCCTGAAATCGAGGAGATTGCAGAAGCGAAGGAAGGAAAGTTCAATGGAAGGGTATTAGGCCATGAAGGTATCGGTATTGTTGACGAAGTCGGCGCTGGAGTTCGTAATGTGAAGAAGGGTGACAGGGTGATTATCTCTTGTATTACAAGATGTGGTATCTGTGAGAATTGTTCAAGATCAATGTACTCTCACTGTAATAGCGGAGGTGGTTGGCTCTTGGGATATATGATTGACGGAACACATGCTGAATATGTCCGTACCCCATTTGCCGACACCTCTCTATACAAGATTCCTCAAGGTCTATCTGATGAGGCTGCCGTGTTGCTTTCTGATGCTTTACCTACTGCCCATGAAATTGGTGTGCAAAATGGTAATGTTAAACCTGGTGACACAGTAGCAGTGATTGGAGCAGGTCCTGTCGGTATGAGTTGCGTTTTGACATCACAATTGTACTCTCCAAGTGTTCTAATCGCGGTGGATATGGATGATAACCGTTTGGCTATGGCTAAGGAGATGGGTGCTACTCATACGATAAATTCTGCAAAAGAGGATGCTGTGAGTAAGATCTTAGAGTACACGGAAGGTCGTGGTGTTGATTGCGCAATGGAAGCCGTGGGTGCCCAGCCAACTTGGGATATATGCCAACGTGTTCTCAAGGAAGGTGGTCATTTAGCAAACGTCGGTGTCCATGGAAAGTCTGTCAATCTTGAGATTGAAAAATTGTGGATAAAGAATTTGACCATCACTACTGGATTAGTGAACACCAATACCACTGAAATGTTAATGAAGAATTGTTGTTCTGGAAAGTTGAAGTCCGAAAAGCTTGCCACTCATCAATGCAAGTTTGAAGATATGGAAAACAATTACACTGTATTCAAGCACGCTGCTGAAGAAAAGGCTATGAAACTTATTATTAACTTTTAG |
| *KmADH3* | ATGCTTAGATTAACTAACGCCAGAACATTTGTCAAGCCATTGCGCGCCACTGCATTCGGTTCCTTCAGAACGATGGCCTCTGTTGCTATTCCAGAAAAGCAAAAGGGTGTTATTTTCTACGAAAATGGTGGTAAACTAGAATACAAGGACATTCCAGTTCCAAAGCCAAAGCCAAACGAAATCTTGATCAACGTCAAGTACTCTGGTGTGTGTCACACAGATTTGCACGCCTGGAAGGGTGACTGGCCATTGGCCACCAAGTTGCCTTTGGTCGGTGGTCACGAAGGTGCCGGTGTCGTTGTCGCCATGGGTGAAAACGTTAAGGGCTGGGAAATCGGTGACTATGCCGGTATCAAGTGGTTGAACGGTTCTTGTATGTCCTGTGAATTCTGTGAATTGTCCAACGAATCTAACTGTCCAGACGCCGACTTGTCCGGTTACACCCACGACGGTTCCTTCCAACAATACGCTACTGCAGATGCTGTGCAAGCTGCAAGAATTCCAAAGGGTACCGATTTGGCTGAAATCGCCCCAATCTTGTGTGCTGGTGTTACCGTCTACAAGGCTTTAAAGACCGCTGGCTTGAAGGCTGGTGACTGGGTCGCCATCTCTGGTGCCGCTGGTGGTCTAGGTTCCTTGGCTGTCCAATACGCCAAGGCAATGGGTTACAGAGTTGTCGGTATTGATGGTGGTGAAGAAAAGGGTAAGTTGGCCAAGCAATTGGGTGCCGAAGCCTTCGTTGACTTCACCAAGACCAAGGACATGATTGGTGAAATCCAAGAAATCACCAACGGTGGTCCACACGGTGTCATCAACGTCTCCGTCTCCGAAGCCGCCATGAACGCTTCCACCCAATACGTCAGACCAACTGGTACTGTCGTGTTGGTTGGTTTGCCAGCCGGTGCAGTCATCAAGTCTGAGGTCTTCTCCCACGTCGTTAAGTCCATTGCCATCAAGGGTTCTTACGTCGGTAACAGAGCTGACACCAGAGAAGCCATTGAGTTCTTCGCTGCTGGTAAGGTTAAGTCTCCAATTAAGGTTGTTGGCTTGTCTGAATTGCCAAAGGTTTACGAACTAATGGAACAAGGTAAGATCTTGGGTAGATACGTCGTCGACACTGAAAAATAA |
| *KmNDE1* | ATGTTTGTGAACAAGCATCTAATGGCTGCTGTTGCCCGTAACAGCAGCAGAGCTTTGAACGTTTCTGCTCGCTCTGGCACCACCGCCAGATTGTTTTCTACATCAAGACCAGCCTTCAATGCTGCTGCTGGTAAGCCTTCCTTGGCCAAGAGAGTTTTGAAGGGTACTTTGAAAACCTCTTTGGTTGCCTTGCTTGCAGGTACTGCTTATGTCTCTTATGAATTATACAGGGAGGCTAACCCACCTCCACAAGTTCCACAATCTCCAACTTTCAGCAATGGATCTCCAAGAAAGACCCTAGTCGTCTTGGGTACCGGTTGGGGTTCCGTCTCGCTATTGAAGAACTTGGACACCACCTTGTACAACGTTATTGTCGTTTCTCCAAGAAACTACTTTTTGTTCACTCCCTTATTGCCATCTACCCCCGTCGGTACTGTTGAATTGAAGTCTATTGTCCAACCTGTTAGAACTATCACCAGATCTTCCCCAGGTGAAGTCCACTACTACGAAGCTGAAGCCAAGGATGTCGACCCTGTTGCCAAGACCGTCAGAATCAAGTCTGCTACCAAGGACCACGATTACGAATTGGACTTGAAGTACGACTACTTGGTCGTCGGTGTCGGTGCTCAGCCAACTACCTTTGGTATCCCAGGTGTGTTTGAAAATGCTTCCTTCTTGAAGGAAATCCCTGACGCTCAAGACATTAGAACTAAGATTATGAACAACATCGAAAAGGCCGCTACCCTATCTCCAAATGACCCAGAACGTAAGAGATTGTTGAGCTTTGTTGTTGTTGGTGGTGGTCCAACCGGTGTTGAATTCGCTGCTGAATTGCAAGACTACGTTGACCAAGATTTGTCTAAATGGATCCCAGAAATCTCTAAAGAAATTAAGGTCACTTTGGTTGAAGCTCTTCCAAACATTTTGAACATGTTCGACAAGTCTCTATGGCAATACGCCCAAGATTTGTTCGCTAAGGAAAAGATTGACTTGAAATTGCAAACTATGGTTAAGAACGTTGACTCCACTACCATTACCGCCAAGCGTGGTGATGCTGTCGAAGAAATTCCATATGGTGTTTTAGTCTGGGCTACCGGTAACGCTCCAAGAGAAGTCTCCAAGAACTTGATGCAAAAGCTACCTGAACAAAATTCCAGACGTGGTCTTCTAATTAACGACAAGTTGCAACTATTGGGTGCTGAAGACTCTATCTTCGCTATTGGTGATTGTACCTTCTACCCAGGTCTTTTCCCAACCGCCCAAGTCGCTCACCAAGAAGCAGAATACTTGGCTACCACTTTGAAGACCCAGTACAAGATTGACCAATTGAAGTGGCAAATCGCTAACACTAGCAACGCTACTGAAACTTCTAAATTGCACTCCAAGTTGGACAGATTGTCAAAGCAAATTCAACCATTCAAGTATGTTCACCAAGGTACCCTTGCCTACATTGGTTCTGAACAAGCTATCGCTGACCTACCTTTCGGTGACTCCAAGTACCGTATGGCTGGTTCATTCACTTTCTTGTTCTGGAAGTCTGCTTACCTAGCCATGTGTCTATCCTTCAGAAACAGAATTTTGGTGTGTATGGACTGGGCTAAGGTTTACTTCCTAGGAAGAGATTCTTCTGTTTAA |
| *KmRAD52* | CTATGAAATGTTTTTCCTGATGTTCGGCTGCTGGAGCATACGTGGTCTGCCTAATTGTGTACGTTGTGCTGCCGGTCTCGCATTTGATGCTGTATTTGGTGCTAGCTGAGCAGGATATCTTGTTGGTGTAGTCTTGTTAGTTTCTGTTGACGATTGAGAAGTGGCAGCCTGCGCTTCATTTGTTGTATTCGTAGATAGTTCTTTTCCCTTTGGGGCAAAGCTAGAGTATATTTTCGATTTATTCGTATCGATTCCTTTTTCTTTCAGGACAGTTGCCCGTACGGGTATGGATACTGATTGATCTACCGTGTGTCTTATTGACTGAGCCTGAAATTTTGGATCGAATATGGCATCCGATGAGATTGGACTTTTGTTCTGTAAAGATTCCGCTGCTTTTGCTGTAACGAATGTCAGCGGTGATGTAGTTCTTGGTTCTTCGGTAGAACCGGAGTTTGATTTTGCATTCATATTAATGAGGTCTTCTTCTTGTATTTCATCACTAAACATGAAAGAGTCATCAAGCAAGTCGTCCGGATCTTGTGCAGCTTCTTCATTTGAAACAGGGTTTACTGCTGCAGTTGTGGTCACAGGGGCAACCGCCGTTGTCGAGTTTGTTGCAGATGGCGCCTCTATAGTTACAGATGCTGTTGGGCGGTTCTGATTTATTGATGCAGTTGATGATACTACTGGTGCTCCTGGTTTTTGAATAGATACATGAGACTGATTTTTTGGGTCATGGTTTAAAATACGCTTCTTCAGCGACGGTCCCTCTGTATGGGAATCTGCTATAGTGTTTGATCGTGTTATTTCACTAAGTTCGTCAGCAGGTCTGAAGAGATTCCCTTCATCGAAATCTGGTGGATCAAACTTCACTTTATCGATCTTAGCTAGGAAATCTTTATCATAAAGACAGTTACCAAGAGCATTTCCGAATCCCCTAAGGGATCTCTTTAATGCATCGGTGACTGCTGACTTTTTAGCACGTTCAAACGCACTCGCCTTCCGACGTTCATTCTCCACTGTCCCATATCCAATATCCTCTCTGAACGTGCCATCTGCCAGCGAAACGCGCACTATGGCAGTACATCCAATGCAAAACTTTCCCTGTCTTTCATCAAGAAAATCCACAGTCACATTCTTAACCTCCGTAGACCATCCATTATATCCAAATATCTGGTTTGCTAGATTAATAGCCTTCCATCCTTCAATGTAAGCTACTCTACTTGACCCAAATCCAATCCTTTTAGAAATATATTCTGGCCCAAGTTTCTTATCTAATTTACTCTGGATATCATCCTGAGCACTCTTCGCGCCACTGTTTCCCTGCCCATCATCCAT |

**Table S5 Compare the CRISPR gene-editing efficiencies among different yeasts**

| Strain | editing method | Targeted site | Donor DNA homologous arm length（bp） | Editing efficiency | literature |
| --- | --- | --- | --- | --- | --- |
| *S. cerevisiae* | CRISPR/Cas9 | *TRP1* | 35 | 50-52% |  |
| *S. cerevisiae* | pop-in/pop-out（PIPO） | *HTB2* | 70 | 55% |  |
| *S. cerevisiae* | pop-in/pop-out（PIPO） | *HTB2* | 140 | 79% |  |
| *Y. lipolytica* | CRISPR/Cas9 | *XRP2* | 50 | 5-9% |  |
| *Y. lipolytica* | CRISPR/Cas9 | *PEX10* | 1000 | 78% |  |
| *Y. lipolytica* | Ura3-blaster | *ADE2* | 1000 | 90-95% |  |
| *Pichia pastoris* | CRISPR/Cas9 | *GUT1* | 1000 | 77.50% |  |
| *S. stipitis* | CRISPR/Cas9 | *XYL2* | 50 | 70-90% |  |
| *fission yeast* | CRISPR/Cas12a | *ADE6* | 200-500 | 60-80% |  |
| *O.polymorpha DL-1* | CRISPR/Cas12a | *HIS4* | 500 | 96.2±1.9% |  |
| *K lactis* | CRISPR/Cas9 | *URA3* | 1000 bp | 55.90% |  |
| *K lactis* | CRISPR/Cas9 | *ADE2* | 1000 bp | 81% |  |
| *K. marxianus* | CRISPR/Cas9 | *URA3* | 50 | 84-92 |  |
| *K. marxianus* | SlugCas9/HF | *GAP1* | 500 | 26-86 |  |
| *K. marxianus* | SlugCas9/HF | *ADE2* | 500 | 58.7-76.8 |  |
| *K. marxianus* | CRISPR/Cas9 | *ADE2* | 40 | 80-98 |  |
| *K. marxianus* | CRISPR/Cas9 | *ZWF1* | 40 | 60 |  |
| *K. marxianus* | CRISPR/Cas9 | *GPD1* | 40 | 95 |  |
| *K. marxianus* | CRISPR/Cas9 | *DNL4* | 85 | 50 |  |
| *K. marxianus* | CRISPR/Cas9 | *LAC4* | 880 | 100 |  |
| *K. marxianus* | CRISPR/Cas9 | *PHO13* | 60-308 | 25-50 |  |
| *K. marxianus* | CRISPR/Cas9 | *ADE2* | 480 | 96 |  |
| *K. marxianus* | CRISPR/Cas12a | *ADE2* | 35 | 66.67 | This study |
| *K. marxianus* | CRISPR/Cas12a | *ADE2* | 40 | 89.45 | This study |
| *K. marxianus* | CRISPR/Cas12a | *ADE2* | 50 | 92.26 | This study |
| *K. marxianus* | CRISPR/Cas12a | *ADE2* | 60 | 50-100 | This study |
| *K. marxianus* | CRISPR/Cas12a | *XYL2* | 60 | 62.5-87.5 | This study |
| *K. marxianus* | CRISPR/Cas12a | *XYL1* | 60 | 76.9 | This study |
| *K. marxianus* | CRISPR/Cas12a | *TRP1* | 60 | 81.25 | This study |

**References**
